# Germ cell-specific gene 2 accelerates cell cycle in epithelial ovarian cancer by inhibiting GSK3α-p27 cascade

**DOI:** 10.1007/s10735-024-10185-6

**Published:** 2024-04-13

**Authors:** Keyu Zhu, Xiaolu Ma, Xiaolin Guan, Ying Tong, Suhong Xie, Yanchun Wang, Hui Zheng, Lin Guo, Renquan Lu

**Affiliations:** 1 Department of Clinical Laboratory, Fudan University Shanghai Cancer Center, No.270, Dong’An Road, Xuhui District, Shanghai, 200032 China; 2 Department of Oncology, Shanghai Medical College, Fudan University, Shanghai, China

**Keywords:** Epithelial ovarian cancer, GSG2, GSK3α, Cell cycle

## Abstract

Epithelial ovarian cancer (EOC) is one of the most common malignant gynecological tumors with rapid growth potential and poor prognosis, however, the molecular mechanism underlying its outgrowth remained elusive. Germ cell-specific gene 2 (GSG2) was previously reported to be highly expressed in ovarian cancer and was essential for the growth of EOC. In this study, GSG2-knockdown cells and GSG2-overexpress cells were established through lentivirus-mediated transfection with Human ovarian cancer cells HO8910 and SKOV3. Knockdown of GSG2 inhibited cell proliferation and induced G2/M phase arrest in EOC. Interestingly, the expression of p27, a well-known regulator of the cell cycle showed a most significant increase after GSG2 knockdown. Further phosphorylation-protein array demonstrated the phosphorylation of GSK3αSer21 decreased in GSG2-knockdown cells to the most extent. Notably, inhibiting GSK3α activity effectively rescued GSG2 knockdown’s suppression on cell cycle as well as p27 expression in EOC. Our study substantiates that GSG2 is able to phosphorylate GSK3α at Ser21 and then leads to the reduction of p27 expression, resulting in cell cycle acceleration and cell proliferation promotion. Thus, GSG2 may have the potential to become a promising target in EOC.

**Supplementary Information:**

The online version contains supplementary material available at 10.1007/s10735-024-10185-6.

## Introduction

As one of the most common malignant tumors in female reproductive system, the morbidity of ovarian cancer is in the second place and the mortality of ovarian cancer takes the first place. It is estimated that there will be approximately 19,710 new cases of ovarian cancer diagnosed and 13,270 ovarian cancer deaths in the United States in 2023 (Siegel et al. 2023). Epithelial ovarian cancer (EOC) accounts for about 90% of all malignant ovarian tumors (Torre et al. 2018). Unfortunately, most ovarian cancer patients are diagnosed at an advanced stage and the poor five-year survival rate is less than 50% due to lacking of specific symptoms and sensitive biological markers for early diagnosis, early and easy invasion and metastasis and low chemotherapy sensitivity(Armstrong et al. 2022). The primary standard treatment, cytoreductive surgery and adjuvant chemotherapy with a platinum and paclitaxel regimen, can achieve good initial response rates, but the majority of ovarian cancer patients eventually relapse (Chiang et al. 2021). Thus, it is important to identify new therapeutic targets for epithelial ovarian cancer in the era of precision medicine.

Germ cell-specific gene-2(GSG2), also known as haploid germ cell-specific nuclear protein kinase (haspin) is an atypical serine/threonine-protein kinase that is present in all major eukaryotic phyla (Higgins 2001). GSG2 was first discovered in male mouse germ cells (Tanaka et al. 1994, 1999), and has long been deemed to an inactive pseudo kinase on account of its low structural homology with classical protein kinases (Kestav et al. 2017). GSG2 has responsibility for the normal alignment of chromosomes, participation in the regulation of kinetochore assembly and contribution to the formation of bipolar spindles (Dai et al. 2006). In addition, GSG2 is crucial for the phosphorylation of histones, particularly histone H3 at Thr3 (Wang et al. 2010), and this specific phosphorylation serves as a docking site for the chromosome passenger complex at the centromere during mitosis and is critical for centromeric functions of Aurora B (Dai et al. 2006). In mammalian cells, phosphorylation of histone H3 at Thr3 begins at late G2 and runs through anaphase and concentrates on the internal centromeric region of mitotic chromosomes (Dai et al. 2009) and hence GSG2 has a great potential as an anticancer therapeutic target. Although it has been reported that GSG2 is highly expressed in ovarian cancer (Huang et al. 2021), the mechanism of GSG2 promoting the development and progress of ovarian cancer has not been clarified.

In this study, we aimed to explore the molecular mechanism of GSG2 promoting the development of EOC. It is found that inhibition of GSG2 with short hairpin RNA generated a substantially increased expression of p27 and cell cycle arrest. Besides, GSG2 mediated GSK3α to modulate cell cycle and promote epithelial ovarian cancer cell proliferation. Thus, GSG2 is considered to be a potential therapeutic target in epithelial ovarian cancer.

## Materials and methods

### Cell culture

Human ovarian cancer cells HO8910 and SKOV3 were cultured in RPMI-1640 medium (Gibco, USA), containing 10% fetal bovine serum (Gibco, USA) and 1% penicillin–streptomycin (Gibco, USA). HEK 293 T cells were cultured in DMEM (Gibco, USA) containing 10% fetal bovine serum (Gibco, USA) and 1% penicillin–streptomycin (Gibco, USA). All cells were cultured at 5% CO_2_ in a 37 °C incubator. Human ovarian cancer cells HO8910 and SKOV3 were gifted from Professor Renquan Lu from Department of Clinical Laboratory in Fudan University Shanghai Cancer Center HEK 293 T cells were purchased from the Chinese Academy of Sciences Committee (Shanghai, China).

### Inhibitors

BRD0705 (HY-116830, MCE, China) was used in this study as a GSK3α inhibitor which was dissolved in DMSO at a concentration of 10 mM and divided into partial shipments for storage. The cells were treated with concentration of 10 μM and 20 μM for 24 h, and the cells treated with DMSO only were served as control.

**Lentivirus infection and transient transfection** A specific RNA interference sequence was first designed for GSG2 lentivirus expressing. The nucleotide sequences were cloned into the AgeI and EcoRI sites of the BR-V108 vector (YBR, China) to generate the BR-V108-GSG2 (shGSG2) and BR-V108-control (shCtrl) (Table 1) recombinant vectors. The lentivirus recombinant vectors packaged with the packing plasmid psPAX2 and the envelop vector pMD2.G were in proportion of 4:3:2 transfected into 293 T cells using Lipofectamine 2000 transfection reagent (Invitrogen). Then culture medium containing lentivirus particles was collected after 48 h. Ovarian cancer cells were transfected with the above lentivirus and 10 mg/ml Polybrene (YEASON, China) was added to the cells for 24 h. Stably transduced cells labeled with GFP positive were selected using fluorescence-activated cell sorting (FACS), and the RNAi knockdown efficiency was detected by western blotting.

**Table 1:** Sequence of shRNA against GSG2 for transfection

Name	Sequence
shCtrl	5′-TTCTCCGAACGTGTCACGT-3′
shGSG2	5′-CCACAGGACAATGCTGAACTT-3′
siCtrl	5′-UUC UCC GAA CGU GUC ACG UTT-3′
SiGSG2-1	5′-GUGACGGUGACUACCAGUUUGTT-3′
SiGSG2-2	5′-CCACAGGACAAUGCUGAACUUTT-3′
SiGSG2-3	5′-CCCUCCUAUCAGAAUGUUCAATT-3′

Exogenous GSG2 was overexpressed by the lentivirus. DNA was extracted and purified from HOSEpiC cell line by TIANamp Genomic DNA Kit (TIANGEN, China). GSG2 fragment product was amplified by PCR, verified and recovered by nucleic acid electrophoresis and QIAquick Gel Extraction Kit (QIAGEN, Germany). At the same time, the pCDH-CMV-MCS-EF1-Puro plasmid (SBI, Palo Alto, CA, USA) was subjected to enzyme digestion at NheI and NotI sites. After the enzyme digestion product and PCR product were connected, they were added into the DH5α Chemically Competent Cell (YEASEN, China) to activate and the overexpress vector was extracted by TIANprep Midi Plasmid Kit (TIANGEN, China). The following lentiviral transfection is similar to above.

A suite of small interfering RNA purchased from Sangon Biotech (Shanghai, China) was used to reduce GSG2 expression. The cells were seeded in 6-well plates and when cells had grown to 60–90%, 20 pmol siRNA and 6 μl RNATransMate (E607402, Sangon Biotech, China) were respectively mixed with serum-free medium, and then the two were mixed for 5–10 min. The cells were transfected with the above mixture for 24 h and the RNAi knockdown efficiency was detected by western blotting. The siRNA sequences are shown in Table 1.

### Quantitative real-time PCR (qRT-PCR)

Cultured cells were collected to extract RNA using RNA-Quick Purification Kit (ES Science, China) according to manufacturer’s instructions. The isolated RNA is reversely transcribed into cDNA using PrimeScript RT Master Mix (Takara, Japan) and then detected by SYBR-Green real-time PCR assays with qPCR SYBR Green Master Mix (11184ES08, YEASEN, China). The PCR reaction conditions were shown in Table 2. An ABI PRISM Detection System (Applied Biosystems, Life Technologies) was used for testing. The mRNA expression levels were normalized to those of GAPDH, and the fold-change of mRNA levels was calculated using the 2^−ΔΔCT^ method. The specific primers used are listed in Table 3.

**Table 2:** Real-time qPCR reaction condition

Reagents	Volume/ul
qPCR SYBR Green Master Mix	10
F-Primer	1
G-Primer	1
DNA(100 ng/ul)	2
ddH_2_O	6

**Table 3:** Primer sequences for real-time qPCR

Primers	Sequence (5′-3′)
GAPDH-F	GGAGCGAGATCCCTCCAAAAT
GAPDH-R	GGCTGTTGTCATACTTCTCATGG
GSG2-F	GTTTACCGGTGACGGTGACT
GSG2-R	CAGAGCAAGTCAGTGGCAGA
cyclin A-F	GAGGTCCCGATGCTTGTCAG
cyclin A-R	GTTAGCAGCCCTAGCACTGTC
cyclin B-F	AATAAGGCGAAGATCAACATGGC
cyclin B-R	TTTGTTACCAATGTCCCCAAGAG
CDK1-F	AAACTACAGGTCAAGTGGTAGCC
CDK1-R	CCTGCATAAGCACATCCTGA
P21-F	ACCGAGACACCACTGGAGGG
P21-R	CCTGCCTCCTCCCAACTCATC
P27-F	CCTCCTCCAAGACAAACAGCG
P27-R	GGGCATTCAGAGCGGGATT

### Western blotting.

Western blot was performed according to a previous publication (Zhang et al. 2017). The antibodies and the dilution used in the detection were as follows: GSG2 (NBP1-26,626, 1:3000 dilution, NOVUS, USA), GAPDH (10,494-1-AP, 1:5000 dilution, Proteintech Group, China), GSK-3α/β (5676, 1:1000 dilution, CST, MA, USA), phospho-GSK-3α(Ser21) (8452, 1:1000 dilution, CST, MA, USA), phospho -GSK-3β(Ser9) (5558, 1:1000 dilution, CST, MA, USA), p27(25,614-1-AP, 1:1000 dilution, Proteintech Group, China), p21(10,355-1-AP, 1:1000 dilution, Proteintech Group, China), cdc2(9116,1:1000dilution, CST, MA, USA), phospho-cdc2 (Tyr15) (4539,1:1000dilution, CST, MA, USA), cyclin A(4656,1:1000dilution, CST, MA, USA), cyclin B(12,231,1:1000dilution, CST, MA, USA). All the protein bands were exposed with Super ECL Detection Reagent (36,208, YEASON, China) on the Chemiluminescence imaging system (Cytiva, USA). The protein bands were analyzed by Image J software (NIH, USA). All the blots were cut prior to hybridisation with antibodies, so some full-length blots cannot be provided. However, all membranes are cut along the corresponding upper and lower markers according to the molecular weight of proteins to ensure the accuracy of the experiment. All the original images are displayed in the supplementary material.

### Cell proliferation assay

The cells were seeded in 96-well plates at a density of 1500 cells/well for culturing. In the next 5 days (one time one day), each well was added with CCK8 solution (cell counting kit-8, YEASON, China) and incubated at 37 °C for 2 h. Optical density (OD) was detected at 450 nm using a microplate reader.

### Edu assay

The cells were labeled with Edu and then collected for Apollo staining according to the instructions (Cell-Light EdU Apollo 643 In Vitro Imaging Kit, RiboBio, China). Flow cytometry was performed immediately after staining to detect Edu positive cells and predict cell proliferation.

### Cell cycle assay

The collected cells were washed with pre-cooled phosphate buffer saline (PBS) and then fixed in pre-cooled absolute ethyl alcohol through the night at 4 °C. Next day, after discarding the anhydrous ethanol, PBS was added into the cells and placed at room temperature for 15 min. The cells were then incubated with DNA staining solution (Cell Cycle Staining Kit, CCS012, MULTI SCIENCES, China) for 30 min in the dark. The cell cycle was analyzed by flow cytometry.

### Phospho-specific protein microarray analysis

HO8910 control cells and GSG2 knockdown cells were collected for protein extraction. Cell lysates (500 μg per array set) were applied to the Phospho-Antibody Array, using Human Phospho-Kinase Array Kit (ARY003C, R&D Systems, USA) for the detection of 37 site-specific phospho-antibody profiles. Finally, Pixel densities on developed X-ray film can be collected and analyzed using a transmission-mode scanner (Cytiva, USA) and image analysis software (Photoshop, Adobe, USA).

### Immunohistochemical Staining and evaluation

Tissue microarray containing 108 tumor tissues of ovarian cancer patients were obtained from the Department of Ovarian Surgery, Fudan University Shanghai Cancer Center. The study was endorsed by the Ethics Committee of Shanghai Cancer Center, Fudan University. Written informed consent was available from all patients. The final diagnosis of ovarian carcinoma was confirmed by histological analysis. GSG2 was detected with a rabbit polyclonal antibody at a dilution of 1:200(NBP1-26626, NOVUS, USA). Phospho-GSK-3α(Ser21) was detected with a rabbit polyclonal antibody at a dilution of 1:100(AF3336, Affinity, China). IHC staining and scoring criteria were described as our previous publications (Huang et al. 2021).

### Statistical analysis

Statistical analysis was performed using SPSS 24.0 software. The data were compared between two groups by an independent Students’ t-test. The data from CCK8 assays between groups were evaluated by Two-way ANOVA. Pearson Correlation Coefficient was used to analyze the correlation between the two quantifications. Differences were considered statistically significant when P < 0.05.

## Result

### Down-regulation of GSG2 inhibits cell proliferation and induces G2/M-phase arrest.

**Fig. 1:**
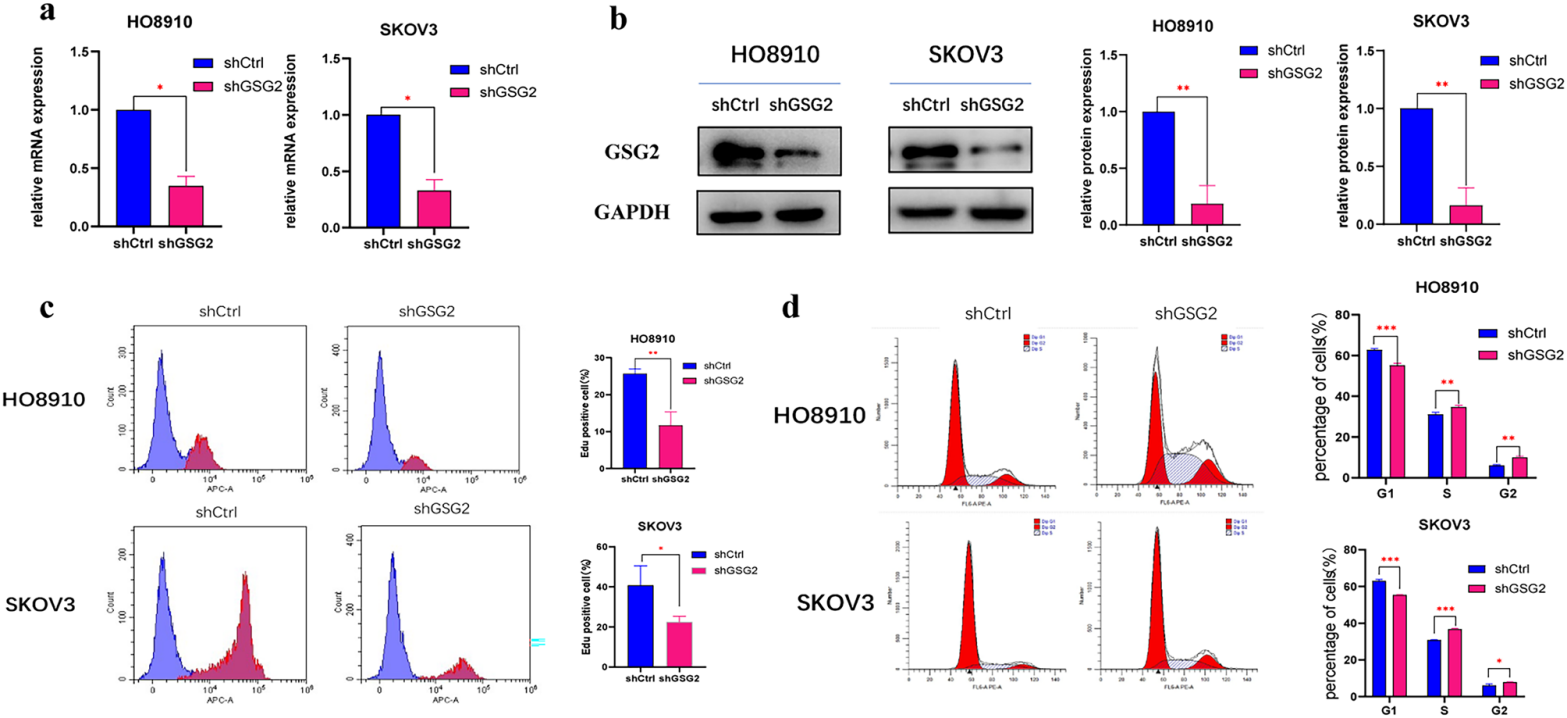
Down-regulation of GSG2 inhibits cell proliferation and induces G2/M-phase arrest. **a**, **b**. The knockdown efficiency of GSG2 in HO8910 and SKOV3 cells was determined by qRT-PCR and Western blotting. **c** Downregulation of GSG2 in HO8910 and SKOV3 cells inhibited cell proliferation. The percentage of Edu positive cells was measured by flow cytometry. **d** Knockdown of GSG2 in HO8910 and SKOV3 cells induced cell cycle arrest. The percentages of each cell cycle phase were presented at the bar graph. All experiments were carried out in triplicate. Data are shown as mean ± SD. *P < 0.05, **P < 0.01, ***P < 0.001

**Fig. 2:**
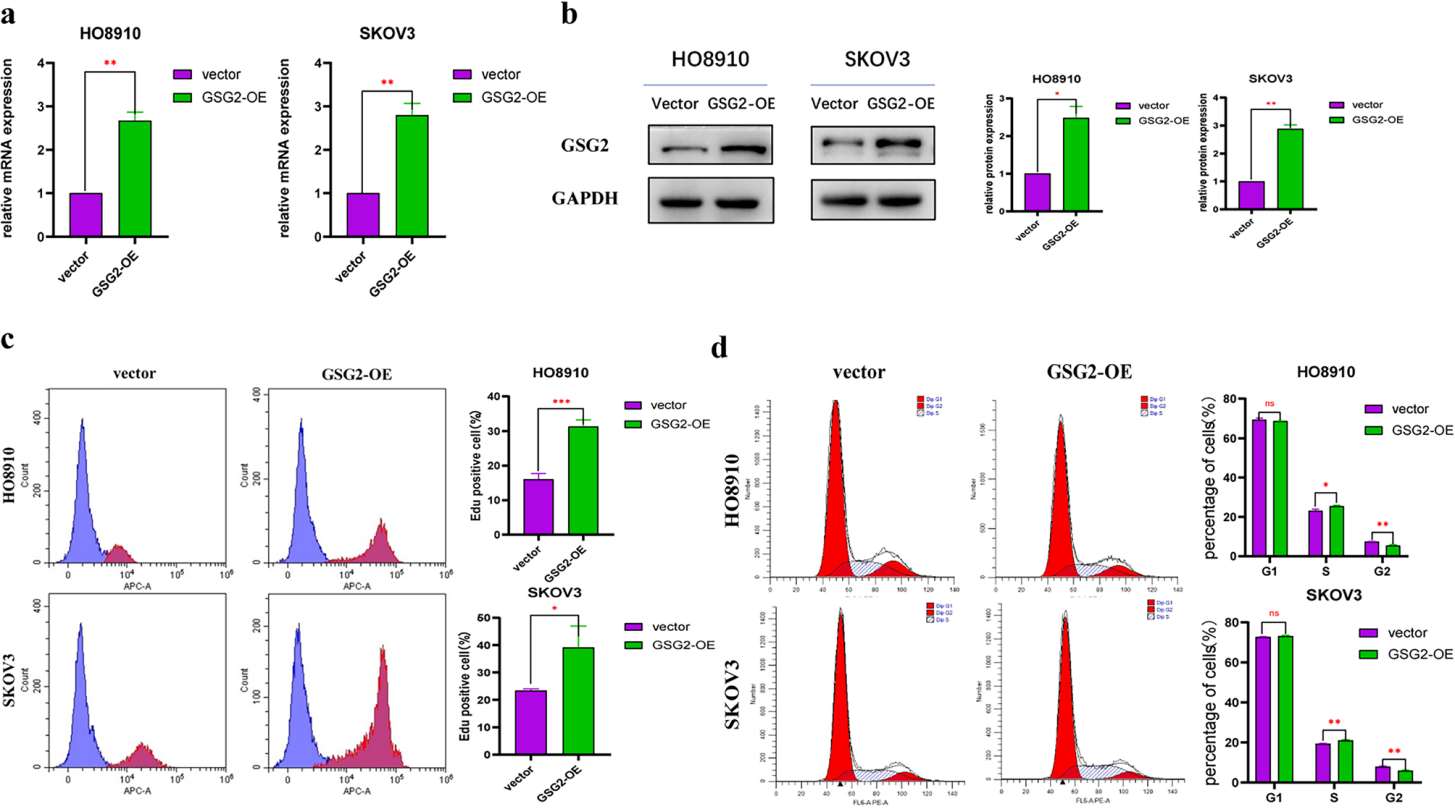
Overexpression of GSG2 promotes cell proliferation and regulates cell cycle. **a**, **b** The overexpression efficiency of GSG2 in HO8910 and SKOV3 cells was determined by qRT-PCR and Western blotting. **c** The percentage of Edu positive cells increased in GSG2-OE cells compared with control cells. **d** Overexpression of GSG2 in HO8910 and SKOV3 cells induced the decline of G2 phase cells. All experiments were carried out in triplicate. Data are shown as mean ± SD. *P < 0.05, **P < 0.01, ***P < 0.001

Previous studies have reported that GSG2 promotes the development and progression of ovarian cancer (Huang et al. 2021). In order to further explore the potential underlying mechanism of GSG2 promoting EOC proliferation, we down-regulated the expression of GSG2 in Human ovarian cancer cells HO8910 and SKOV3 with Lentivirus-mediated RNAi, and the knockdown efficiency was verified by western blotting and real-time PCR (Fig. 1a, b). By Edu assay, the percentage of Edu positive cells dropped after downregulation of GSG2 (Fig. 1c). The suppression of GSG2 in HO8910 and SKOV3 cells indeed inhibited cell proliferation. As an important protein involved in mitosis, GSG2 has been reported to accelerate cell cycle progression in cancer cells (Wang et al. 2020) and accelerated cell cycle was considered as a key drive for boosted proliferation in cancer. By flow cytometry, we observed the impact of GSG2 on cell cycle progression in EOC. Results demonstrated that HO8910 and SKOV3 cells were arrested in G2/M phase after knockdown of GSG2, meanwhile the percentage of cells in G1 phase decreased compared with control cells (Fig. 1d). GSG2 expressions were also overexpressed in HO8910 and SKOV3 cells to validate the regulatory role of GSG2 in cell cycle (Fig. 2a, b). Results showed that overexpression of GSG2 promoted cell proliferation and accelerated cell cycle, evidenced by that G2/M phase cells significantly decreased in GSG2-overexpressed(OE) cells when compared with control cells (Fig. 2c, d).

### Knockdown of GSG2 increases the expression of p27 and modulates G2/M phase proteins

**Fig. 3:**
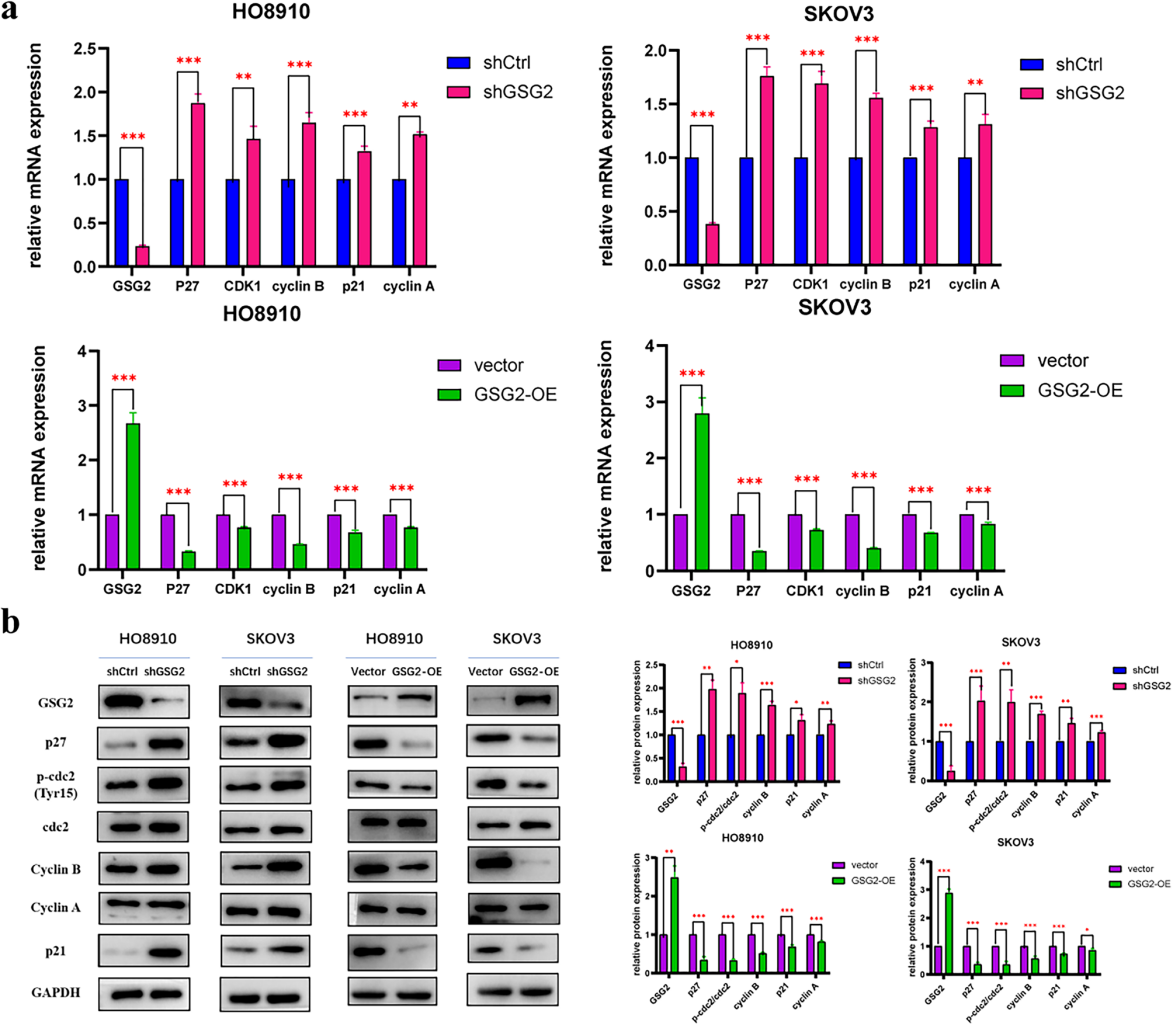
GSG2 regulates key cell cycle molecules of G2/M phase. **a** Cell cycle-related molecules were detected by qRT-PCR. After suppression of GSG2 in HO8910 and SKOV3 cells, the expression of p27, CDK1, cyclin B, p21 and cyclin A increased. In GSG2-OE cells, the expression of these molecules decreased. **b** Key proteins of G2/M phase were detected by western blotting. Downregulation of GSG2 in HO8910 and SKOV3 cells increase the phosphorylation level of cdc2 at Tyr15 and the expression of cyclin B. The expression of p27 notably increased while the expression of p21 and cyclin A slightly increased. The variation was reversed in GSG2-OE cells. Data are presented as the mean ± SD of three independent experiments. *P < 0.05, **P < 0.01, ***P < 0.001

As previously confirmed, GSG2 regulates the cell cycle progression of EOC cells so that we examined the expression of several cell cycle-related molecules, especially those key molecules involved in regulating G2/M phase. As shown in Fig. 3a, b, the expression of p27 remarkably increased in GSG2-knockdown cells on the both level of mRNA and protein to the most extent, and the variation was reversed in GSG2-OE cells. As key molecules of G2/M phase, the expression of CDK1 and cyclin B similarly increased and the phosphorylation level of cdc2 was elevated at Tyr15 after suppression of GSG2. Contrarily, opposite alterations of these proteins were observed in GSG2-OE cells. In addition, the expression of p21 and cyclin A was only slightly increased in GSG2-knockdown cells compared with control cells and the trend was inverse in GSG2-OE cells. Collectively, these results implied p27 as the potential downstream target of GSG2 in modulating cell cycle in EOC cells.

### GSG2 phosphorylates GSK3α at Ser21 in epithelial ovarian cancer

**Fig. 4:**
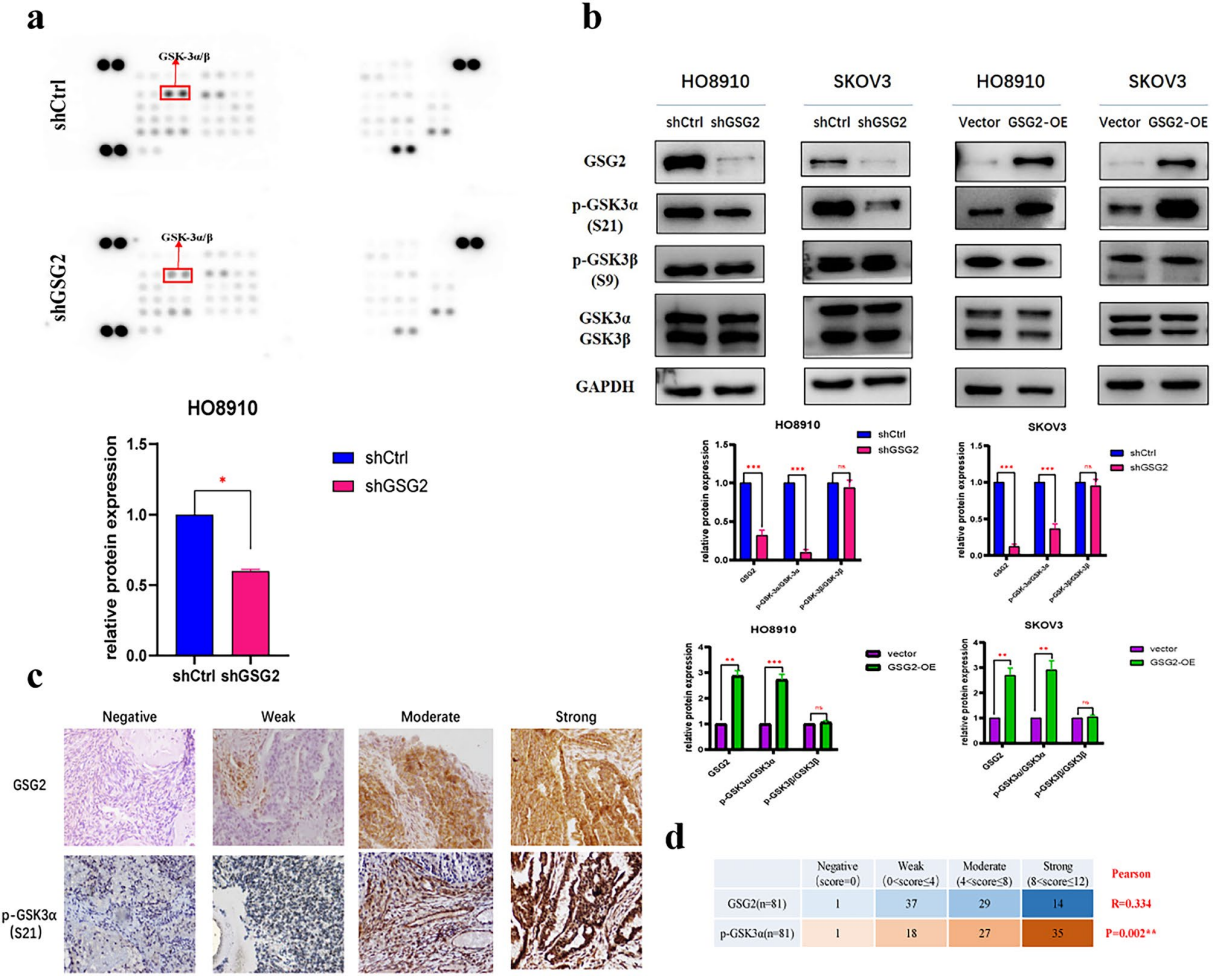
GSG2 phosphorylates GSK3α at Ser21 in epithelial ovarian cancer. **a** Expression of 37 proliferation-related phosphorylated kinases was detected using Human Phospho-kinase Array Kit, and the phosphorylation level of GSK3α/β was significantly reduced after downregulation of GSG2. **b** Knockdown of GSG2 inhibited the expression of p-GSK3α(S21) but not p-GSK3β(S9). **c**, **d** The Correlation Analysis between GSG2 and p-GSK3α(S21) was performed based on epithelial ovarian cancer tissue staining scores and Pearson correlation coefficient is 0.334(**P < 0.01)

Next, we used Human Phospho-kinase Array Kit to explore the potential downstream signaling pathway of GSG2. It turned out that the phosphorylation level of GSK3α/βreduced mostly in GSG2-knockdown cells among all 37 phosphorylated kinases (Fig. 4a). We detected the expression of the two proteins respectively by western blotting to determine that it was the expression of p-GSK3α(S21) not p-GSK3β(S9) that decreased in GSG2-knockdown cells and the phosphorylation level of GSK3α at S21 also increased in GSG2-OE cells (Fig. 4b). On account of the off-target effect, small interfering RNA was used to verify this finding, and the experimental results agree with the above result (Supplementary Fig. 1a). Then, 88 epithelial ovarian cancer tissues were stained with GSG2 and p- GSK3α(S21) antibodies respectively and correlation analysis was performed based on staining scores (Fig. 4c). The result showed that GSG2 and p- GSK3α(S21) are positively correlated and Pearson correlation coefficient is 0.334(P < 0.01) (Fig. 4d).

### GSG2 mediates GSK3α to regulate cell cycle in epithelial ovarian cancer.

**Fig. 5:**
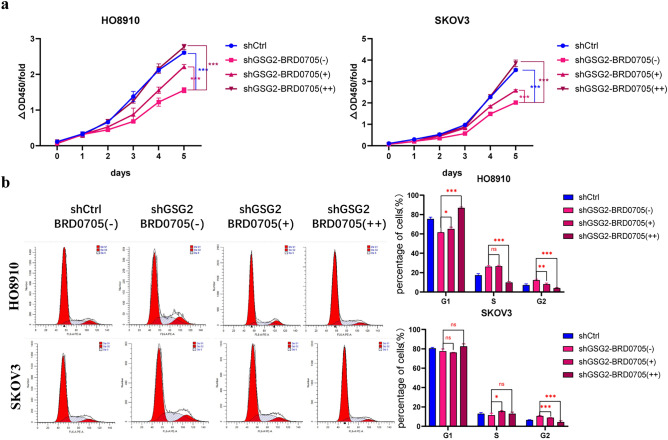
GSG2 mediates GSK3α to regulate cell proliferation and cell cycle. **a** The inhibition of proliferation of GSG2-knockdown cells was relieved after using GSK3α inhibitor **b** G2/M phase arrest caused by the downregulation of GSG2 is relieved after using BRD0705. The percentages of each cell cycle phase were presented at the bar graph. BRD0705(-),0 μM; BRD0705( +),10 μM; BRD0705(+ +),20 μM. Data are presented as the mean ± SD of three independent experiments. *P < 0.05, **P < 0.01, ***P < 0.001

GSK3α is widely known as a promoting factor of apoptosis and the phosphorylation at Ser21 inhibits its activity. After down-regulation of GSG2, the phosphorylation level of GSK3α^Ser21^ declined, activating the pro-apoptotic function of GSK3α, hence we used BRD0705, a potent, paralog selective GSK3α inhibitor to inhibit the activity of GSK3α and observe cell proliferation. Based on previous studies (Wagner 2018), the optimal concentration of BRD0705 was selected as 10 μM and 20 μM. The inhibition of proliferation in GSG2-knockdown cells was relieved after using GSK3α inhibitor (Fig. 5a). In the same way, the percentage of G2/M phase cells declined on account of the use of GSK3α inhibitor. As a result, G2/M phase arrest due to the downregulation of GSG2 was restored (Fig. 5b). Overall these data validated the critical role of GSK3α in GSG2-mediated cell cycle and proliferation of EOC cells.

### GSK3α regulates the expression of p27 in GSG2-knockdown ovarian cancer cells

**Fig. 6:**
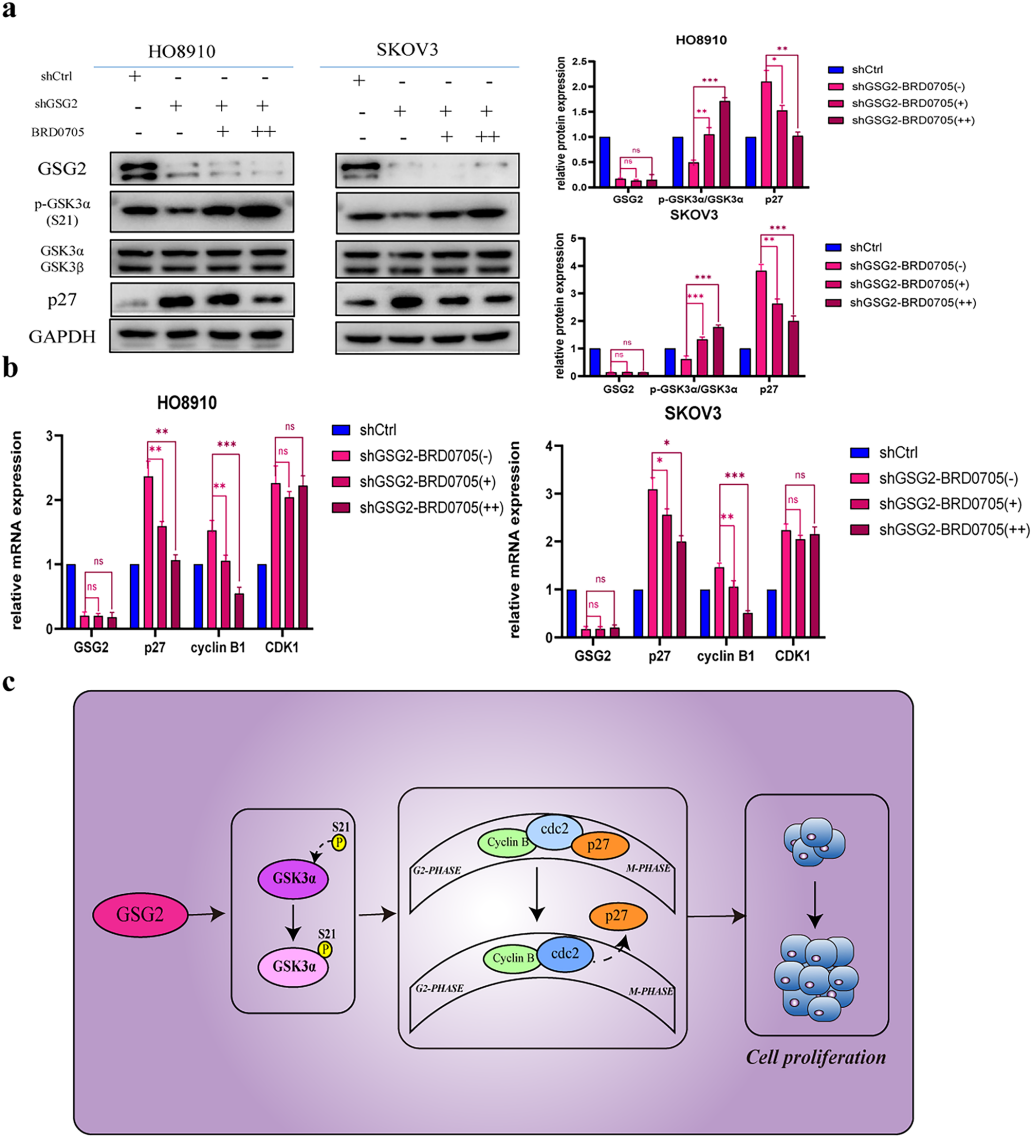
GSK3α regulates the expression of p27 in epithelial ovarian cancer cells. **a** BRD0705-treated GSG2 knockdown cells enhanced the phosphorylation level of GSK3α at Ser21 but inhibited the level of p27 compared with control cells. **b** BRD0705-treated GSG2 knockdown cells increased the expression of cyclin B while decreased the expression of p27 on the level of mRNA in compare with control cells. **c** Schematic model of GSG2 and GSK3α regulate p27 during G2/M phase. In epithelial ovarian cancer, GSG2 reduces the expression of p27 in the G2 cell cycle by phosphorylating GSK3α, and fails to bind to cdc2 to inhibit its activation, resulting in cell cycle disorder and massive cell proliferation. Data are presented as the mean ± SD of three independent experiments. *P < 0.05, **P < 0.01, ***P < 0.001

In GSG2-knockdown cells, the phosphorylation level of GSK3α decreased while the expression of p27 notably increased. Therefore, we wondered if GSK3α would regulate the expression of p27. GSK3α inhibitor was added to GSG2-knockdown cells and the expression of P27 decreased at the same time as the phosphorylation level of GSK increased (Fig. 6a). Furthermore, the mRNA expression of cyclin B declined (Fig. 6b) while the expression of CDK1 was unchanged. We speculate that GSK3α regulates the expression of p27 during G2/M phase to modulate cell cycle and proliferation. Combined with aforementioned regulation of GSG2 on GSK3α, we predicted the mechanism of GSG2 promoting the development of epithelial ovarian cancer. (Fig. 6c).

## Discussion

GSG2 is an atypical serine/threonine-protein kinase that specifically phosphorylates histone H3 at Thr3 during mitosis. Depletion of GSG2 in mitotically active cells results in low phosphorylation level of histone H3 which promoting the accumulation of cells during prometaphase (Dai et al. 2005), preventing normal chromosome alignment and activating the spindle assembly checkpoint. Recently, GSG2 have been reported to act as a cancer-promoting factor in various human tumors (Huertas et al. 2012), such as prostate cancer (Yu et al. 2020), gallbladder cancer (Zhu et al. 2020), and pancreatic cancer (Han et al. 2019).

Previous study has showed that GSG2 promotes the development of ovarian cancer (Huang et al. 2021), however it has not elaborated on the mechanism. Therefore, we focus on the mechanism of GSG2 promoting the development of EOC.

We constructed GSG2-knockdown cells with Human ovarian cancer cells HO8910 and SKOV3 by short hairpin RNA interference and the suppression of GSG2 inhibited cell proliferation. It is acknowledged that GSG2 plays a critical role in mitosis, favoring chromosome cohesion, metaphase alignment and progression (Xie et al. 2015), thus we laid emphasis on the role of GSG2 in cell cycle progression. The expression level of GSG2 increased significantly in G2 phase, and the cells continued to enter the mitotic phase. Downregulation of GSG2 induced G2/M phase arrest and the expression of p27 mostly increased. In addition, there was a notable variation on the expression of key proteins of G2/M phase in GSG2-knockdown cells such as cyclin B and phoshpo-cdc2^Tyr15^, therefore we concluded that GSG2 could regulate cell cycle in epithelial ovarian cancer. In order to further determine the function of GSG2 in EOC, we overexpressed GSG2 by lentivirus transfection in HO8910 and SKOV3 cells and the results supported this conclusion.

In order to explore the potential downstream signal pathway of GSG2, we used phospho-kinase array and discovered the significant decreased phosphorylation of GSK-3α^Ser21^ after downregulation of GSG2 while there is no change in the phosphorylation level of GSK3β. Glycogen synthase kinase 3(GSK3) is a serine/threonine group of protein kinases with two known members, GSK3α and GSK3β, and identified to be crucial in promoting apoptosis during cellular process. Unlike most kinases, GSK3 is active under basal conditions and requires extracellular signaling for its inactivation. The phosphorylated residues function as pseudo substrates blocking the substrate binding site and GSK3α activity is inhibited by phosphorylation at S21 (Frame et al. 2001). Although it is verified that GSK3β positively regulates the proliferation of human ovarian cancer cells (Cao et al. 2006), the association between GSK3α and ovarian cancer has not been demonstrated. Our study proved that GSG2 and p- GSK3α(S21) are positively correlated and GSG2 regulates the phosphorylation of GSK3α^Ser21^ in epithelial ovarian cancer.

It is recognized that GSK-3 has a vital effect on the cell cycle and is capable of phosphorylating a number of proteins involved in cell cycle progression (McCubrey et al. 2014; Mishra et al. 2015). Our study showed that GSG2 obviously regulated the expression of p27, a Cyclin-dependent kinase (CDK) inhibitor belonging to the CIP/Kip (Cyclin Inhibitory Protein/Kinase inhibitory protein) family. It participates in the control of G2 advancement and M phase/cytokinesis completion and forms inactive ternary complexes with cyclin B/CDK1, contributing to G2/M phase delay (Bencivenga et al. 2021). Thus, we speculated whether GSK3α could affect p27 expression to regulate cell cycle in EOC. We used GSK3α inhibitor to inhibit the activity of GSK3α that increased by downregulation of GSG2. It turned out that the inhibition of cell proliferation and cell cycle delay reversed after applying GSK3α inhibitor to GSG2-knockdown cells and the increased expression of p27 visibly reduced caused by GSG2 knockdown. Meanwhile, the expression of cyclin B jointly reduced, indicating that the decrease of p27 inhibited the inactivation of cyclin B/CDK1. Therefore, we conjecture that GSG2 mediates GSK3α to regulate p27 expression in epithelial ovarian cancer. However, we also noticed that even with high doses of GSK3α inhibitors, p27 expression did not return to the level of shCtrl cell expression, suggesting that p27 is not only regulated by GSK3α, but also by other proteins acting on p27 to regulate the cell cycle. This is where we need to explore further.

In conclusion, our study demonstrates that knockdown of GSG2 has an important influence on the phosphorylation of GSK3α^Ser21^ and results in the distinctly increased expression of p27 that leads to cell cycle arrest and inhibits cell proliferation in EOC. Thus, we regard GSG2 as a promising therapeutic target in epithelial ovarian cancer. However, we have not got an overall understanding of mechanism of the phosphorylation of GSK3α^Ser21^ decreasing in GSG2-knockdown cells. We surmise that GSG2 regulates GSK3α by another protein, which is able to directly phosphorylate GSK3α because there is no interaction between GSG2 and GSK3α. In addition, the regulation mechanism of GSK3α on p27 is not clear, and we have not known whether GSK3α directly regulates the expression of p27. Based on these speculation and query, we will perform more precise and detailed research.

### Supplementary Information

Below is the link to the electronic supplementary material.

Supplementary file1 (ZIP 11,586 kb)

## Data Availability

The data that support the findings of this study are available on request from the corresponding author. The data are not publicly available due to privacy or ethical restrictions.

## References

[CR1] Armstrong DK, Alvarez RD, Backes FJ, Bakkum-Gamez JN, Barroilhet L, Behbakht K (2022). Nccn guidelines(r) insights: ovarian cancer, version 3.2022. J Natl Compr Canc Netw.

[CR2] Bencivenga D, Stampone E, Roberti D, Della RF, Borriello A (2021). p27(Kip1), an intrinsically unstructured protein with scaffold properties. Cells-Basel.

[CR3] Cao Q, Lu X, Feng YJ (2006). Glycogen synthase kinase-3beta positively regulates the proliferation of human ovarian cancer cells. Cell Res.

[CR4] Chiang YC, Lin PH, Cheng WF (2021). Homologous recombination deficiency assays in epithelial ovarian cancer: current status and future direction. Front Oncol.

[CR5] Dai J, Sultan S, Taylor SS, Higgins JM (2005). The kinase Haspin is required for mitotic histone H3 Thr 3 phosphorylation and normal metaphase chromosome alignment. Genes Dev.

[CR6] Dai J, Sullivan BA, Higgins JM (2006). Regulation of mitotic chromosome cohesion by Haspin and Aurora B. Dev Cell.

[CR7] Dai J, Kateneva AV, Higgins JM (2009). Studies of haspin-depleted cells reveal that spindle-pole integrity in mitosis requires chromosome cohesion. J Cell Sci.

[CR8] Frame S, Cohen P, Biondi RM (2001). A common phosphate binding site explains the unique substrate specificity of GSK3 and its inactivation by phosphorylation. Mol Cell.

[CR9] Han X, Kuang T, Ren Y, Lu Z, Liao Q, Chen W (2019). Haspin knockdown can inhibit progression and development of pancreatic cancer in vitro and vivo. Exp Cell Res.

[CR10] Higgins JM (2001). The Haspin gene: location in an intron of the integrin alphaE gene, associated transcription of an integrin alphaE-derived RNA and expression in diploid as well as haploid cells. Gene.

[CR11] Huang Y, Liu Y, Zhu K, Ma X, Lu R, Zhang M (2021). GSG2 promotes development and predicts poor prognosis of ovarian cancer. Cancer Manag Res.

[CR12] Huertas D, Soler M, Moreto J, Villanueva A, Martinez A, Vidal A, Charlton M, Moffat D, Patel S, McDermott J, Owen J, Brotherton D, Krige D, Cuthill S, Esteller M (2012). Antitumor activity of a small-molecule inhibitor of the histone kinase Haspin. Oncogene.

[CR13] Kestav K, Uri A, Lavogina D (2017). Structure, roles and inhibitors of a mitotic protein kinase Haspin. Curr Med Chem.

[CR14] McCubrey JA, Steelman LS, Bertrand FE, Davis NM, Sokolosky M, Abrams SL, Montalto G, D’Assoro AB, Libra M, Nicoletti F, Maestro R, Basecke J, Rakus D, Gizak A, Demidenko ZN, Cocco L, Martelli AM, Cervello M (2014). GSK-3 as potential target for therapeutic intervention in cancer. Oncotarget.

[CR15] Mishra R, Nagini S, Rana A (2015). Expression and inactivation of glycogen synthase kinase 3 alpha/ beta and their association with the expression of cyclin D1 and p53 in oral squamous cell carcinoma progression. Mol Cancer.

[CR16] Siegel RL, Miller KD, Wagle NS, Jemal A (2023). Cancer statistics, 2023. CA Cancer J Clin.

[CR17] Tanaka H, Yoshimura Y, Nishina Y, Nozaki M, Nojima H, Nishimune Y (1994). Isolation and characterization of cDNA clones specifically expressed in testicular germ cells. FEBS LETT.

[CR18] Tanaka H, Yoshimura Y, Nozaki M, Yomogida K, Tsuchida J, Tosaka Y, Habu T, Nakanishi T, Okada M, Nojima H, Nishimune Y (1999). Identification and characterization of a haploid germ cell-specific nuclear protein kinase (Haspin) in spermatid nuclei and its effects on somatic cells. J Biol Chem.

[CR19] Torre LA, Trabert B, DeSantis CE, Miller KD, Samimi G, Runowicz CD, Gaudet MM, Jemal A, Siegel RL (2018). Ovarian cancer statistics, 2018. CA Cancer J Clin.

[CR20] Wagner FF, Benajiba L, Campbell AJ, Weiwer M, Sacher JR, Gale JP, Ross L, Puissant A, Alexe G, Conway A, Back M, Pikman Y, Galinsky I, DeAngelo DJ, Stone RM, Kaya T, Shi X, Robers MB, Machleidt T, Wilkinson J, Hermine O, Kung A, Stein AJ, Lakshminarasimhan D, Hemann MT, Scolnick E, Zhang YL, Pan JQ, Stegmaier K, Holson EB (2018). Exploiting an Asp-Glu “switch” in glycogen synthase kinase 3 to design paralog-selective inhibitors for use in acute myeloid leukemia. Sci Transl Med.

[CR21] Wang F, Dai J, Daum JR, Niedzialkowska E, Banerjee B, Stukenberg PT, Gorbsky GJ, Higgins JM (2010). Histone H3 Thr-3 phosphorylation by Haspin positions Aurora B at centromeres in mitosis. Science.

[CR22] Wang P, Hua X, Bryner YH, Liu S, Gitter CB, Dai J (2020). Haspin inhibition delays cell cycle progression through interphase in cancer cells. J Cell Physiol.

[CR23] Xie J, Wooten M, Tran V, Chen BC, Pozmanter C, Simbolon C, Betzig E, Chen X (2015). Histone H3 threonine phosphorylation regulates asymmetric histone inheritance in the drosophila male germline. Cell.

[CR24] Yu F, Lin Y, Xu X, Liu W, Tang D, Zhou X, Wang G, Zheng Y, Xie A (2020). Knockdown of GSG2 inhibits prostate cancer progression in-vitro and in-vivo. INT J ONCOL.

[CR25] Zhang H, Zhong A, Sun J, Chen M, Xie S, Zheng H, Wang Y, Yu Y, Guo L, Lu R (2017). COPS5 inhibition arrests the proliferation and growth of serous ovarian cancer cells via the elevation of p27 level. Biochem Biophys Res Commun.

[CR26] Zhu D, Gu X, Lin Z, Yu D, Wang J, Li L (2020). HASPIN is involved in the progression of gallbladder carcinoma. Exp Cell Res.

