# Supplementary material for: Germ cell-specific gene 2 accelerates cell cycle in epithelial ovarian cancer by inhibiting GSK3α-p27 cascade
Source: J Mol Histol. 2024 Apr 13;55(3):241–51. doi: 10.1007/s10735-024-10185-6 (PMC11102877; doi:10.1007/s10735-024-10185-6)
Supplement: Supplementary file 1 — Supplementary file1 (ZIP 11,586 kb) [file 10735_2024_10185_MOESM1_ESM.zip › 10735_2024_10185_MOESM1_ESM/Supplementary Material/HO8910 STR Profiling Report.pdf]

Cell Line Authentication Service

STR Profiling Report

Sample From: Fudan University Shanghai Cancer Center

Sample Type: Cell Line

Testing Method: STR Genotyping

Report Time: March 24, 2022

Add: Room 502, NO.1015 Longteng Rd , Songjiang District, Shanghai/ Tel:+86-18521538068
Homepage: http://www.biowing.com.cn/ E-mail: market@biowing.com.cn

COMPANY STATEMENT

1. THIS REPORT IS ONLY RESPONSIBLE FOR THE SAMPLES ANALYZED.
2. THE TESTING RESULTS AND THE ORGANIZATION NAME WILL NOT BE USED FOR ADVERTISEMENT, COMMERCIAL EXHIBITIONS, COMMERCIAL PERFORMANCE AND OTHER COMMERCIAL ACTIVITIES.
3. OBJECTIONS SHOULD BE RAISED WITHIN FIFTEEN DAYS AFTER THE RECEIPT OF THIS REPORT.
4. THE PAPER REPORT WITH CONTENT ALTERING, ADDING OR WITHOUT THE STAMPED SEAL OF THE COMPANY ARE INVALID.

Testing Company: Shanghai Biowing Applied Biotechnology Co. Ltd

Address: Room 502, NO.1015 Longteng Rd , Songjiang District, Shanghai

Tel: +86-18521538068

Contact: Wenyao Zhang

E-mail: market@biowing.com.cn

Cell Line Authentication – STR Profiling Report

Sample code

Table 1. Sample Code

Customer's code	Company Code
HO-8910	20220323-02

Sample Number :1

Sample Type: Cell line

Testing Type: STR

Testing Method:

DNA was extracted by a commercial kit from CORNING (AP-EMN-BL-GDNA-250G). The twenty STRs including Amelogenin locus were amplified by six multiplex PCR and separated on ABI 3730XL Genetic Analyzer. The signals were then analyzed by the software GeneMapper.

Data Interpretation:

Cell lines were authenticated using Short Tandem Repeat (STR) analysis asdescribed in 2012 in ANSI Standard (ASN-0002) by the ATCC Standards Development Organization (SDO) and in Capes-Davis et al., Match criteria for human cell line authentication: Where do we draw the line? Int J Cancer.2013;132(11):2510-9.

Add: Room 502, NO.1015 Longteng Rd , Songjiang District, Shanghai/ Tel:+86-18521538068
Homepage: http://www.biowing.com.cn/ E-mail: market@biowing.com.cn

Test Results

1. STR profile

Table 2. STR and Amelogenin Genotyping Results of Cell line.

Loci	Sample information			Cell Bank information		
	Sample name：HO-8910			Cell line name：HO-8910		
	**Allele1**	**Allele2**	**Allele3**	**Allele1**	**Allele2**	**Allele3**
D5S818	11	12		11	12	
D13S317	13.3	13.3		13.3	13.3	
D7S820	8	12		8	12	
D16S539	9	10		9	10	
VWA	16	18		16	18	
TH01	7	7		7	7	
AMEL	X	X		X	X	
TPOX	8	12		8	12	
CSF1PO	9	10		9	10	
D12S391	20	25				
FGA	18	21				
D2S1338	17	17				
D21S11	27	28				
D18S51	16	17				
D8S1179	12	13				
D3S1358	15	18				
D6S1043	18	18				
PENTAE	7	17				
D19S433	13	14				
PENTAD	8	15				
D1S1656	12	15				

Add: Room 502, NO.1015 Longteng Rd , Songjiang District, Shanghai/ Tel:+86-18521538068
Homepage: http://www.biowing.com.cn/ E-mail: market@biowing.com.cn

2. database annotation

Figure 1. STR matching analysis

Name	N° Markers	Score	Amel	CSF1PO	D2S1338	D3S1358	D5S818	D6S1043	D7S820	D8S1179	D12S391	D13S317	D16S539	D18S51	D19S433	D21S11	FGA	Penta D	Penta E	TH01	TPOX	vWA
Query	NA	NA	X	9,10	17	15,18	11,12	18	8,12	12,13	20,25	13.3	9,10	16,17	13,14	27,28	18,21	8,15	7,17	7	8,12	16,18
HO-8910	19	100.00%	X	9,10	17	15,18	11,12	18	8,12	12,13	20,25	13.3	9,10	16,17	13,14	27,28	18,21	8,15	7,17	7	8,12	16,18

Note: The STR online match analysis of the test cell against EXPASY database, showing cell number (Cell No. CVCL_6868) and cell name.

3. Authentication

☐ The submitted sample profile is human, but not a match for any profile in the DSMZ STR database.

☑ The submitted profile is exact match for the following human cell line(s) in the EXPASY STR database (8 core loci plus Amelogenin): **HO-8910**.

☐ The submitted profile is similar to the following DSMZ human cell line: /.

- **Note:** A cell line can be considered to be authenticated when 80% (exact match) of the alleles in its STR profile match profiles from tissue or other cell line samples from that donor or from database. Cell lines with between a 55% to 80% (similar) match require further profiling for investigation of relatedness.

Figure 2. STR profiles of sample cell line

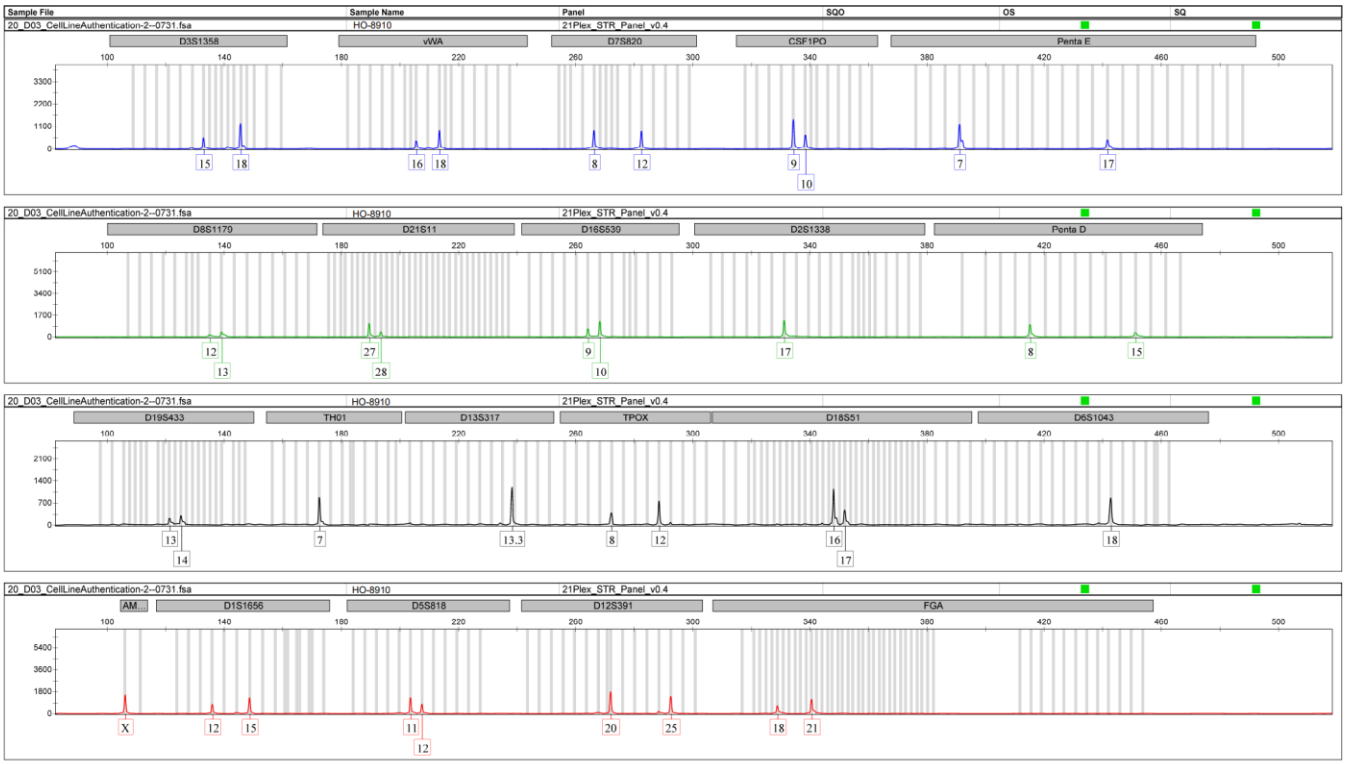

Add: Room 502, NO.1015 Longteng Rd , Songjiang District, Shanghai/ Tel:+86-18521538068
Homepage: http://www.biowing.com.cn/ E-mail: market@biowing.com.cn

Appendix

1. Genotyping Strategy and Site Distribution

Table S1. Experimental Strategy and Sites

	Strategy 1	Strategy 2	Strategy 3	Strategy 4
1	D3S1358	D8S1179	D19S433	AMEL
2	VWA	D21S11	TH01	D1S1656
3	D7S820	D16S539	D13S317	D5S818
4	CSF1PO	D2S1338	TPOX	D12S391
5	PENTAE	PENTAD	D18S51	FGA
6			D6S1043	

The allele match algorithm compares the 8 core loci plus amelogenin only, even though alleles from all lociwill be reported when available.

2. DSMZ tools was used to carry on the cell line comparison, which contains 2455 cell lines STR data from ATCC, DSMZ, JCRB ,ECACC， GNE and RIKEN databases. If the cell is not included in the above cell library, users need to compared with other databases.

Technician: Jianan Zhang

Checked by: Ning Qian

Issued by: Yang Bai

Issue date: March 24, 2022

Add: Room 502, NO.1015 Longteng Rd , Songjiang District, Shanghai/ Tel:+86-18521538068
Homepage: http://www.biowing.com.cn/ E-mail: market@biowing.com.cn
