# Supplementary figures and images for: Germ cell-specific gene 2 accelerates cell cycle in epithelial ovarian cancer by inhibiting GSK3α-p27 cascade (part 2 of 2)
Source: J Mol Histol. 2024 Apr 13;55(3):241–51. doi: 10.1007/s10735-024-10185-6 (PMC11102877; doi:10.1007/s10735-024-10185-6)

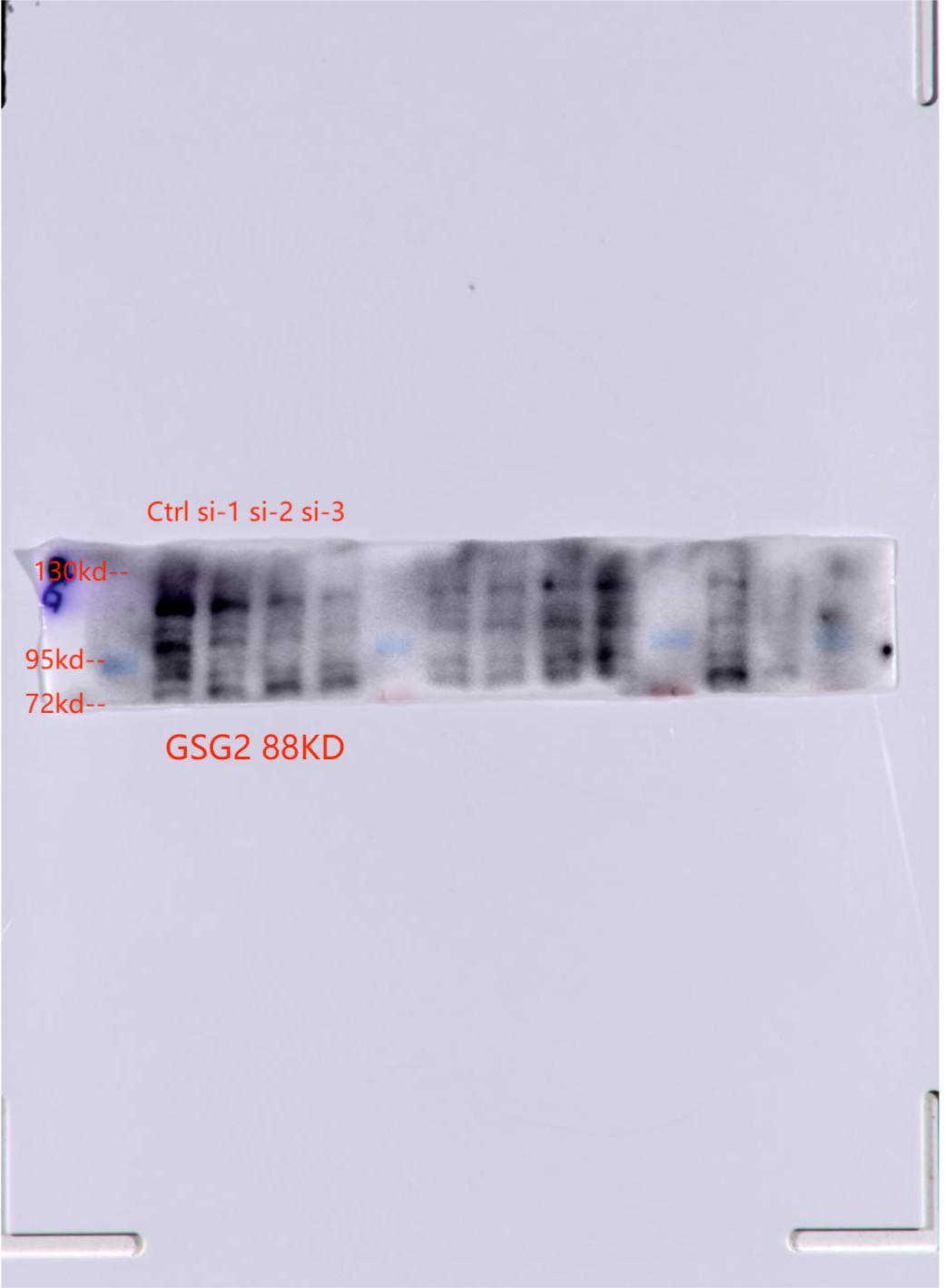

Supplement: Supplementary file 1 — Supplementary file1 (ZIP 11,586 kb) [file 10735_2024_10185_MOESM1_ESM.zip › 10735_2024_10185_MOESM1_ESM/Supplementary Material/Supplementary fig1A-SKOV3 GSG2.jpg]

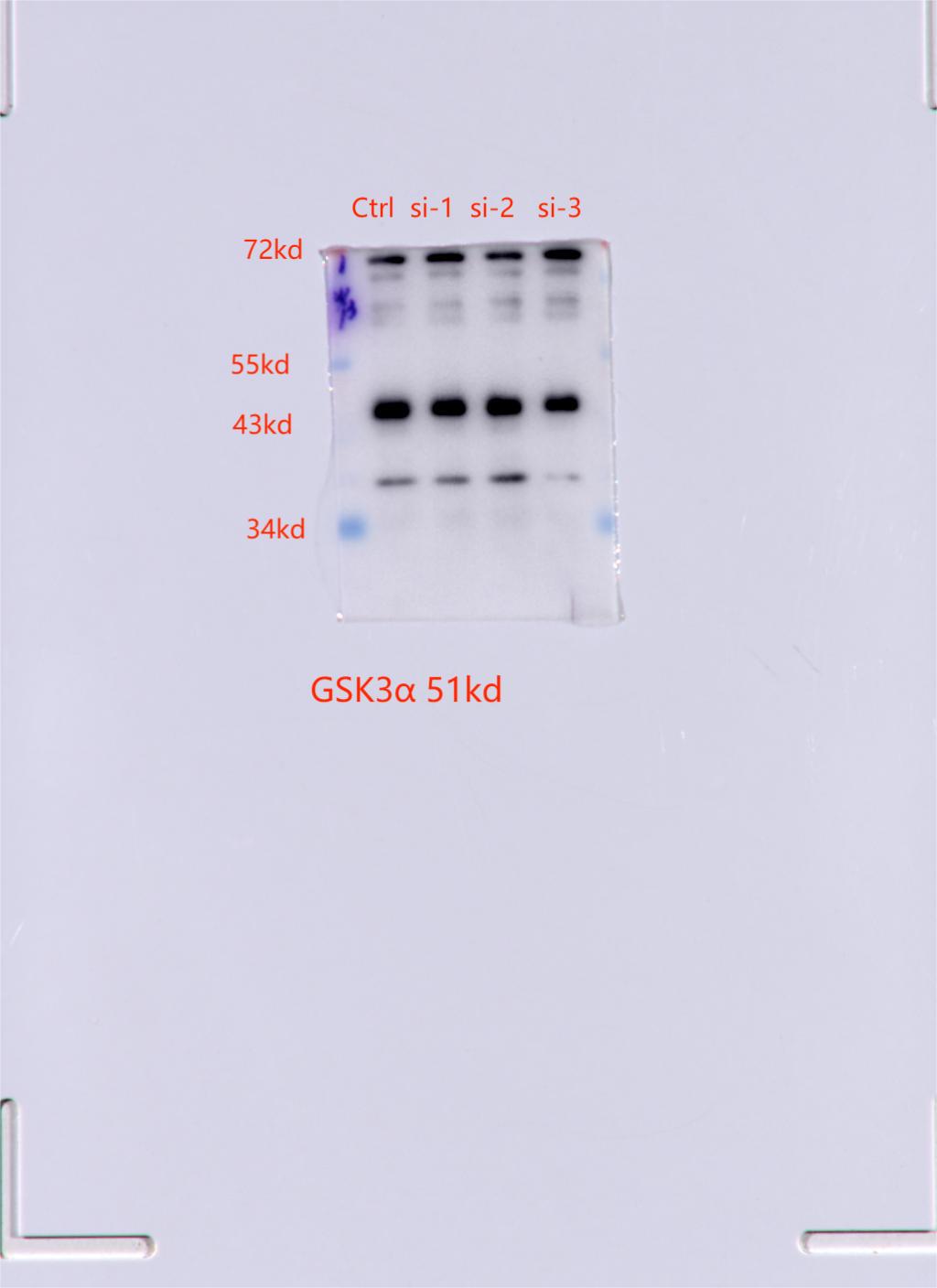

Supplement: Supplementary file 1 — Supplementary file1 (ZIP 11,586 kb) [file 10735_2024_10185_MOESM1_ESM.zip › 10735_2024_10185_MOESM1_ESM/Supplementary Material/Supplementary fig1A-SKOV3 GSKA.jpg]

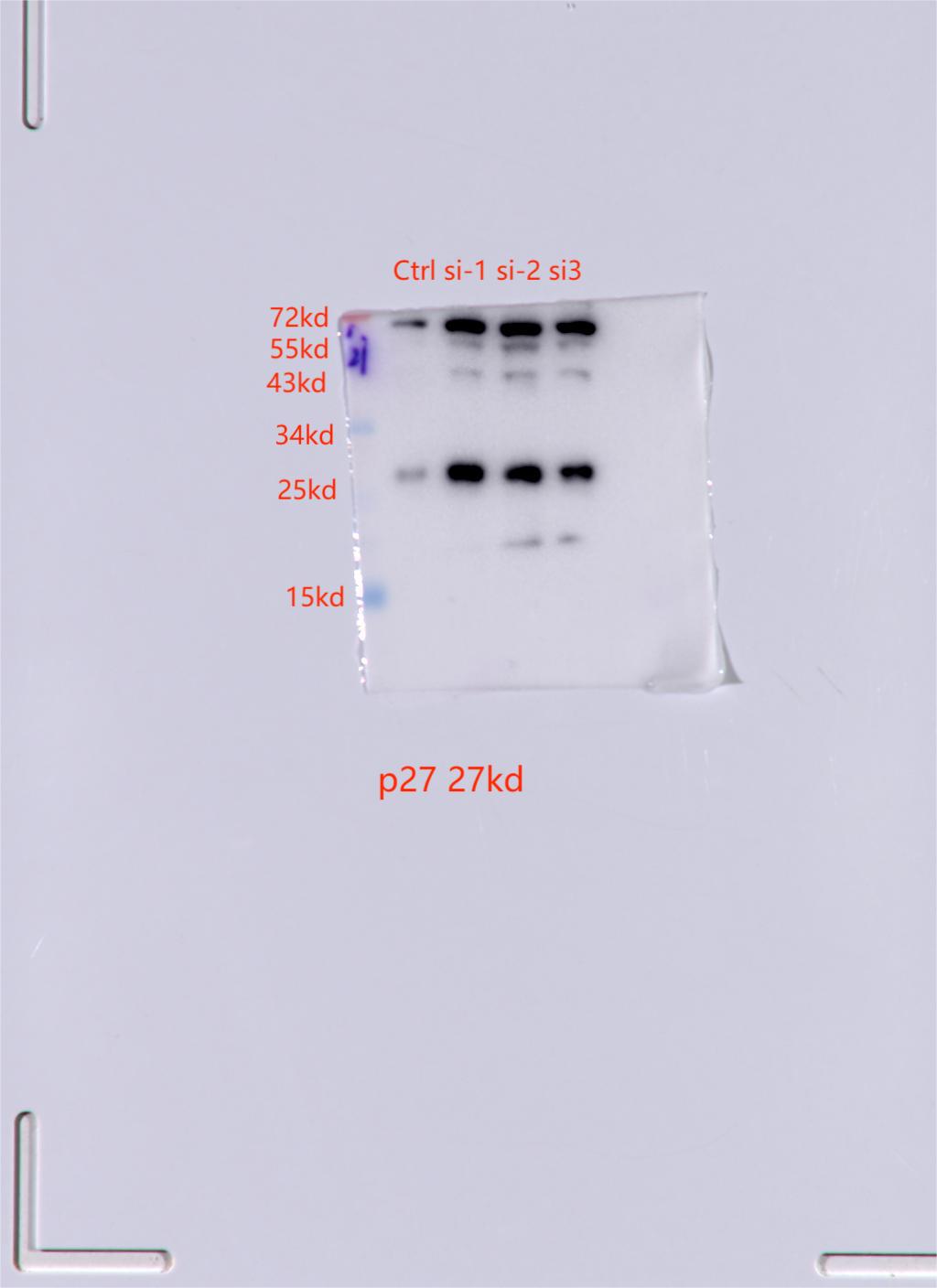

Supplement: Supplementary file 1 — Supplementary file1 (ZIP 11,586 kb) [file 10735_2024_10185_MOESM1_ESM.zip › 10735_2024_10185_MOESM1_ESM/Supplementary Material/Supplementary fig1A-SKOV3 p27.jpg]

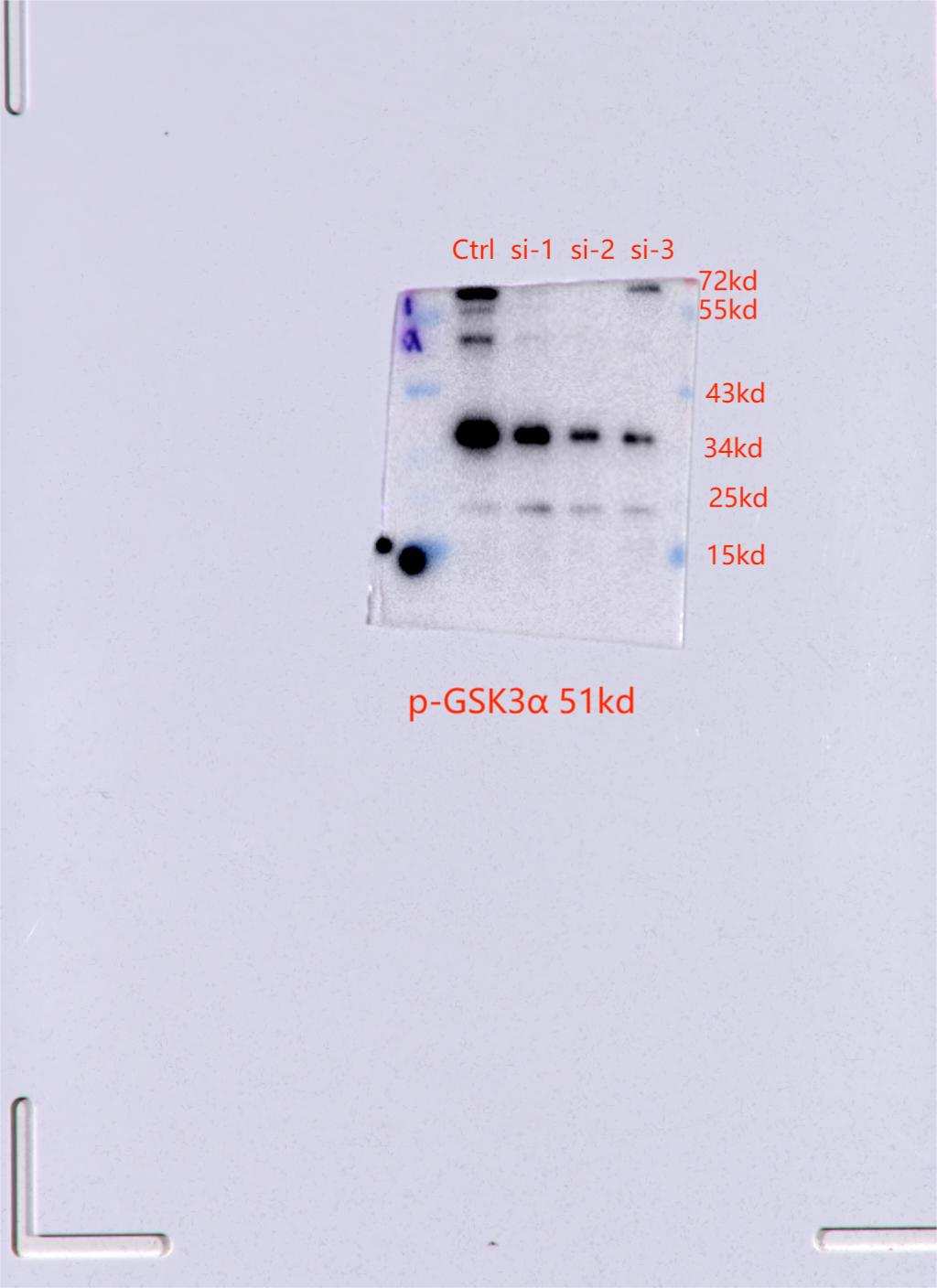

Supplement: Supplementary file 1 — Supplementary file1 (ZIP 11,586 kb) [file 10735_2024_10185_MOESM1_ESM.zip › 10735_2024_10185_MOESM1_ESM/Supplementary Material/Supplementary fig1A-SKOV3 pGSK3a .jpg]

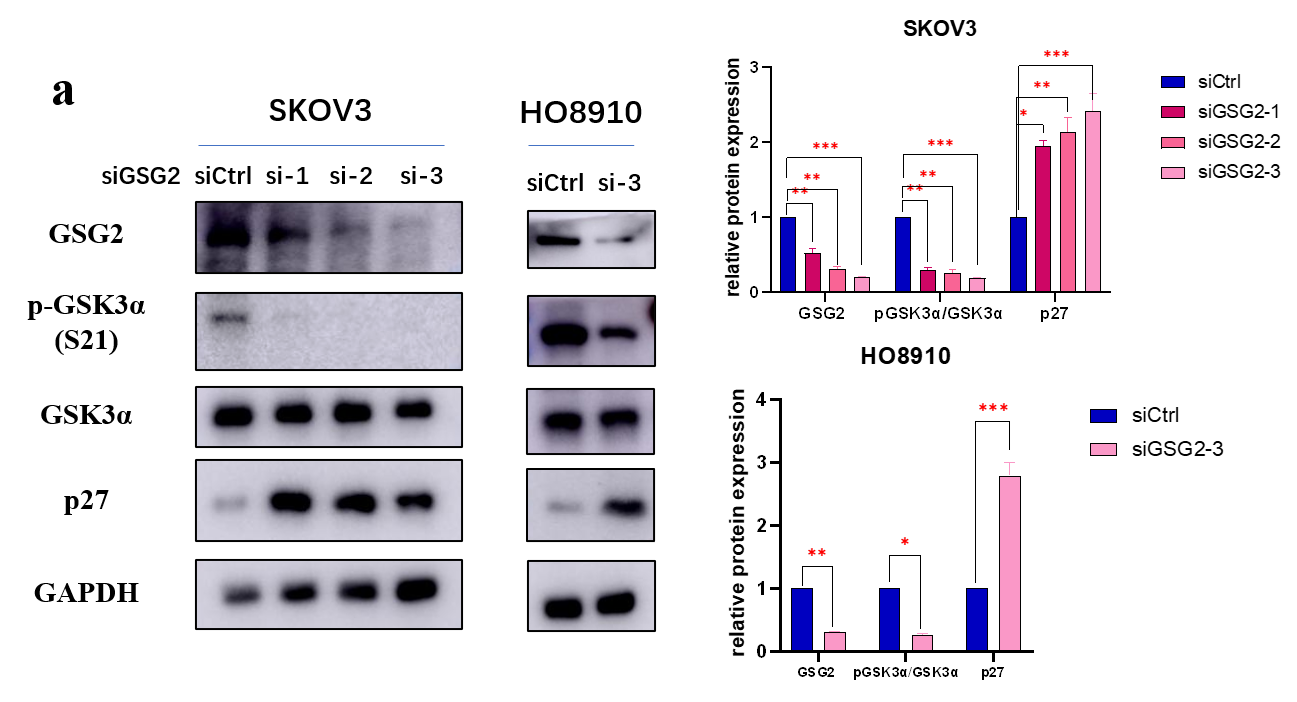

Supplement: Supplementary file 1 — Supplementary file1 (ZIP 11,586 kb) [file 10735_2024_10185_MOESM1_ESM.zip › 10735_2024_10185_MOESM1_ESM/Supplementary Material/Supplementary Figure1_ESM.tif]
