# Supplementary figures and images for: Germ cell-specific gene 2 accelerates cell cycle in epithelial ovarian cancer by inhibiting GSK3α-p27 cascade (part 1 of 2)
Source: J Mol Histol. 2024 Apr 13;55(3):241–51. doi: 10.1007/s10735-024-10185-6 (PMC11102877; doi:10.1007/s10735-024-10185-6)

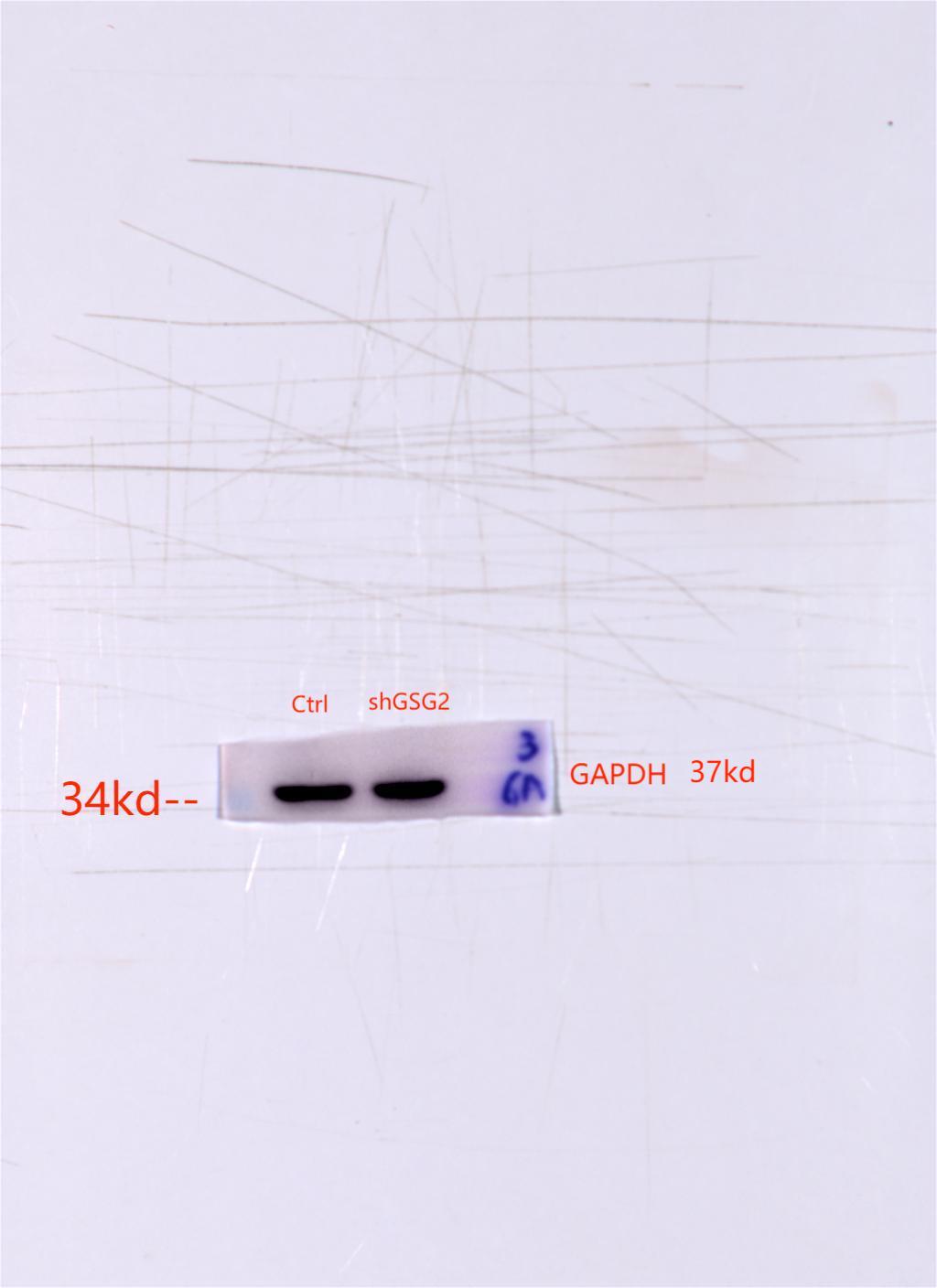

Supplement: Supplementary file 1 — Supplementary file1 (ZIP 11,586 kb) [file 10735_2024_10185_MOESM1_ESM.zip › 10735_2024_10185_MOESM1_ESM/Supplementary Material/Fig1B-HO8910-GAPDH.jpg]

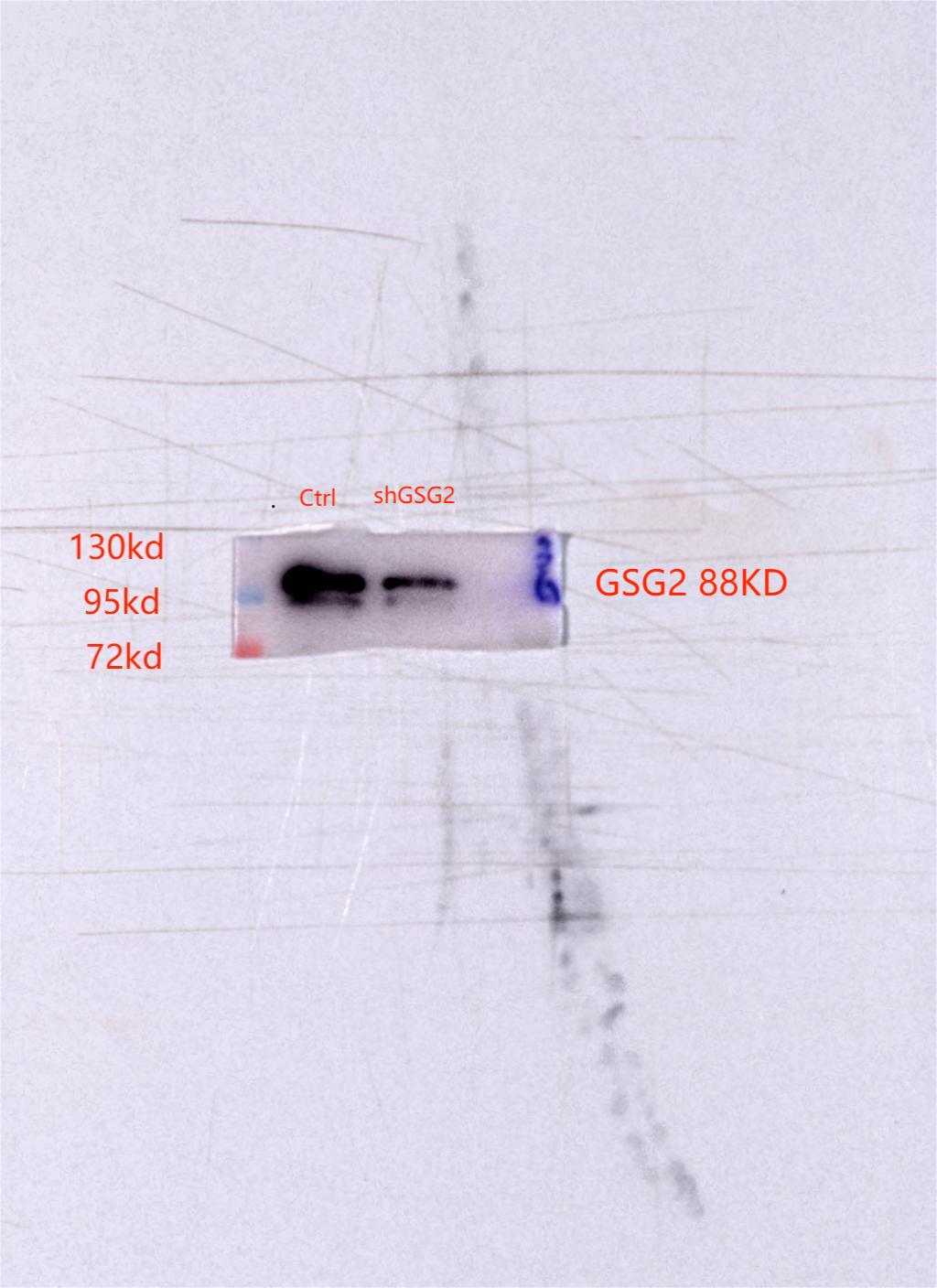

Supplement: Supplementary file 1 — Supplementary file1 (ZIP 11,586 kb) [file 10735_2024_10185_MOESM1_ESM.zip › 10735_2024_10185_MOESM1_ESM/Supplementary Material/Fig1B-HO8910-GSG2 .jpg]

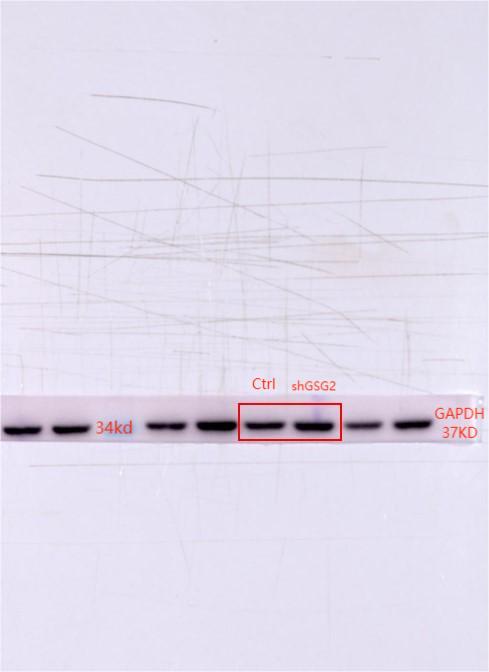

Supplement: Supplementary file 1 — Supplementary file1 (ZIP 11,586 kb) [file 10735_2024_10185_MOESM1_ESM.zip › 10735_2024_10185_MOESM1_ESM/Supplementary Material/Fig1B-SKOV3-GAPDH .jpg]

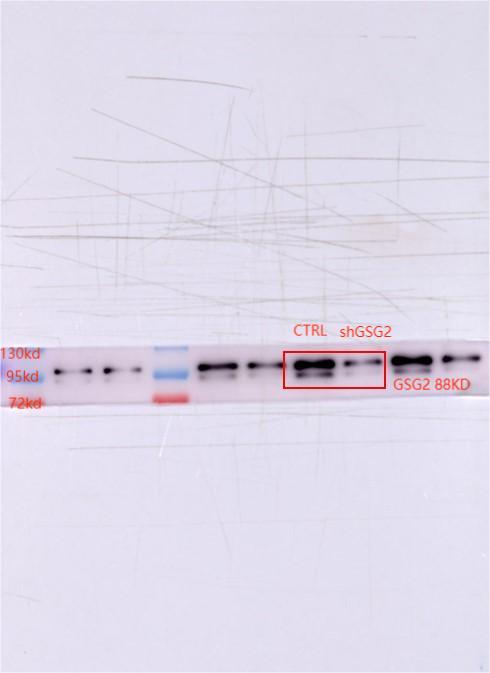

Supplement: Supplementary file 1 — Supplementary file1 (ZIP 11,586 kb) [file 10735_2024_10185_MOESM1_ESM.zip › 10735_2024_10185_MOESM1_ESM/Supplementary Material/Fig1B-SKOV3-GSG2.jpg]

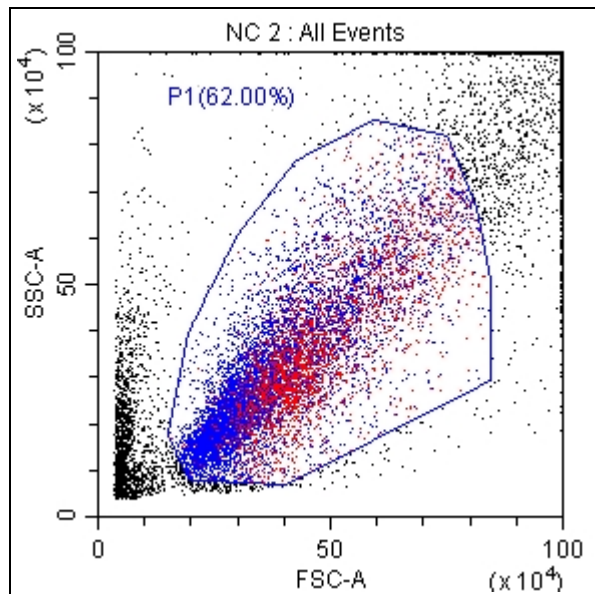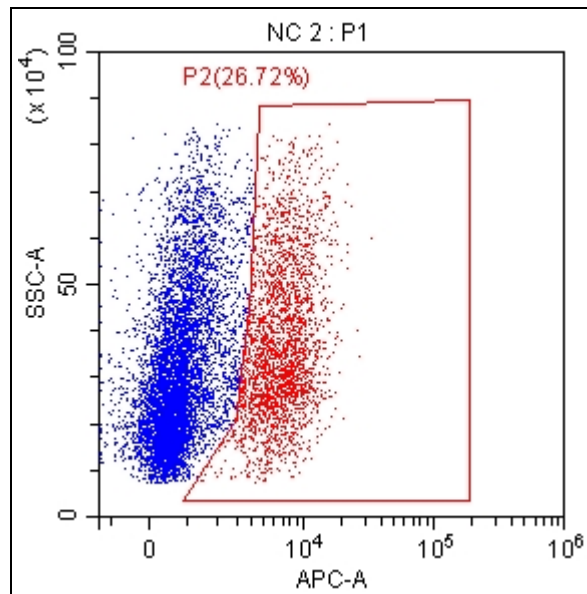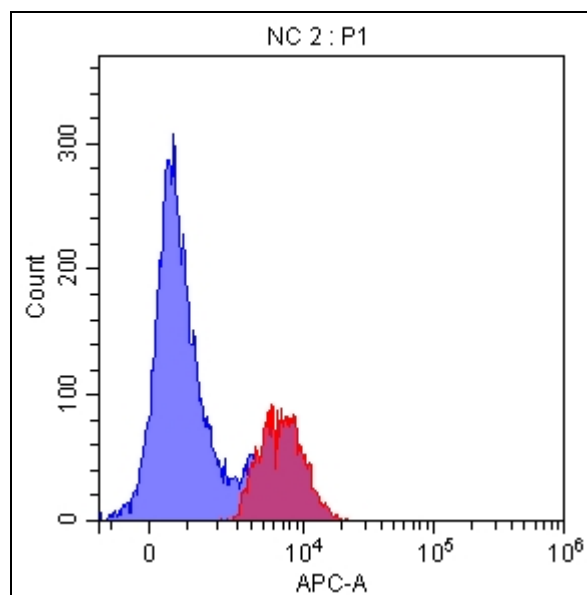

试管名称: NC 2

样本ID:

| 群体           | 颗粒数   | %总数     | %父群     | Mean APC-A |
|--------------|-------|---------|---------|------------|
| ● All Events | 16130 | 100.00% | 100.00% | 9524.2     |
| ● P1         | 10000 | 62.00%  | 62.00%  | 2541.3     |
| ● P2         | 2672  | 16.57%  | 26.72%  | 7361.6     |

Supplement: Supplementary file 1 — Supplementary file1 (ZIP 11,586 kb) [file 10735_2024_10185_MOESM1_ESM.zip › 10735_2024_10185_MOESM1_ESM/Supplementary Material/Fig1C-HO8910-shCtrl.pdf]

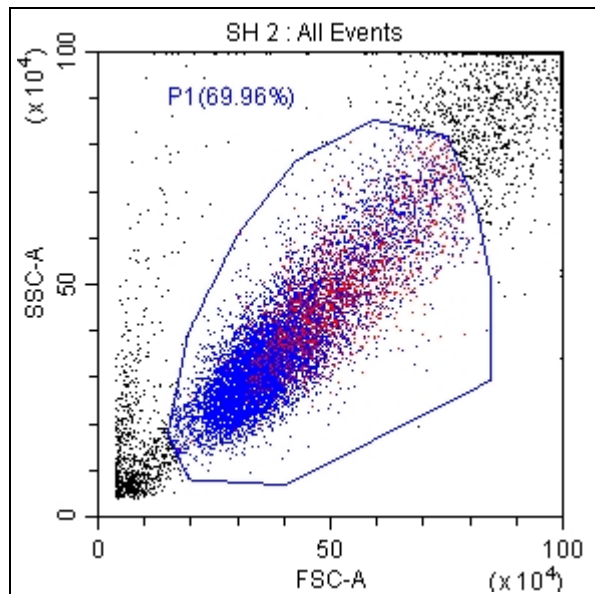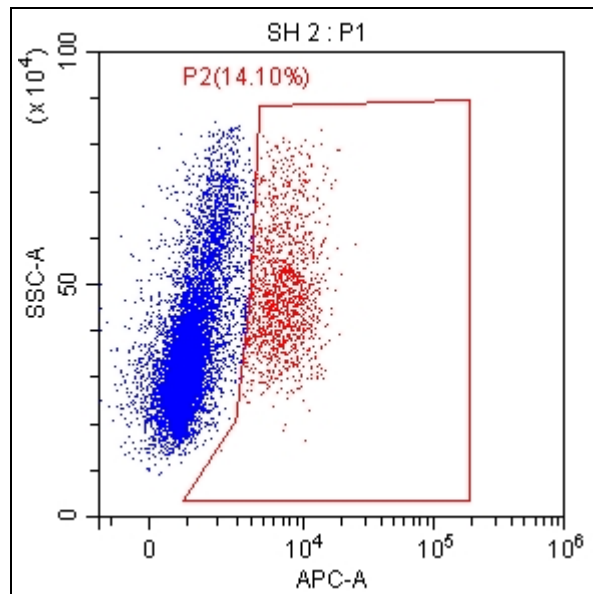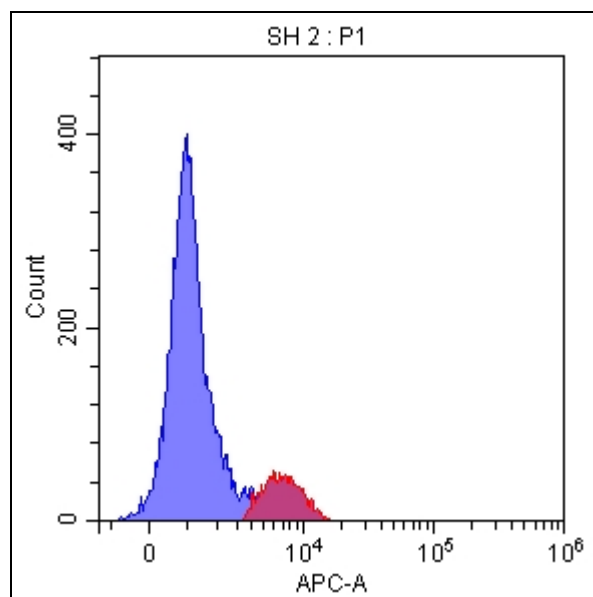

试管名称: SH 2

样本ID:

| 群体           | 颗粒数   | %总数     | %父群     | Mean APC-A |
|--------------|-------|---------|---------|------------|
| ● All Events | 14293 | 100.00% | 100.00% | 6523.5     |
| ● P1         | 10000 | 69.96%  | 69.96%  | 1997.5     |
| ● P2         | 1410  | 9.86%   | 14.10%  | 7441.9     |

Supplement: Supplementary file 1 — Supplementary file1 (ZIP 11,586 kb) [file 10735_2024_10185_MOESM1_ESM.zip › 10735_2024_10185_MOESM1_ESM/Supplementary Material/Fig1C-HO8910-shGSG2.pdf]

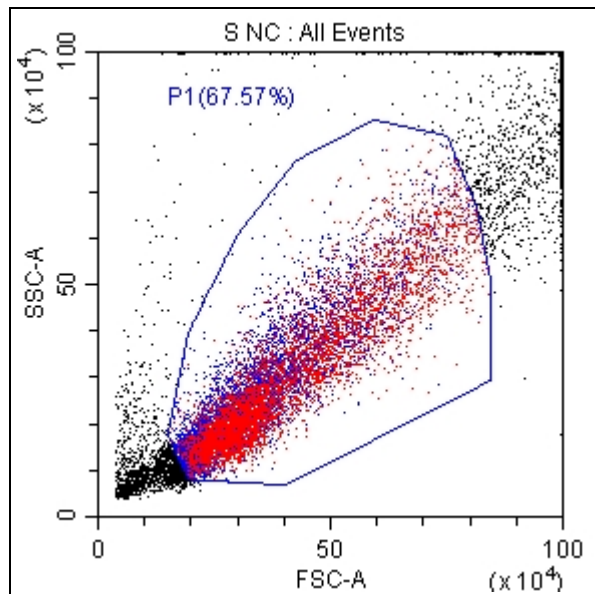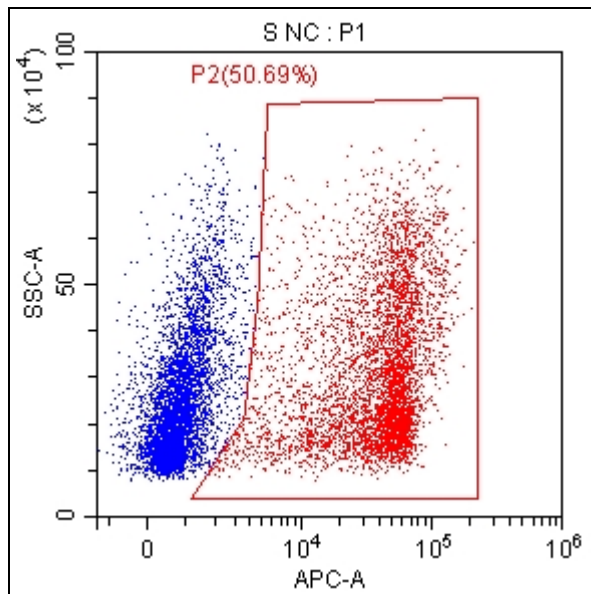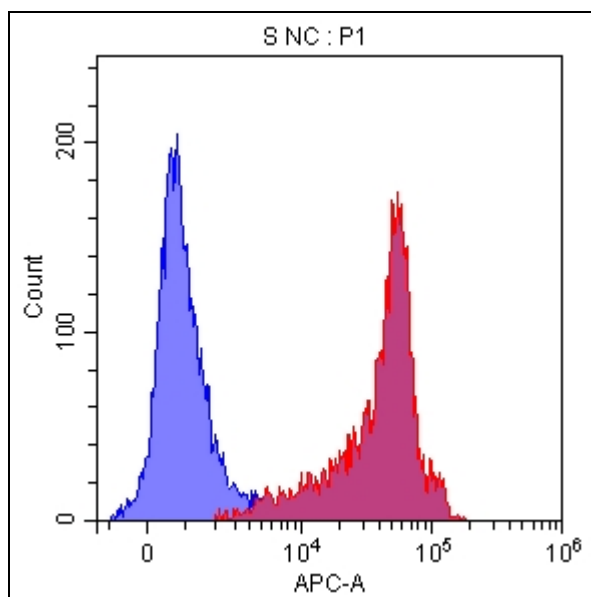

试管名称: S NC

样本ID:

| 群体           | 颗粒数   | %总数     | %父群     | Mean APC-A |
|--------------|-------|---------|---------|------------|
| ● All Events | 14800 | 100.00% | 100.00% | 49097.9    |
| ● P1         | 10000 | 67.57%  | 67.57%  | 23445.2    |
| ● P2         | 5069  | 34.25%  | 50.69%  | 45377.3    |

Supplement: Supplementary file 1 — Supplementary file1 (ZIP 11,586 kb) [file 10735_2024_10185_MOESM1_ESM.zip › 10735_2024_10185_MOESM1_ESM/Supplementary Material/Fig1C-SKOV3-shCtrl.pdf]

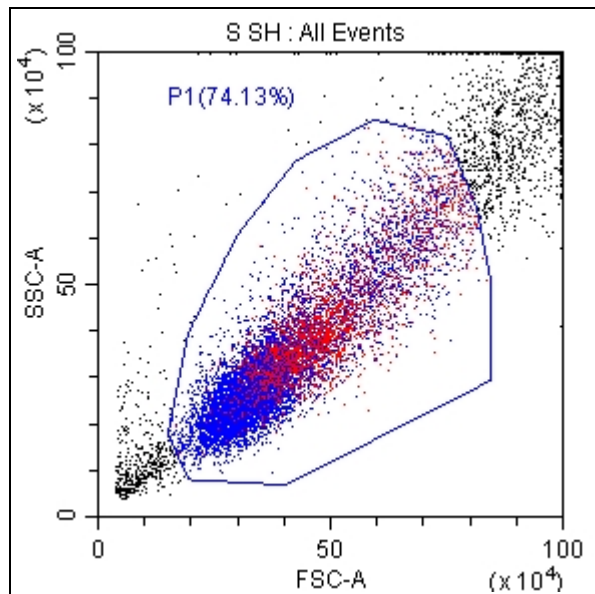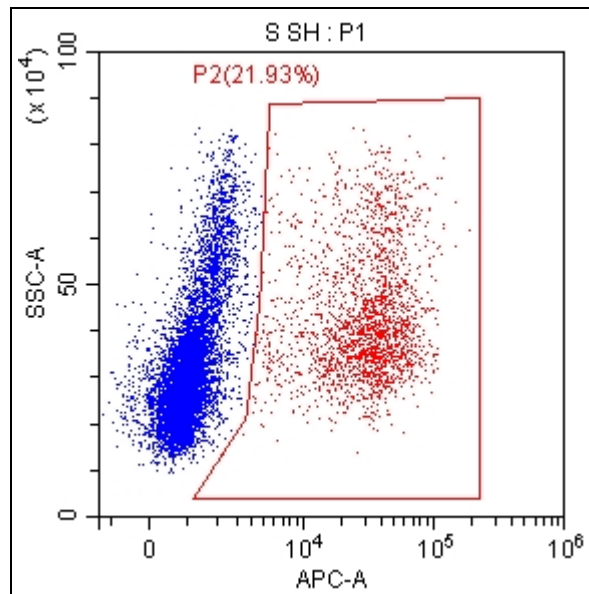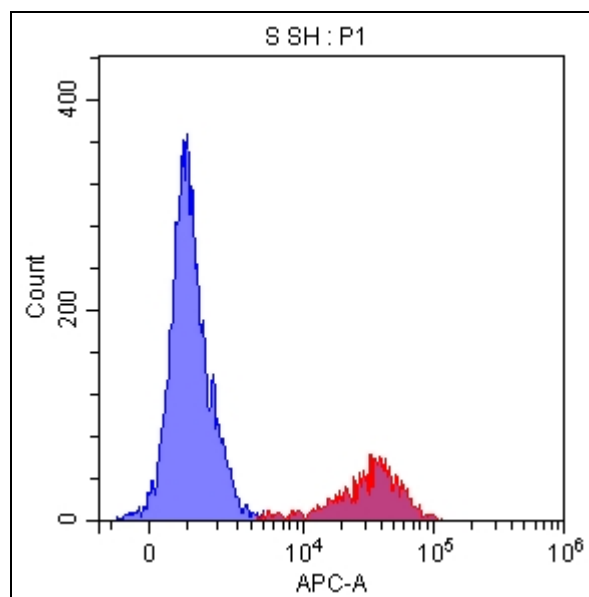

试管名称: S SH

样本ID:

| 群体           | 颗粒数   | %总数     | %父群     | Mean APC-A |
|--------------|-------|---------|---------|------------|
| ● All Events | 13489 | 100.00% | 100.00% | 26260.8    |
| ● P1         | 10000 | 74.13%  | 74.13%  | 8613.3     |
| ● P2         | 2193  | 16.26%  | 21.93%  | 35310.9    |

Supplement: Supplementary file 1 — Supplementary file1 (ZIP 11,586 kb) [file 10735_2024_10185_MOESM1_ESM.zip › 10735_2024_10185_MOESM1_ESM/Supplementary Material/Fig1C-SKOV3-shGSG2.pdf]

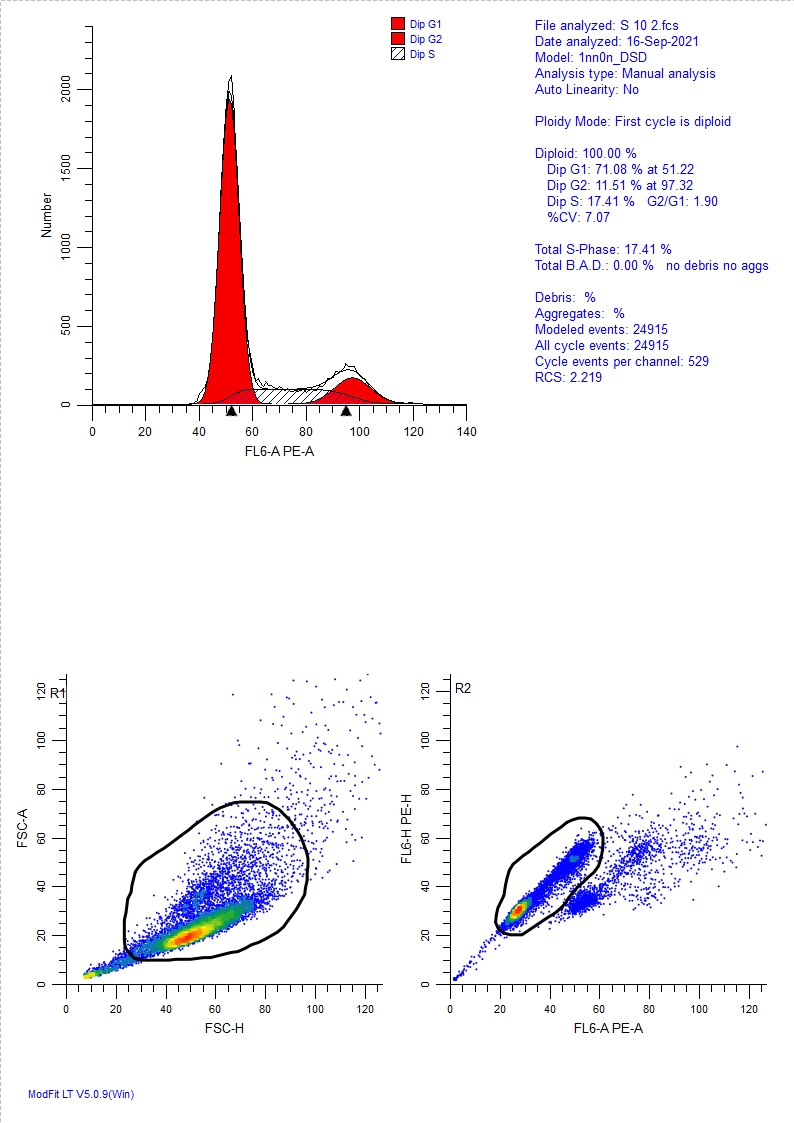

Supplement: Supplementary file 1 — Supplementary file1 (ZIP 11,586 kb) [file 10735_2024_10185_MOESM1_ESM.zip › 10735_2024_10185_MOESM1_ESM/Supplementary Material/Fig1D-HO8910-shCtrl.jpg]

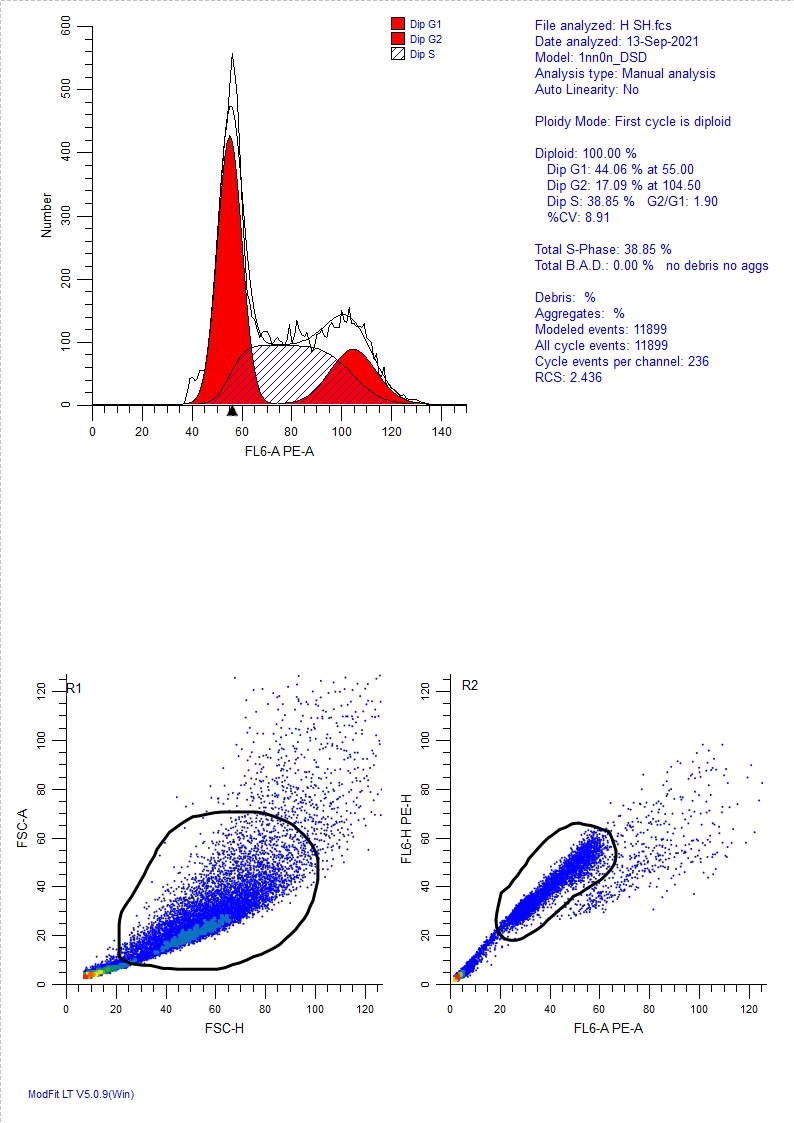

Supplement: Supplementary file 1 — Supplementary file1 (ZIP 11,586 kb) [file 10735_2024_10185_MOESM1_ESM.zip › 10735_2024_10185_MOESM1_ESM/Supplementary Material/Fig1D-HO8910-shGSG2.jpg]

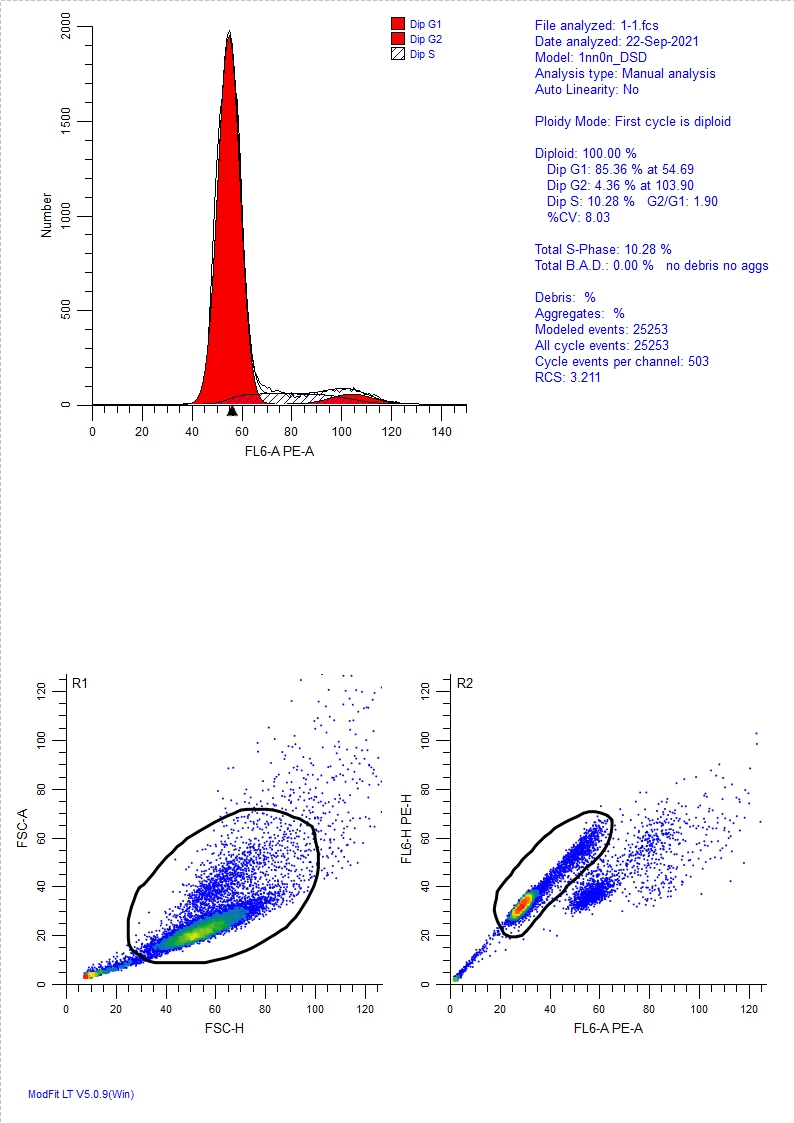

Supplement: Supplementary file 1 — Supplementary file1 (ZIP 11,586 kb) [file 10735_2024_10185_MOESM1_ESM.zip › 10735_2024_10185_MOESM1_ESM/Supplementary Material/Fig1D-SKOV3-Ctrl.jpg]

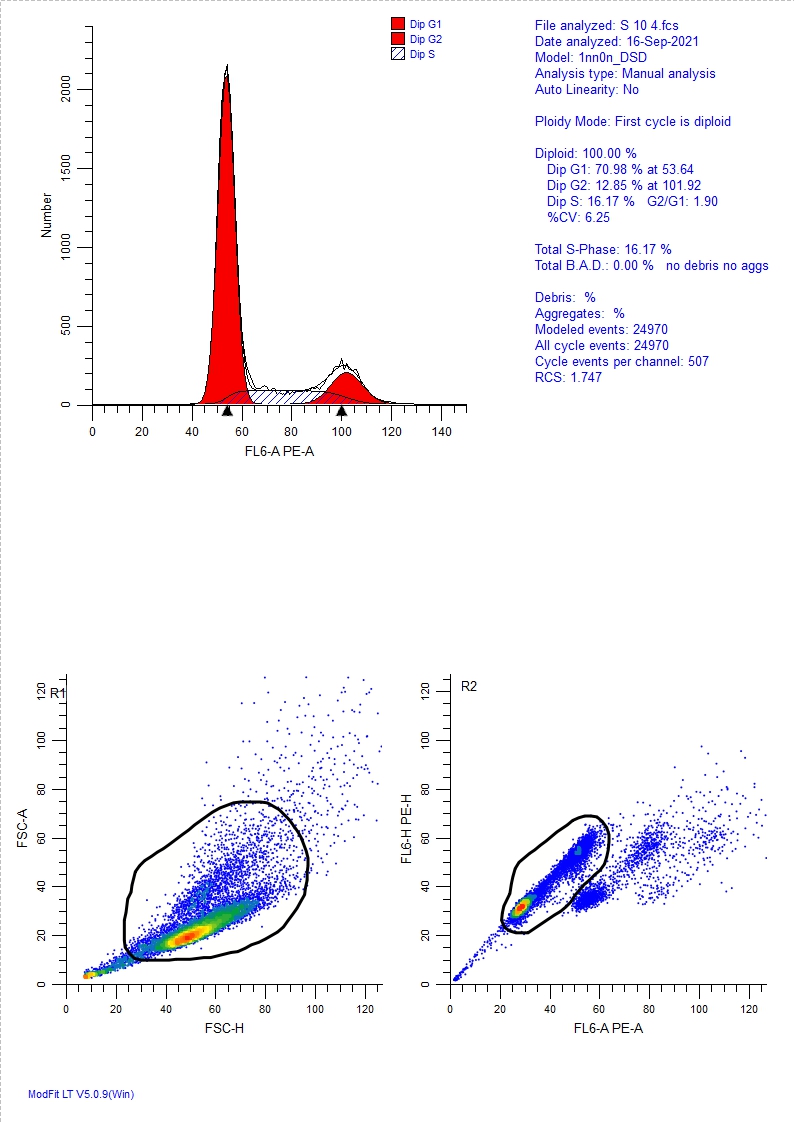

Supplement: Supplementary file 1 — Supplementary file1 (ZIP 11,586 kb) [file 10735_2024_10185_MOESM1_ESM.zip › 10735_2024_10185_MOESM1_ESM/Supplementary Material/Fig1D-SKOV3-shGSG2 .jpg]

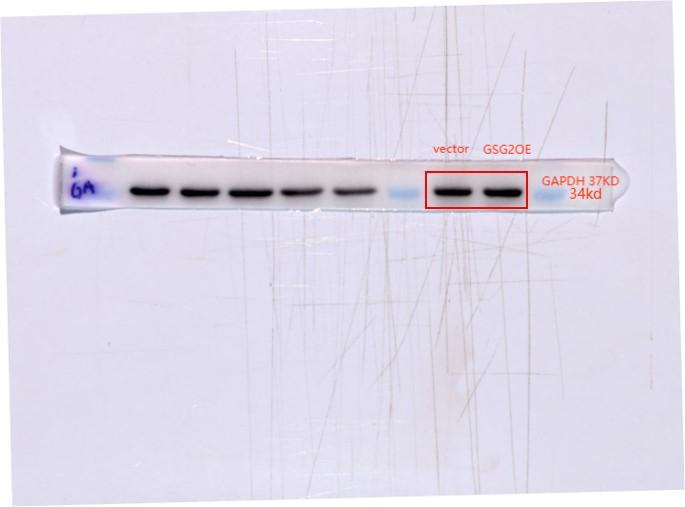

Supplement: Supplementary file 1 — Supplementary file1 (ZIP 11,586 kb) [file 10735_2024_10185_MOESM1_ESM.zip › 10735_2024_10185_MOESM1_ESM/Supplementary Material/Fig2B-HO8910-GAPDH.jpg]

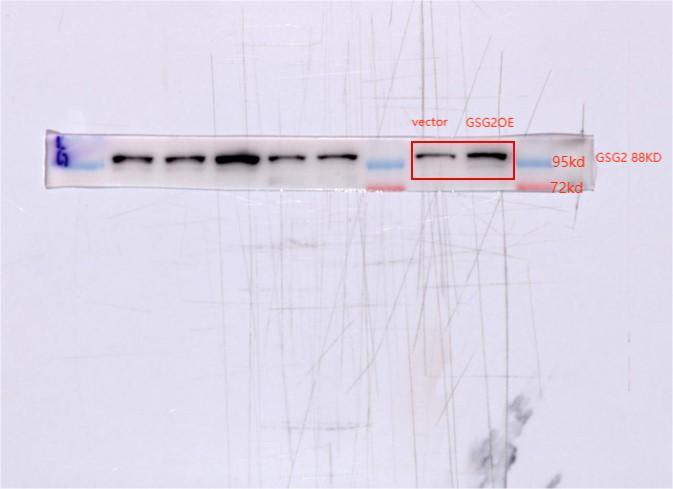

Supplement: Supplementary file 1 — Supplementary file1 (ZIP 11,586 kb) [file 10735_2024_10185_MOESM1_ESM.zip › 10735_2024_10185_MOESM1_ESM/Supplementary Material/Fig2B-HO8910-GSG2.jpg]

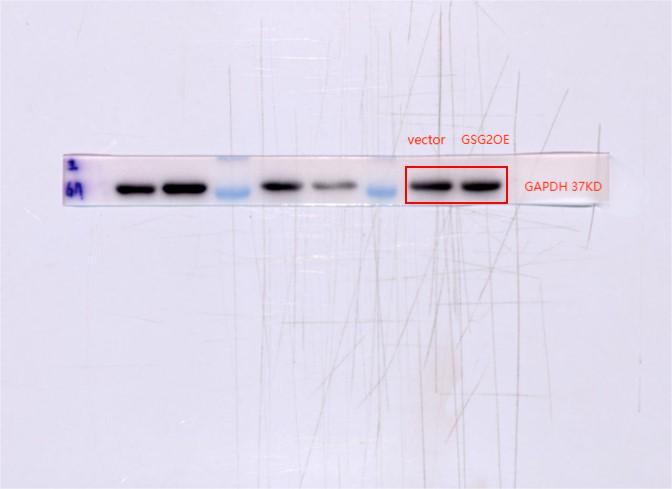

Supplement: Supplementary file 1 — Supplementary file1 (ZIP 11,586 kb) [file 10735_2024_10185_MOESM1_ESM.zip › 10735_2024_10185_MOESM1_ESM/Supplementary Material/Fig2B-SKOV3-GAPDH.jpg]

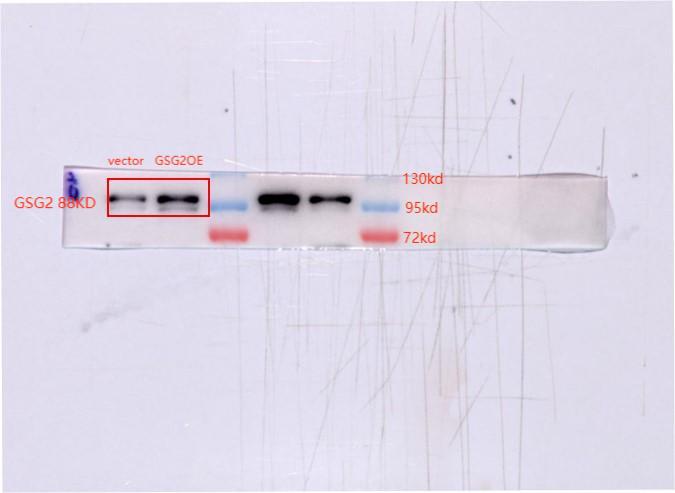

Supplement: Supplementary file 1 — Supplementary file1 (ZIP 11,586 kb) [file 10735_2024_10185_MOESM1_ESM.zip › 10735_2024_10185_MOESM1_ESM/Supplementary Material/Fig2B-SKOV3-GSG2.jpg]

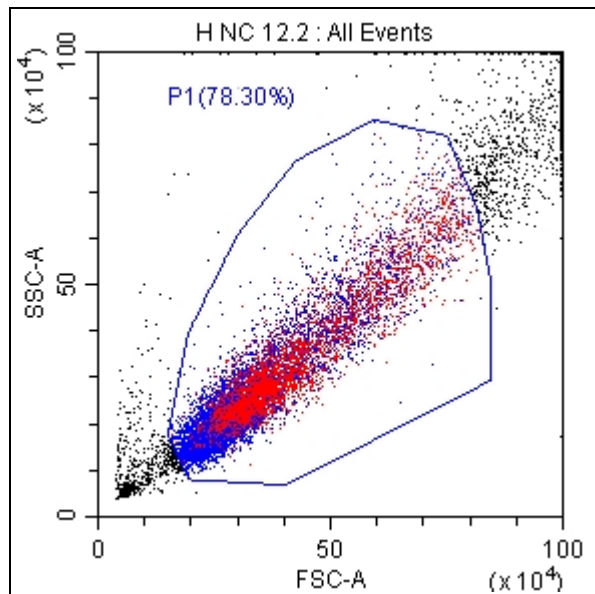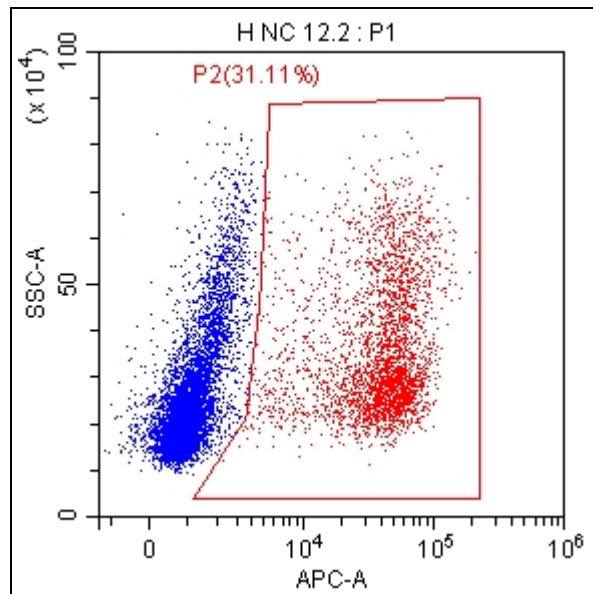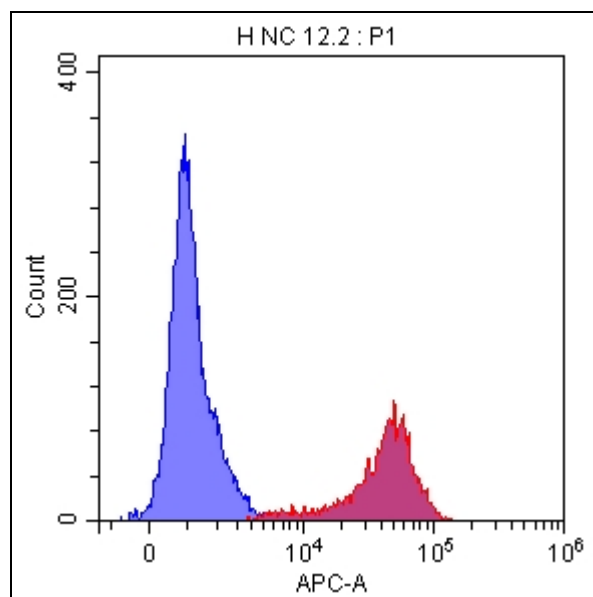

试管名称: H NC 12.2

样本ID:

| 群体           | 颗粒数   | %总数     | %父群     | Mean APC-A |
|--------------|-------|---------|---------|------------|
| ● All Events | 13015 | 100.00% | 100.00% | 34642.9    |
| ● P1         | 10191 | 78.30%  | 78.30%  | 14586.1    |
| ● P2         | 3170  | 24.36%  | 31.11%  | 44356.7    |

Supplement: Supplementary file 1 — Supplementary file1 (ZIP 11,586 kb) [file 10735_2024_10185_MOESM1_ESM.zip › 10735_2024_10185_MOESM1_ESM/Supplementary Material/Fig2C-HO8910-GSG2OE.pdf]

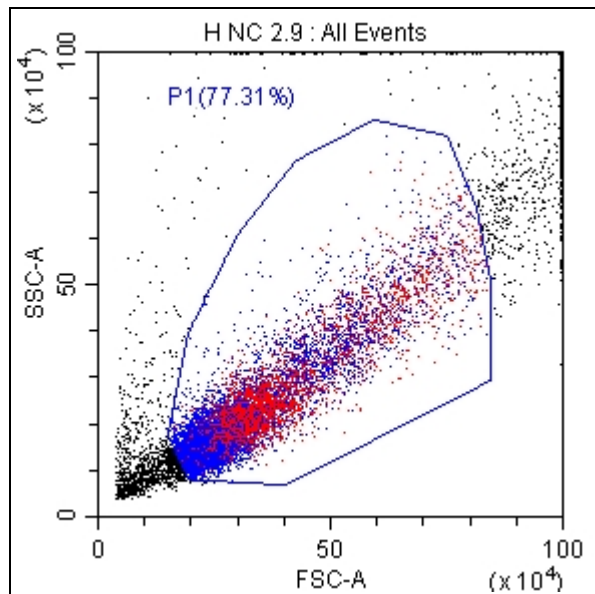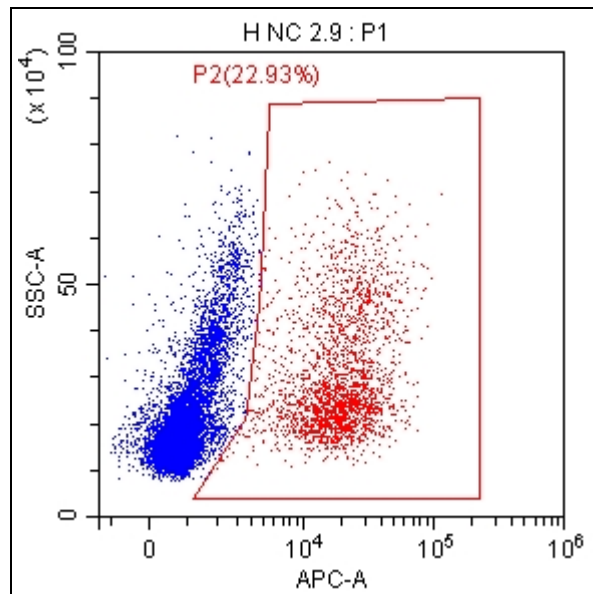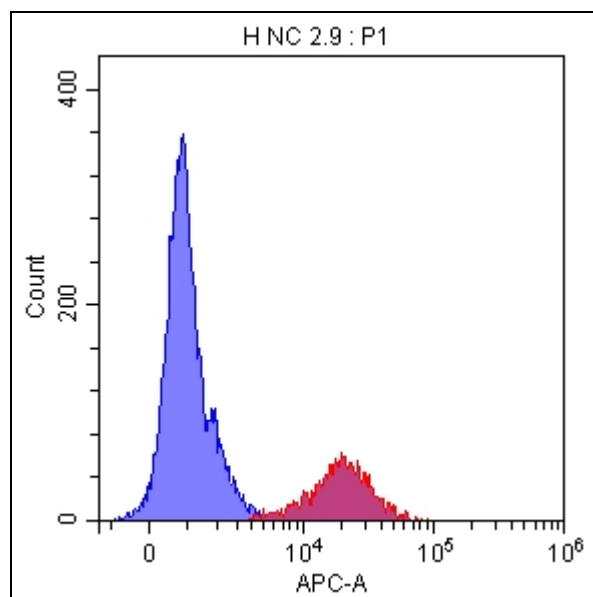

试管名称: H NC 2.9

样本ID:

| 群体           | 颗粒数   | %总数     | %父群     | Mean APC-A |
|--------------|-------|---------|---------|------------|
| ● All Events | 12935 | 100.00% | 100.00% | 11555.0    |
| ● P1         | 10000 | 77.31%  | 77.31%  | 5789.7     |
| ● P2         | 2293  | 17.73%  | 22.93%  | 21829.4    |

Supplement: Supplementary file 1 — Supplementary file1 (ZIP 11,586 kb) [file 10735_2024_10185_MOESM1_ESM.zip › 10735_2024_10185_MOESM1_ESM/Supplementary Material/Fig2C-SKOV3-vector.pdf]

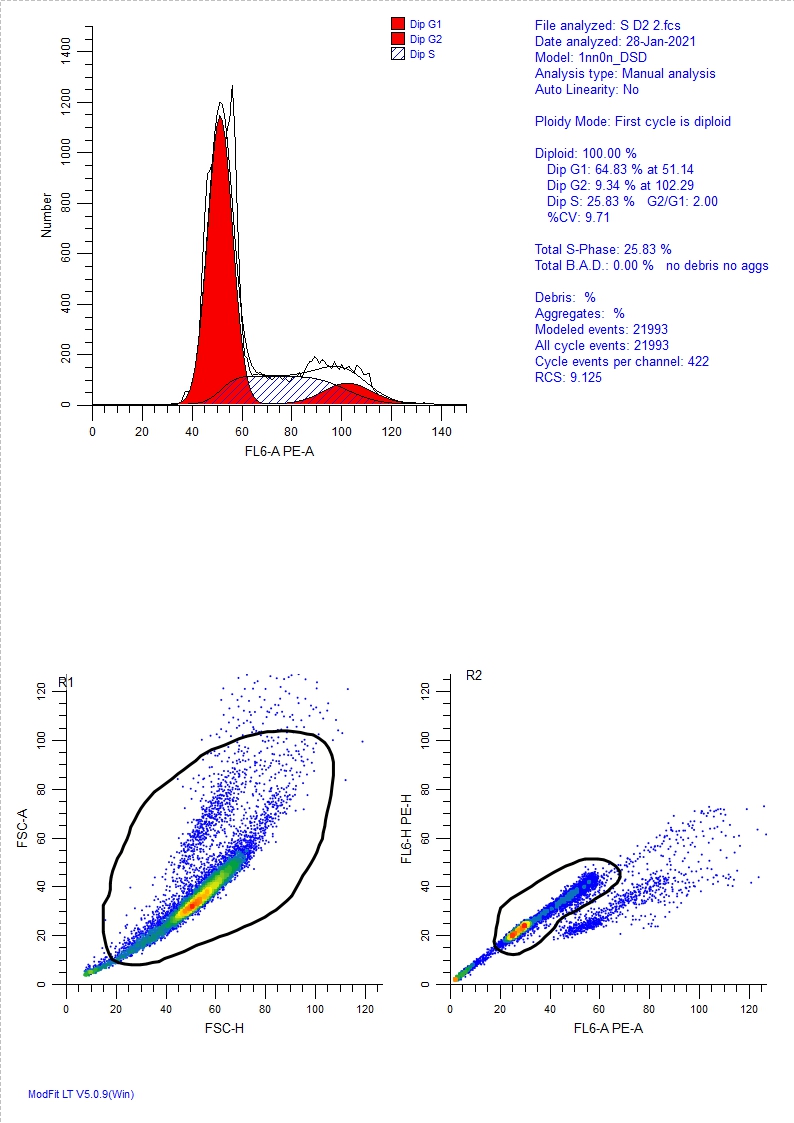

Supplement: Supplementary file 1 — Supplementary file1 (ZIP 11,586 kb) [file 10735_2024_10185_MOESM1_ESM.zip › 10735_2024_10185_MOESM1_ESM/Supplementary Material/Fig2D-HO8910-GSG2OE.jpg]

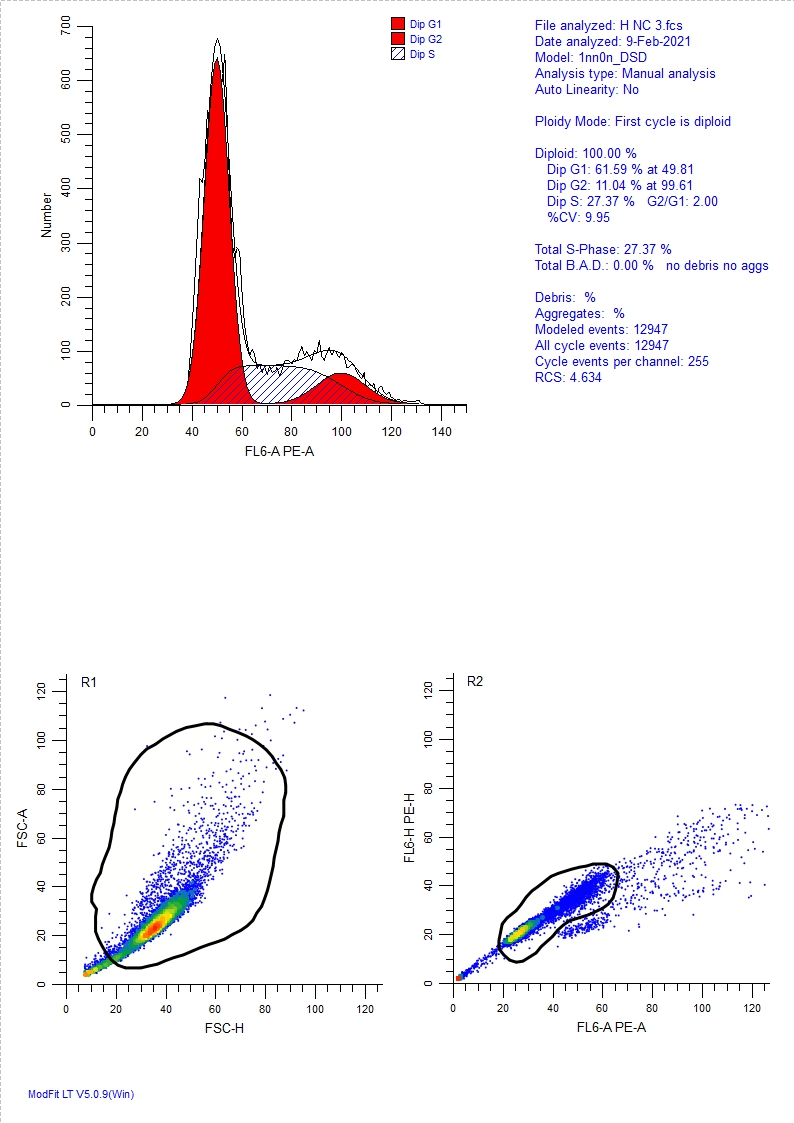

Supplement: Supplementary file 1 — Supplementary file1 (ZIP 11,586 kb) [file 10735_2024_10185_MOESM1_ESM.zip › 10735_2024_10185_MOESM1_ESM/Supplementary Material/Fig2D-HO8910-vector.jpg]

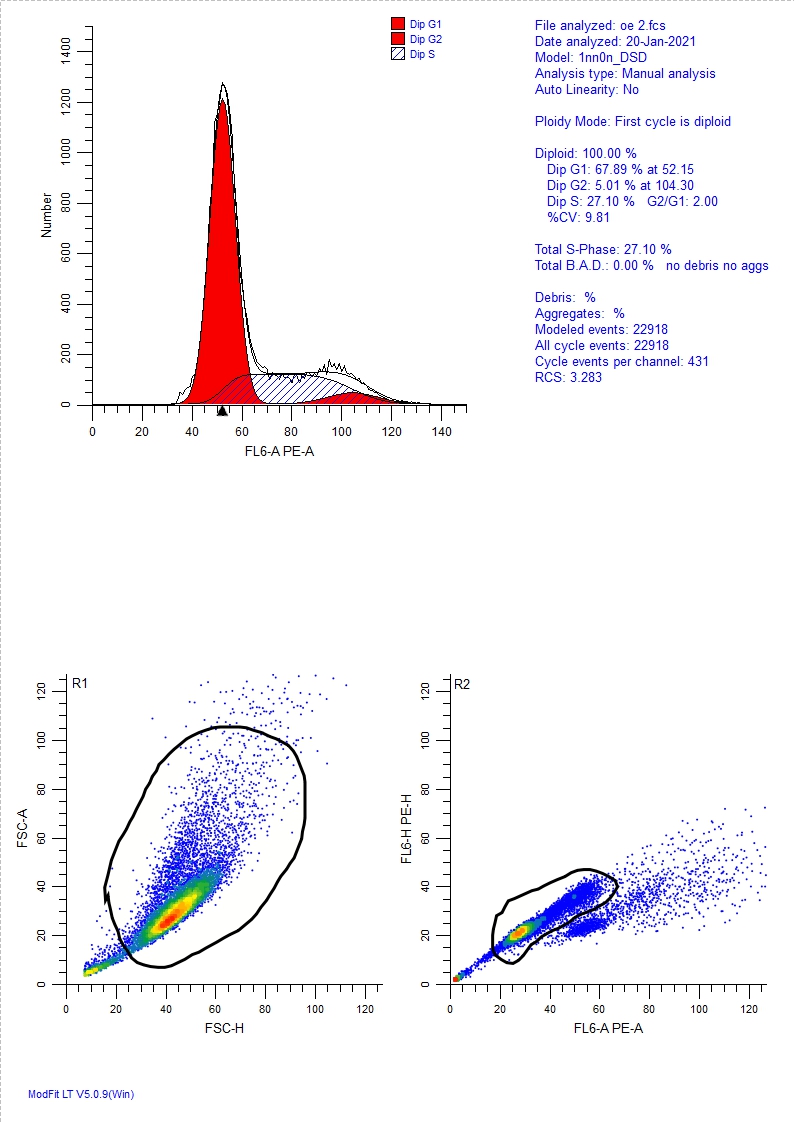

Supplement: Supplementary file 1 — Supplementary file1 (ZIP 11,586 kb) [file 10735_2024_10185_MOESM1_ESM.zip › 10735_2024_10185_MOESM1_ESM/Supplementary Material/Fig2D-SKOV3-GSG2OE.jpg]

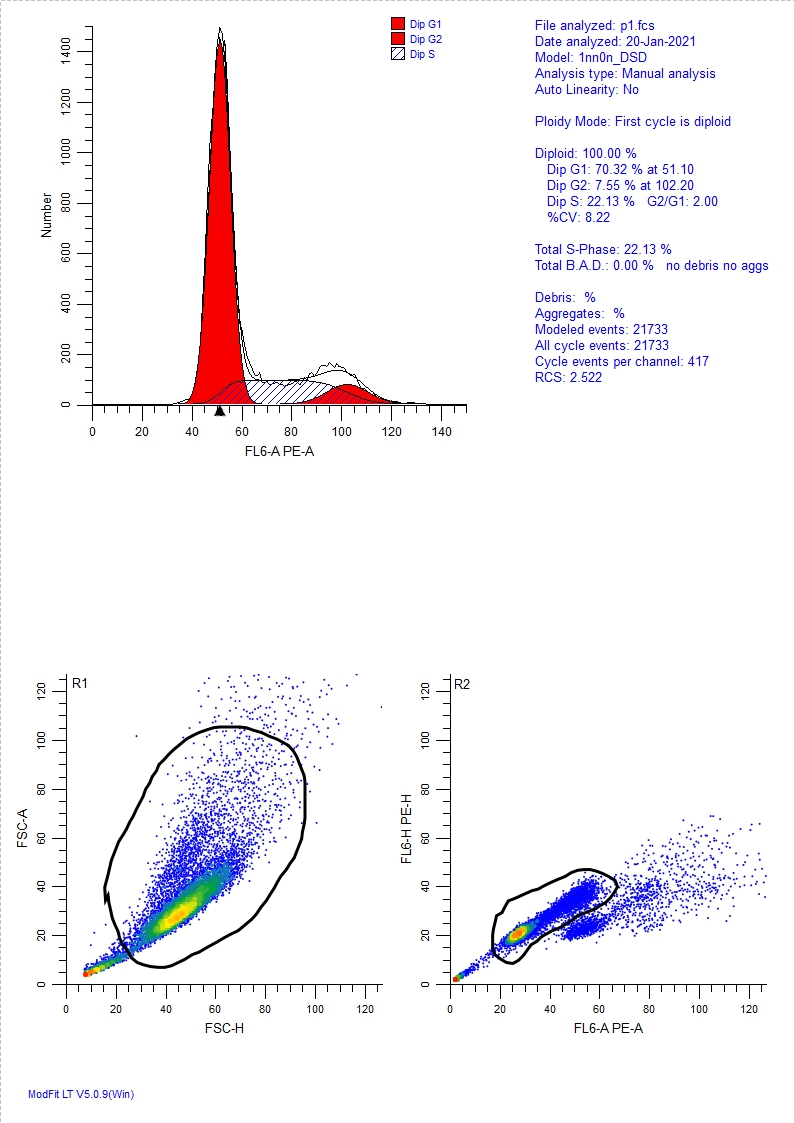

Supplement: Supplementary file 1 — Supplementary file1 (ZIP 11,586 kb) [file 10735_2024_10185_MOESM1_ESM.zip › 10735_2024_10185_MOESM1_ESM/Supplementary Material/Fig2D-SKOV3-vector.jpg]

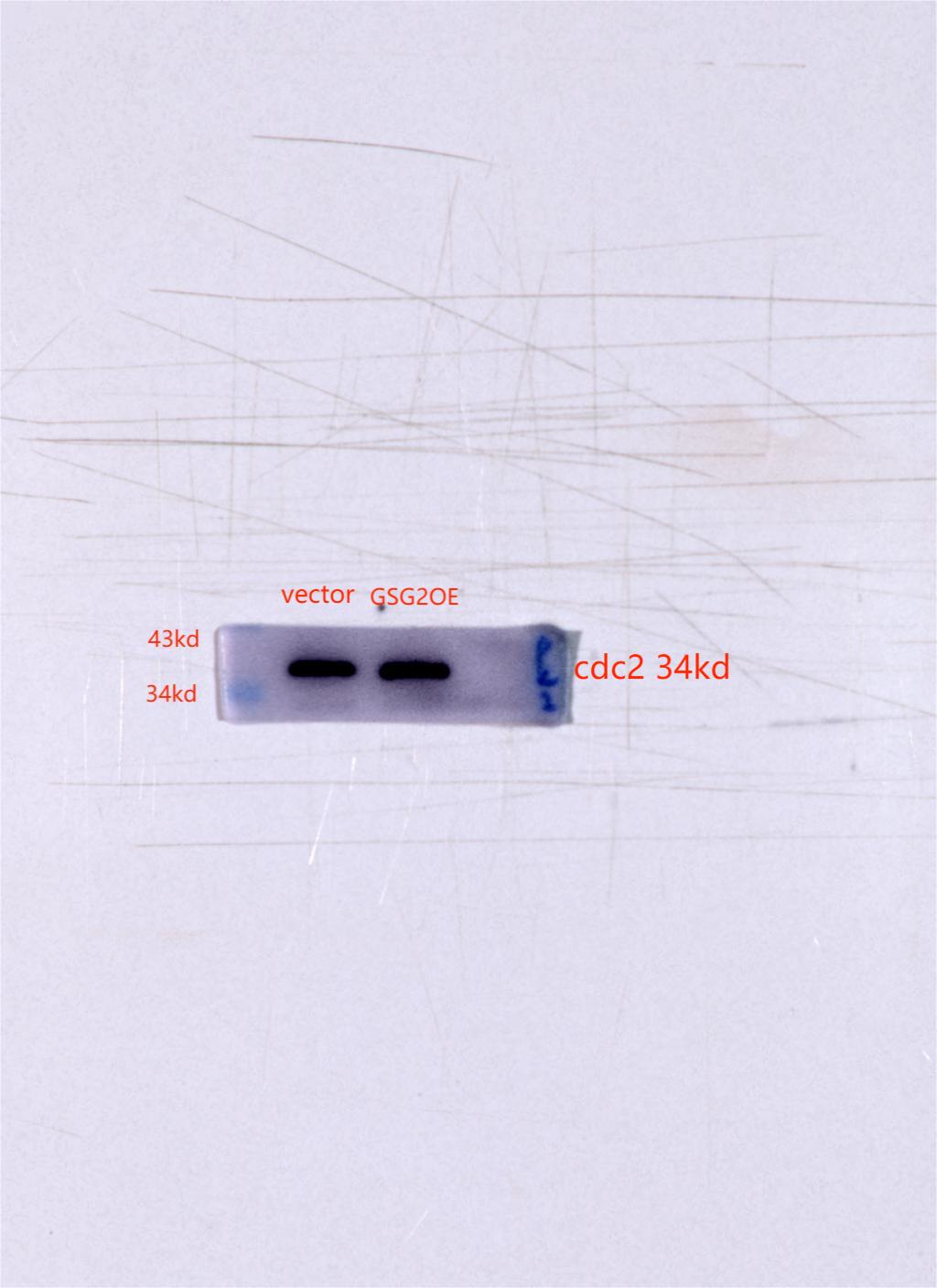

Supplement: Supplementary file 1 — Supplementary file1 (ZIP 11,586 kb) [file 10735_2024_10185_MOESM1_ESM.zip › 10735_2024_10185_MOESM1_ESM/Supplementary Material/Fig3B-HO8910-OE-cdc2.jpg]

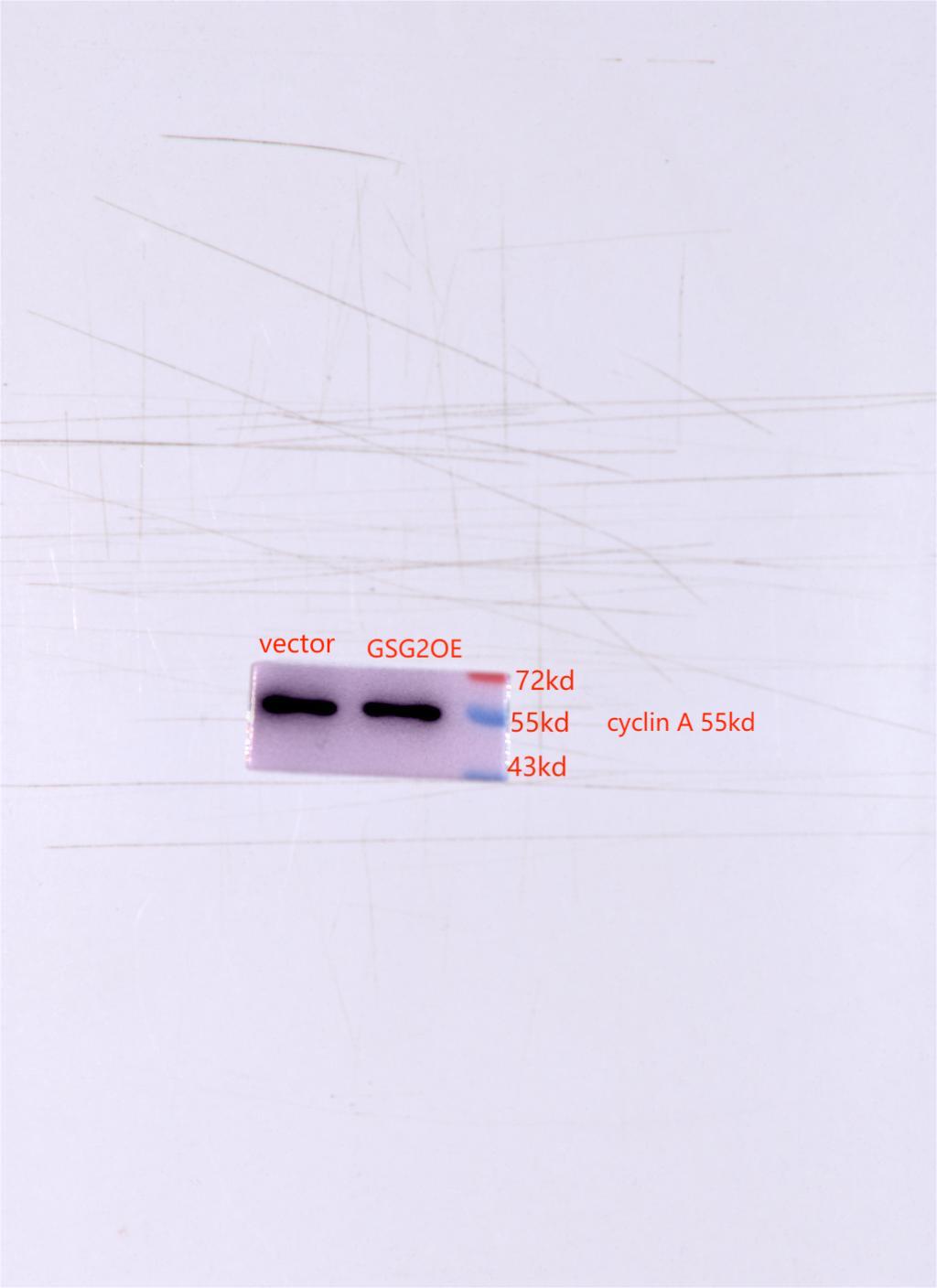

Supplement: Supplementary file 1 — Supplementary file1 (ZIP 11,586 kb) [file 10735_2024_10185_MOESM1_ESM.zip › 10735_2024_10185_MOESM1_ESM/Supplementary Material/Fig3B-HO8910-OE-cyclin A.jpg]

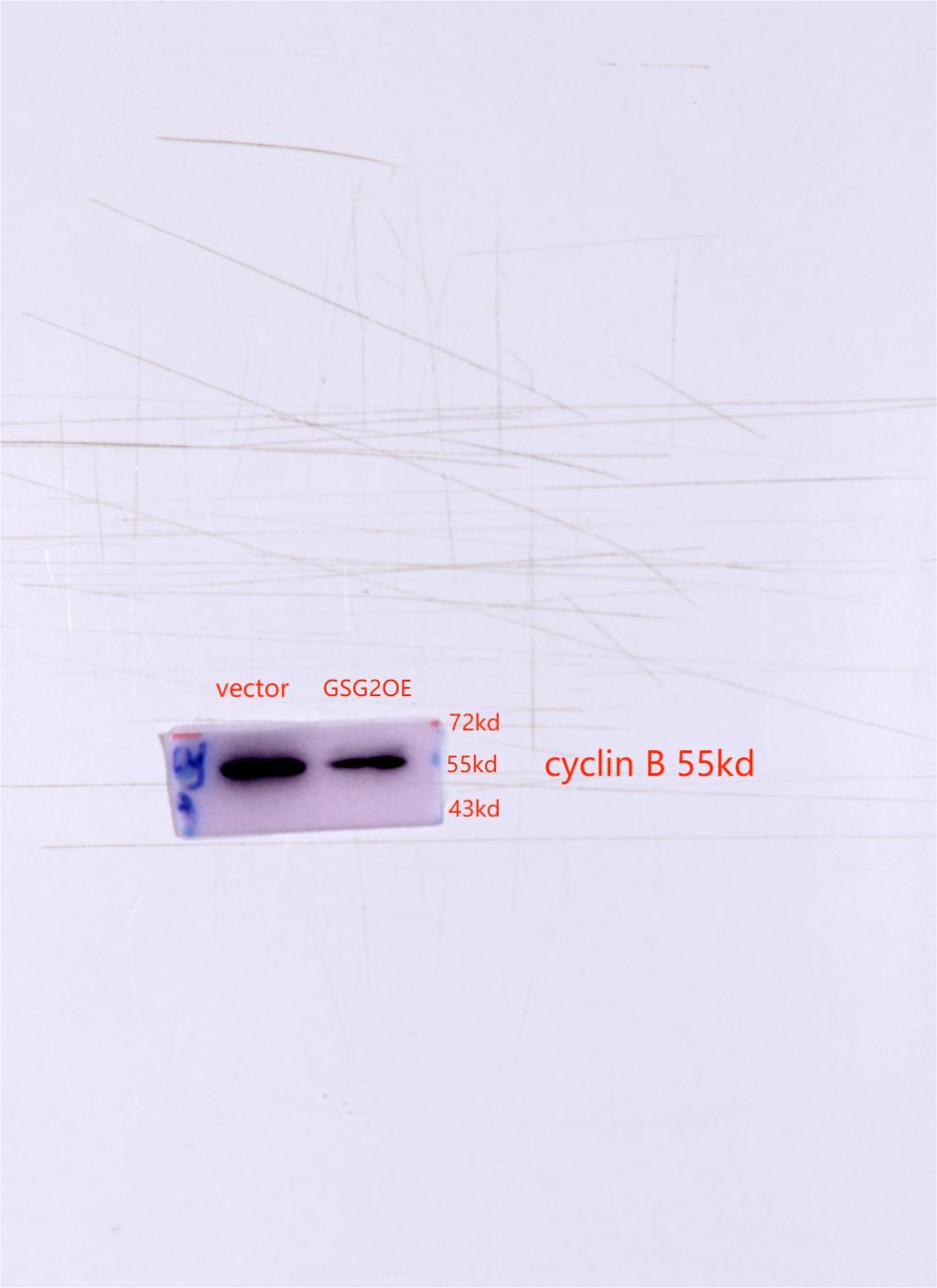

Supplement: Supplementary file 1 — Supplementary file1 (ZIP 11,586 kb) [file 10735_2024_10185_MOESM1_ESM.zip › 10735_2024_10185_MOESM1_ESM/Supplementary Material/Fig3B-HO8910-OE-cyclin B.jpg]

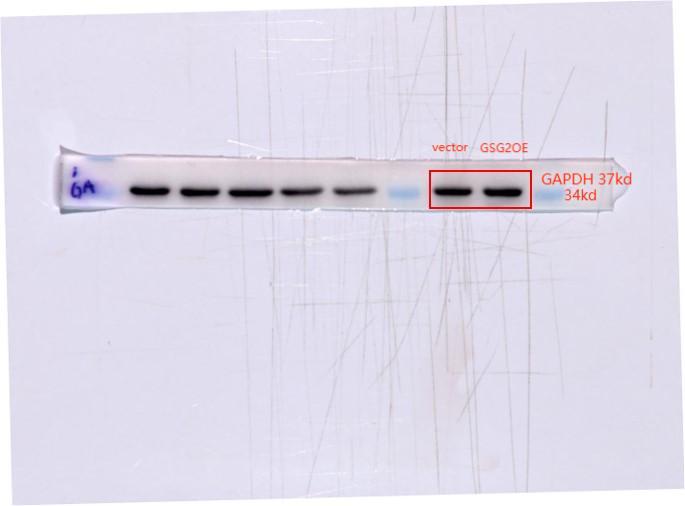

Supplement: Supplementary file 1 — Supplementary file1 (ZIP 11,586 kb) [file 10735_2024_10185_MOESM1_ESM.zip › 10735_2024_10185_MOESM1_ESM/Supplementary Material/Fig3B-HO8910-OE-GAPDH.jpg]

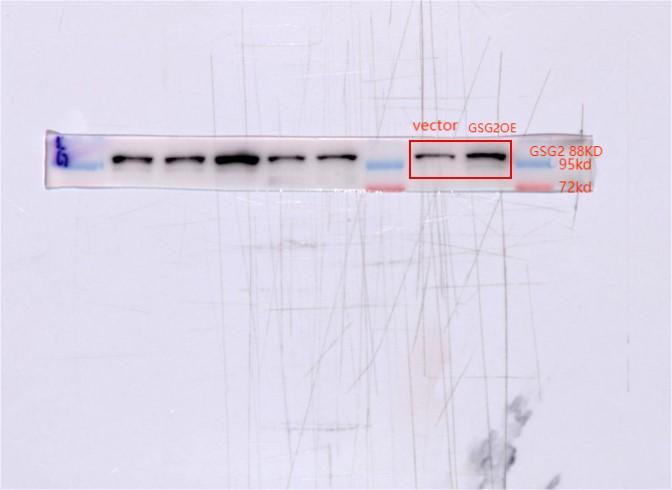

Supplement: Supplementary file 1 — Supplementary file1 (ZIP 11,586 kb) [file 10735_2024_10185_MOESM1_ESM.zip › 10735_2024_10185_MOESM1_ESM/Supplementary Material/Fig3B-HO8910-OE-GSG2.jpg]

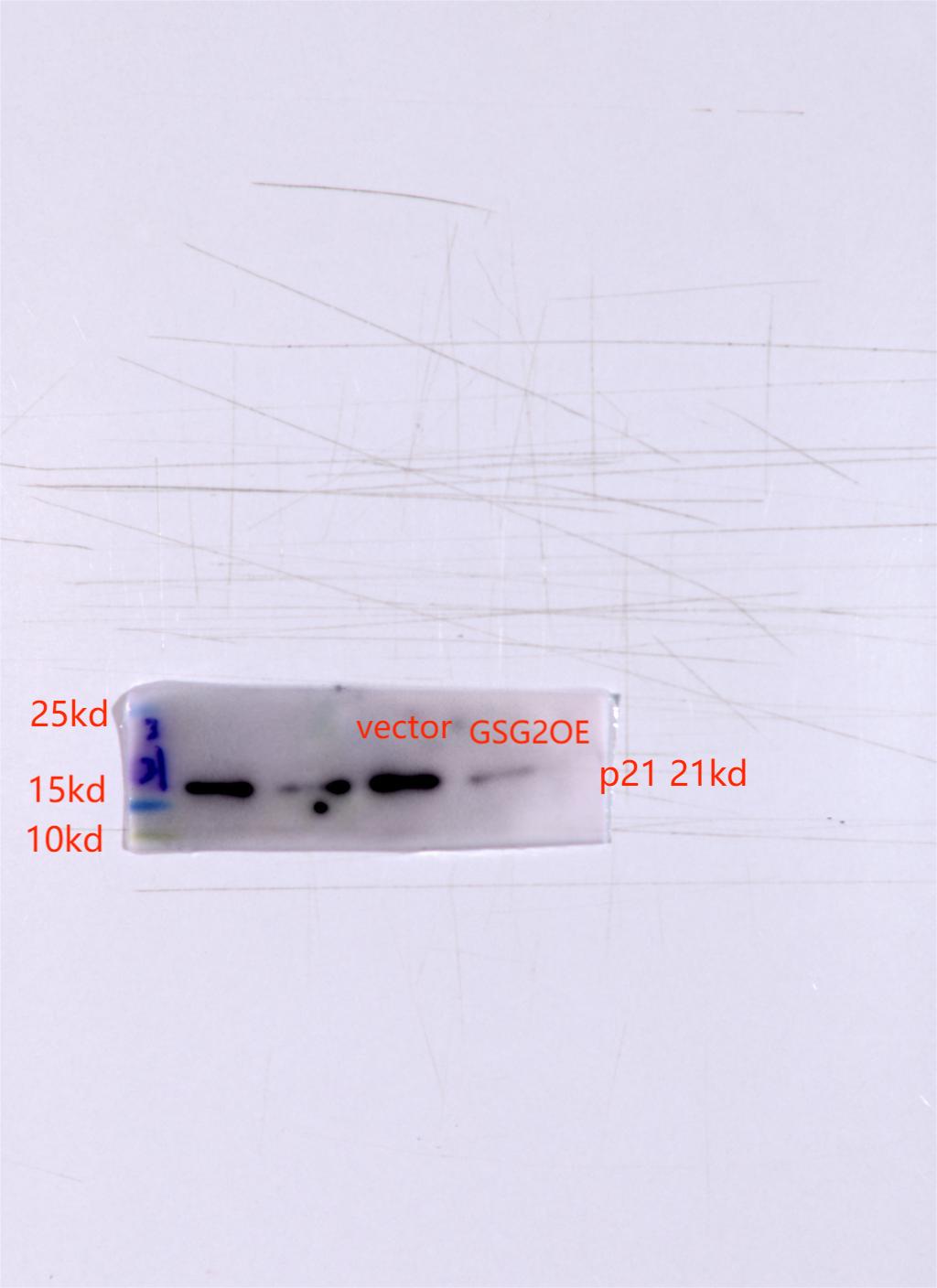

Supplement: Supplementary file 1 — Supplementary file1 (ZIP 11,586 kb) [file 10735_2024_10185_MOESM1_ESM.zip › 10735_2024_10185_MOESM1_ESM/Supplementary Material/Fig3B-HO8910-OE-p21.jpg]

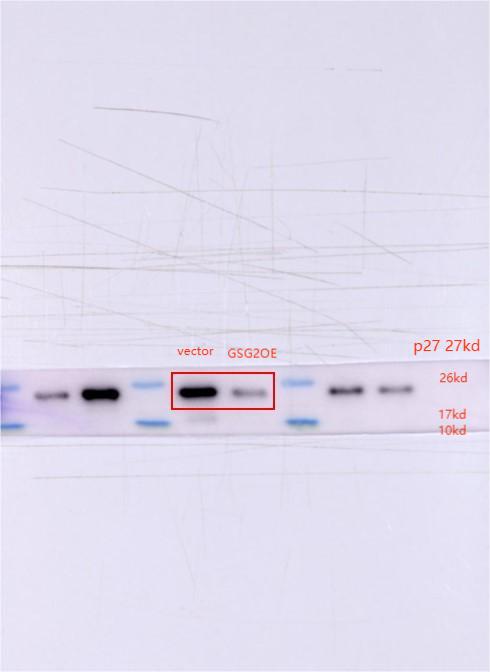

Supplement: Supplementary file 1 — Supplementary file1 (ZIP 11,586 kb) [file 10735_2024_10185_MOESM1_ESM.zip › 10735_2024_10185_MOESM1_ESM/Supplementary Material/Fig3B-HO8910-OE-p27 .jpg]

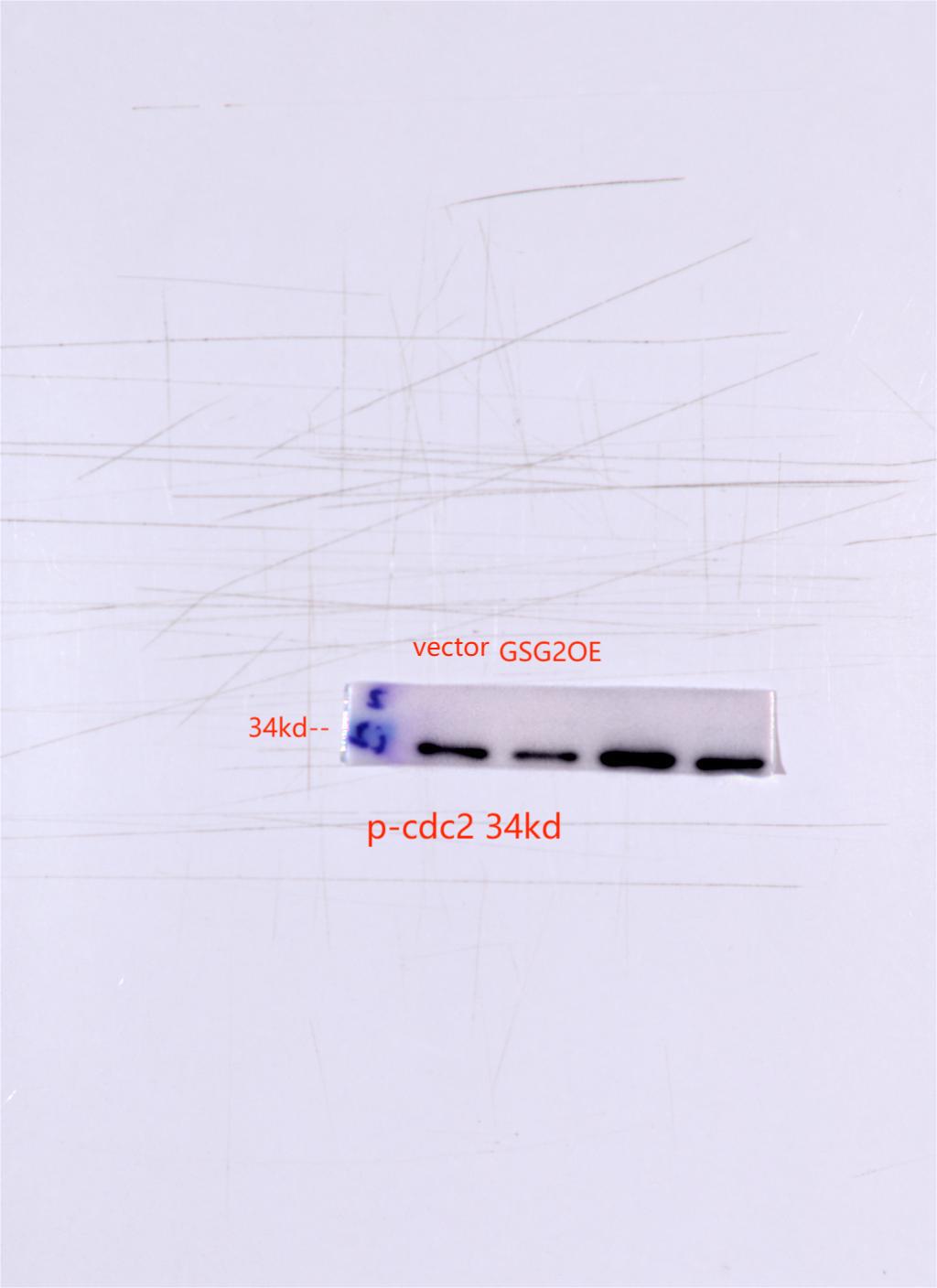

Supplement: Supplementary file 1 — Supplementary file1 (ZIP 11,586 kb) [file 10735_2024_10185_MOESM1_ESM.zip › 10735_2024_10185_MOESM1_ESM/Supplementary Material/Fig3B-HO8910-OE-Pcdc2.jpg]

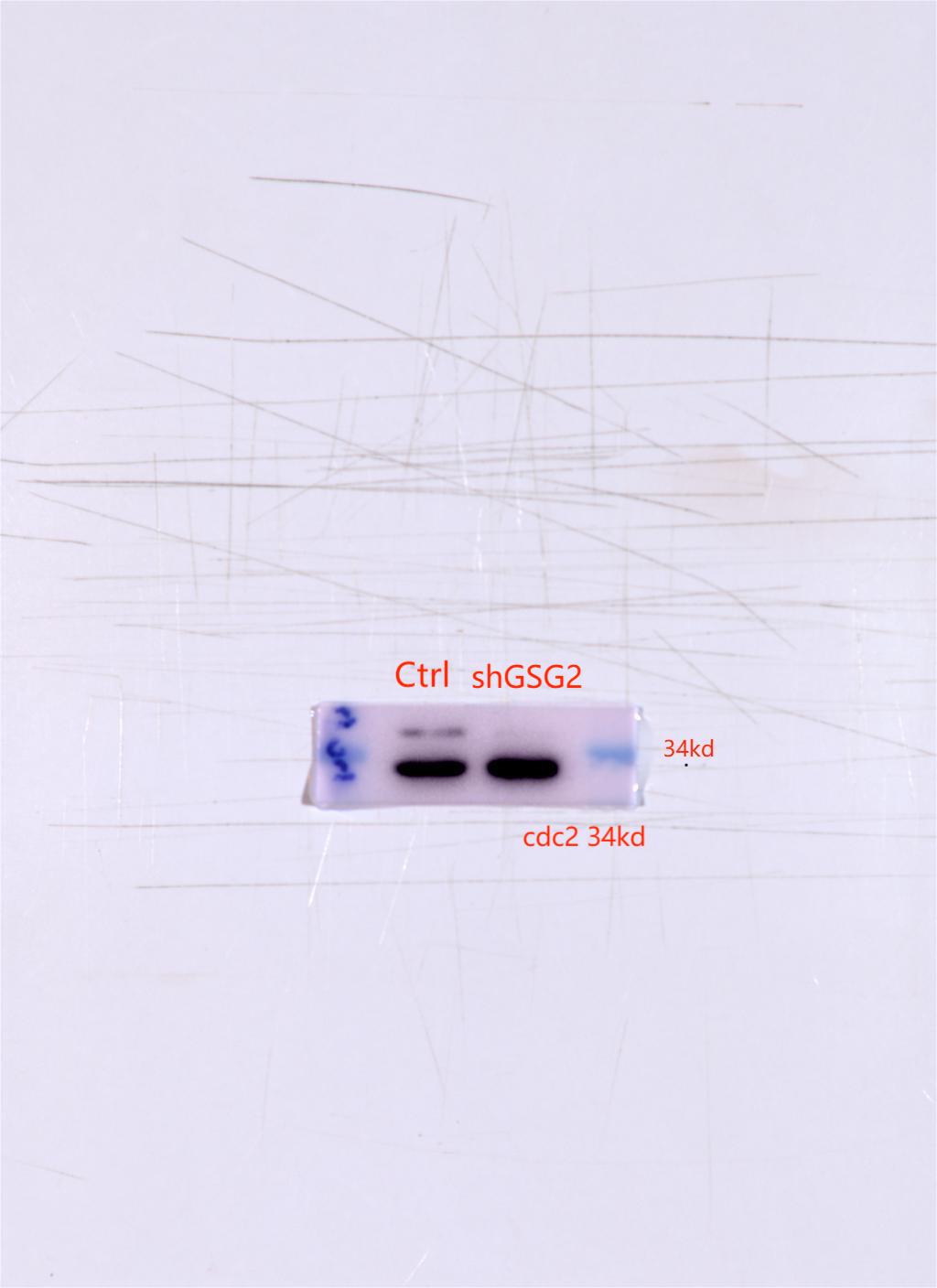

Supplement: Supplementary file 1 — Supplementary file1 (ZIP 11,586 kb) [file 10735_2024_10185_MOESM1_ESM.zip › 10735_2024_10185_MOESM1_ESM/Supplementary Material/Fig3B-HO8910-sh-cdc2.jpg]

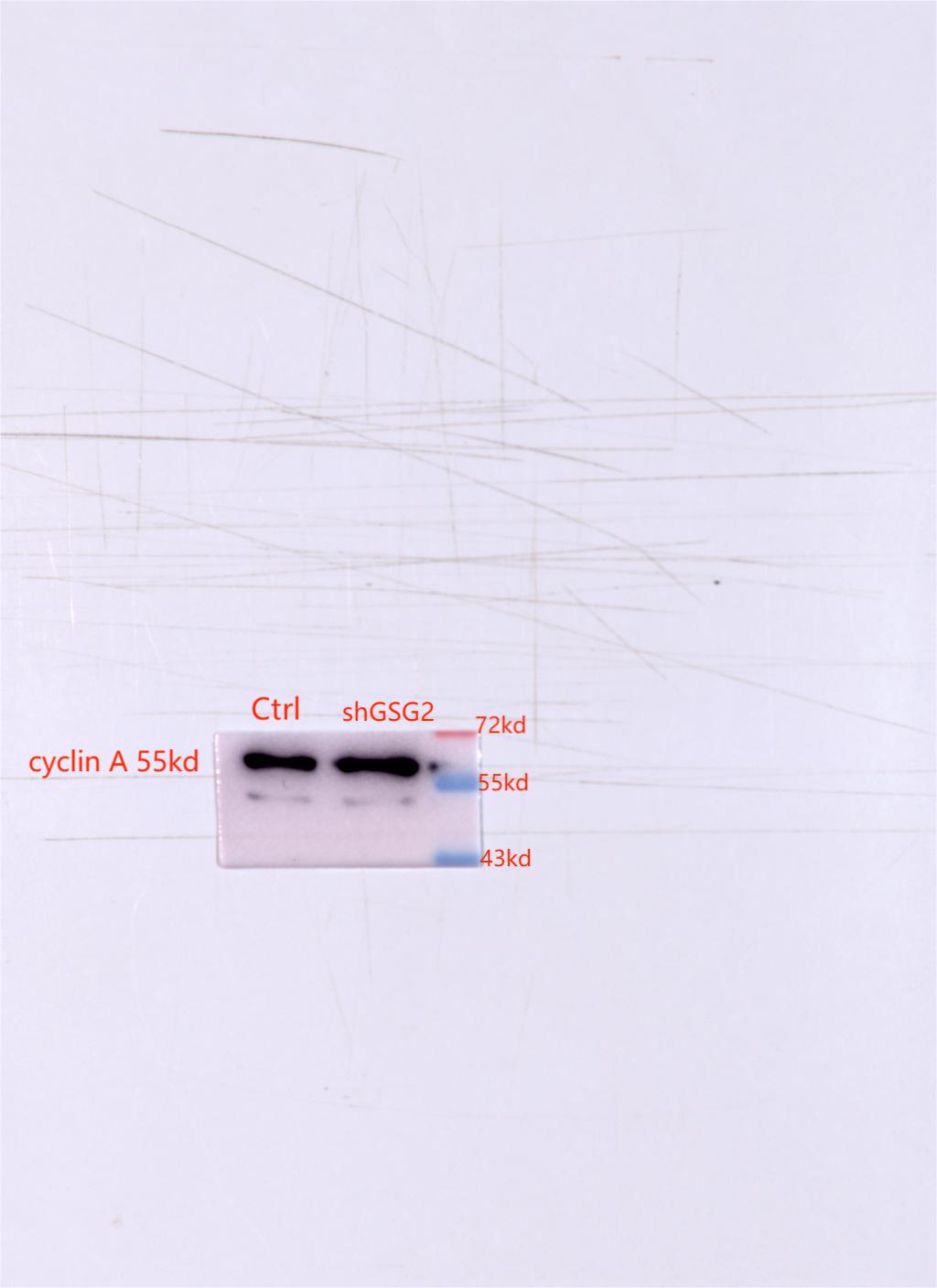

Supplement: Supplementary file 1 — Supplementary file1 (ZIP 11,586 kb) [file 10735_2024_10185_MOESM1_ESM.zip › 10735_2024_10185_MOESM1_ESM/Supplementary Material/Fig3B-HO8910-sh-cyclin A.jpg]

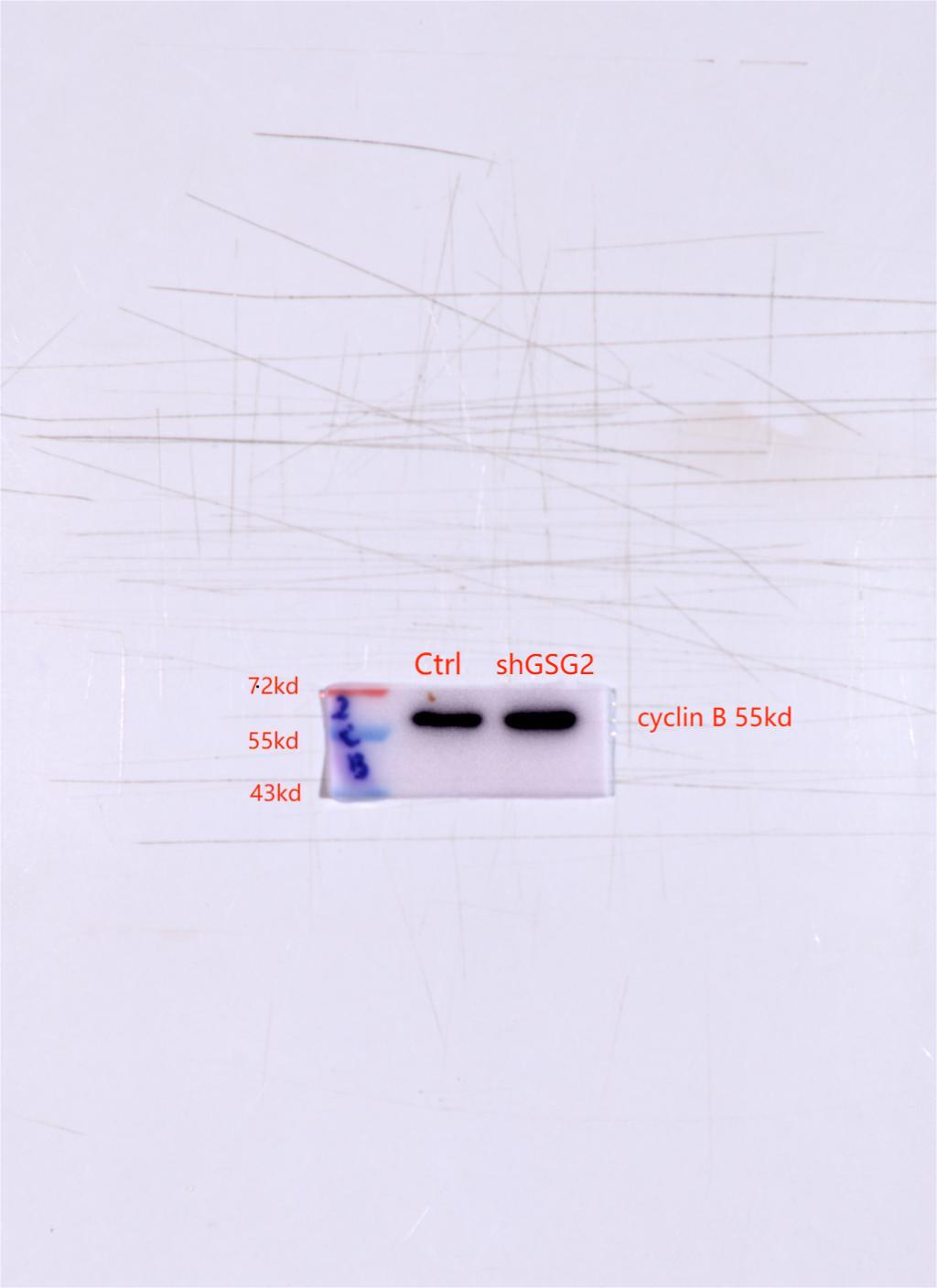

Supplement: Supplementary file 1 — Supplementary file1 (ZIP 11,586 kb) [file 10735_2024_10185_MOESM1_ESM.zip › 10735_2024_10185_MOESM1_ESM/Supplementary Material/Fig3B-HO8910-sh-cyclin B.jpg]

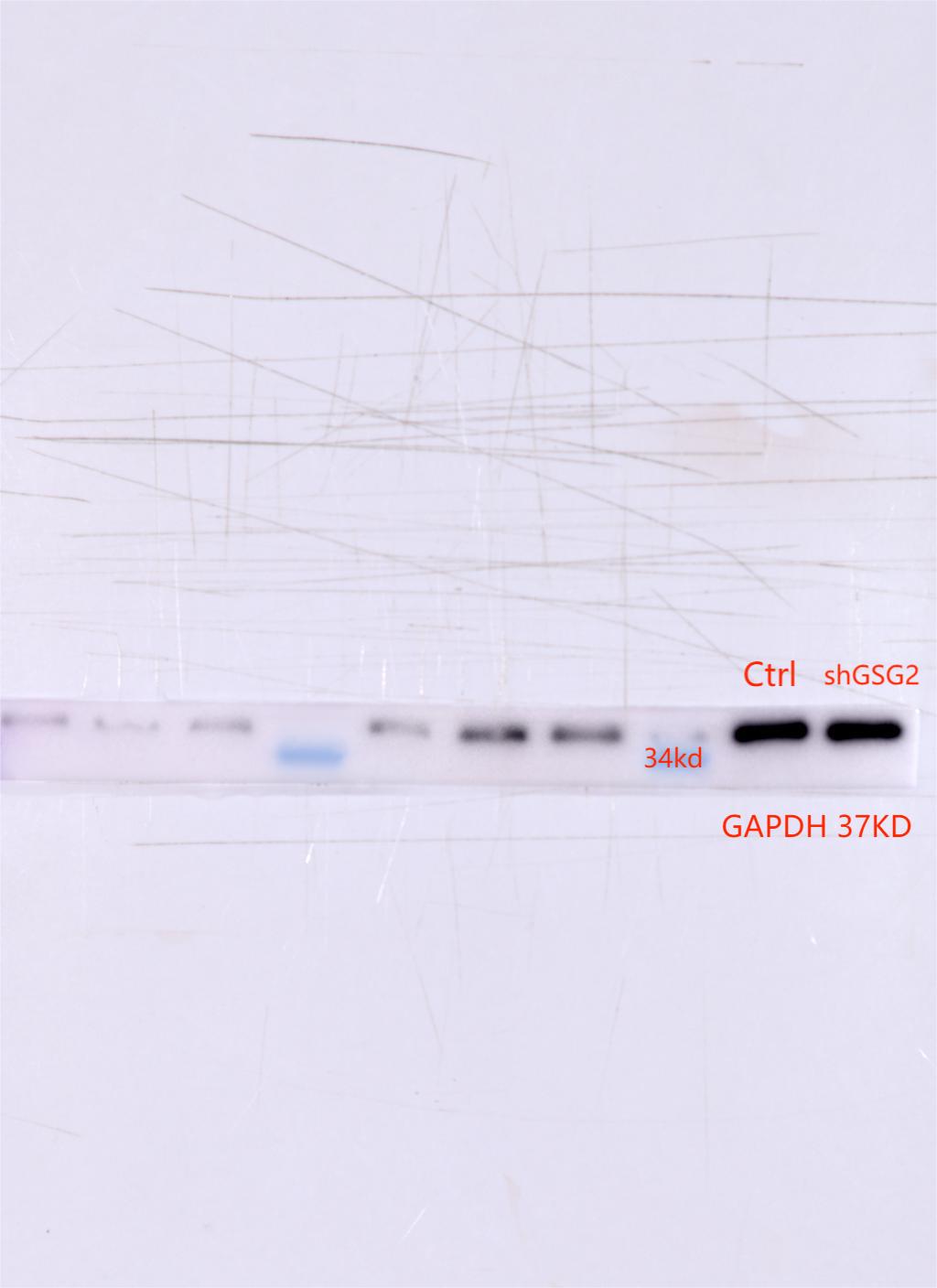

Supplement: Supplementary file 1 — Supplementary file1 (ZIP 11,586 kb) [file 10735_2024_10185_MOESM1_ESM.zip › 10735_2024_10185_MOESM1_ESM/Supplementary Material/Fig3B-HO8910-sh-GAPDH.jpg]

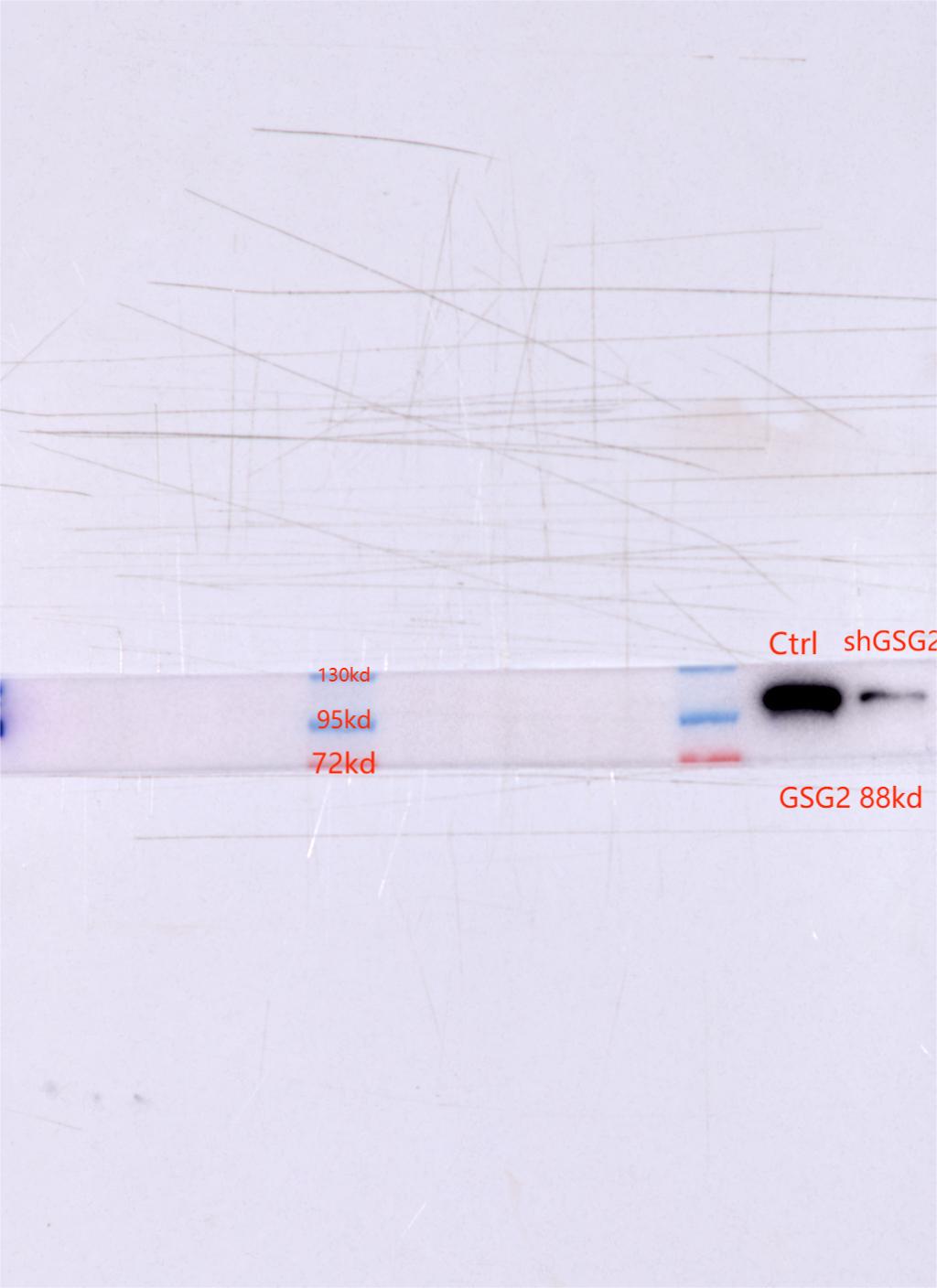

Supplement: Supplementary file 1 — Supplementary file1 (ZIP 11,586 kb) [file 10735_2024_10185_MOESM1_ESM.zip › 10735_2024_10185_MOESM1_ESM/Supplementary Material/Fig3B-HO8910-sh-GSG2.jpg]

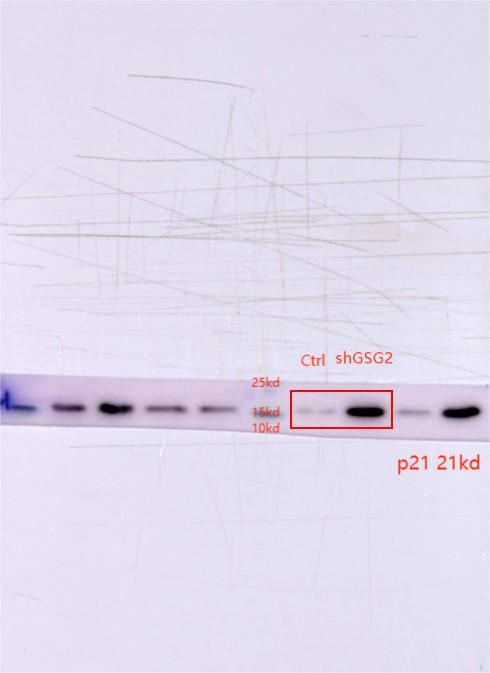

Supplement: Supplementary file 1 — Supplementary file1 (ZIP 11,586 kb) [file 10735_2024_10185_MOESM1_ESM.zip › 10735_2024_10185_MOESM1_ESM/Supplementary Material/Fig3B-HO8910-sh-p21.jpg]

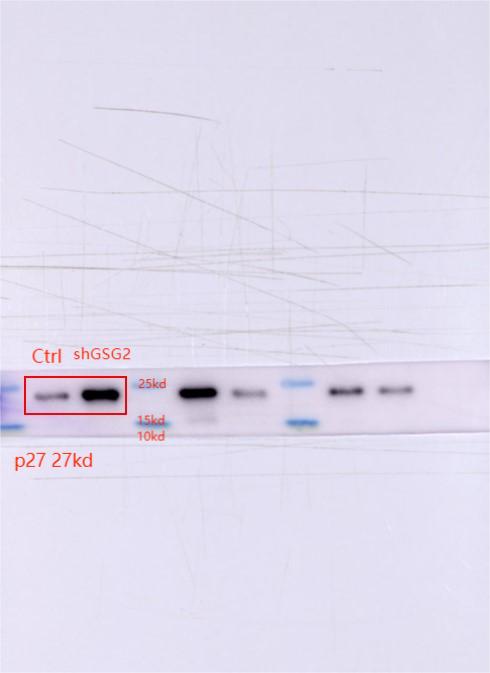

Supplement: Supplementary file 1 — Supplementary file1 (ZIP 11,586 kb) [file 10735_2024_10185_MOESM1_ESM.zip › 10735_2024_10185_MOESM1_ESM/Supplementary Material/Fig3B-HO8910-sh-p27.jpg]

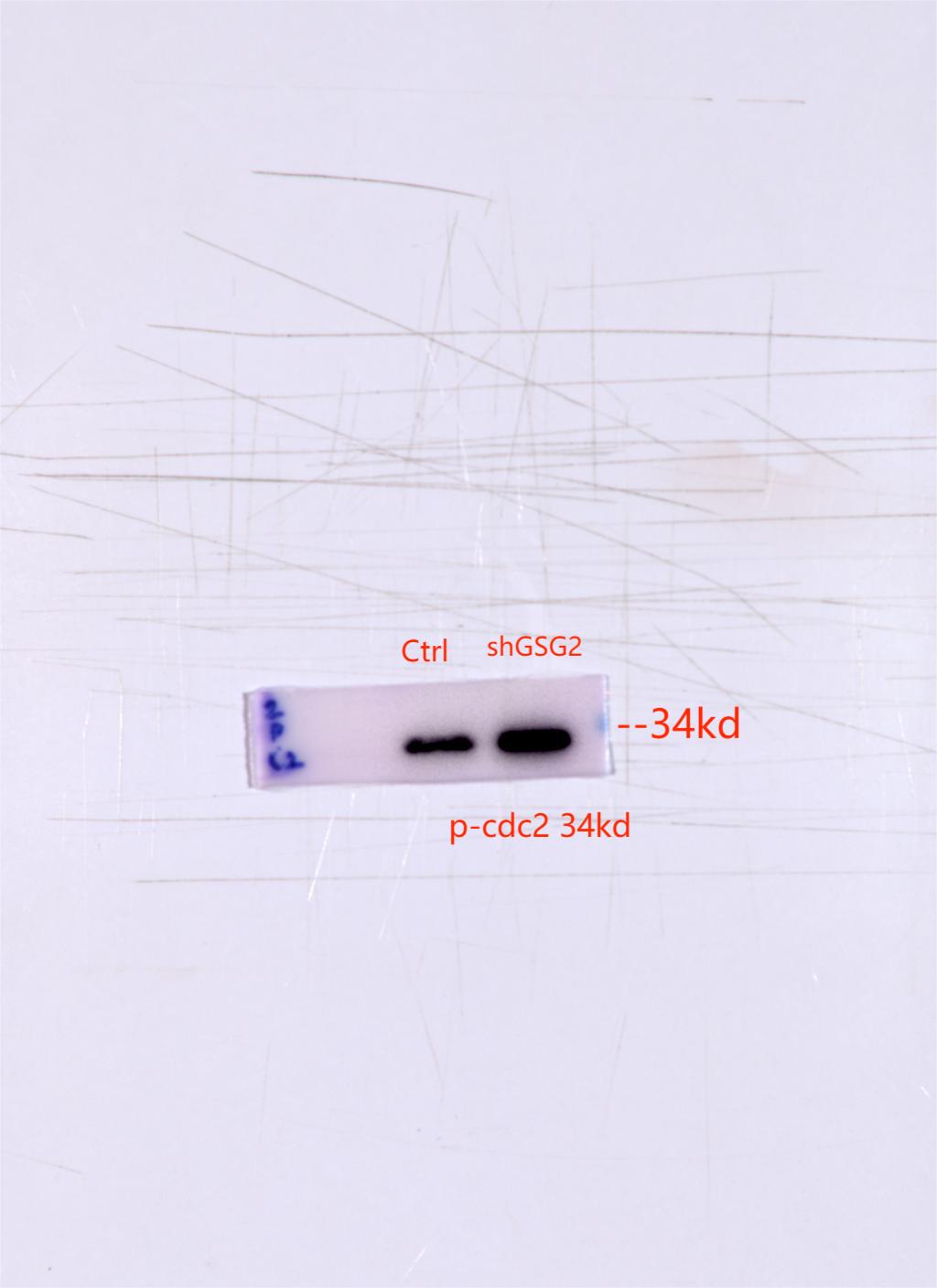

Supplement: Supplementary file 1 — Supplementary file1 (ZIP 11,586 kb) [file 10735_2024_10185_MOESM1_ESM.zip › 10735_2024_10185_MOESM1_ESM/Supplementary Material/Fig3B-HO8910-sh-pcdc2.jpg]

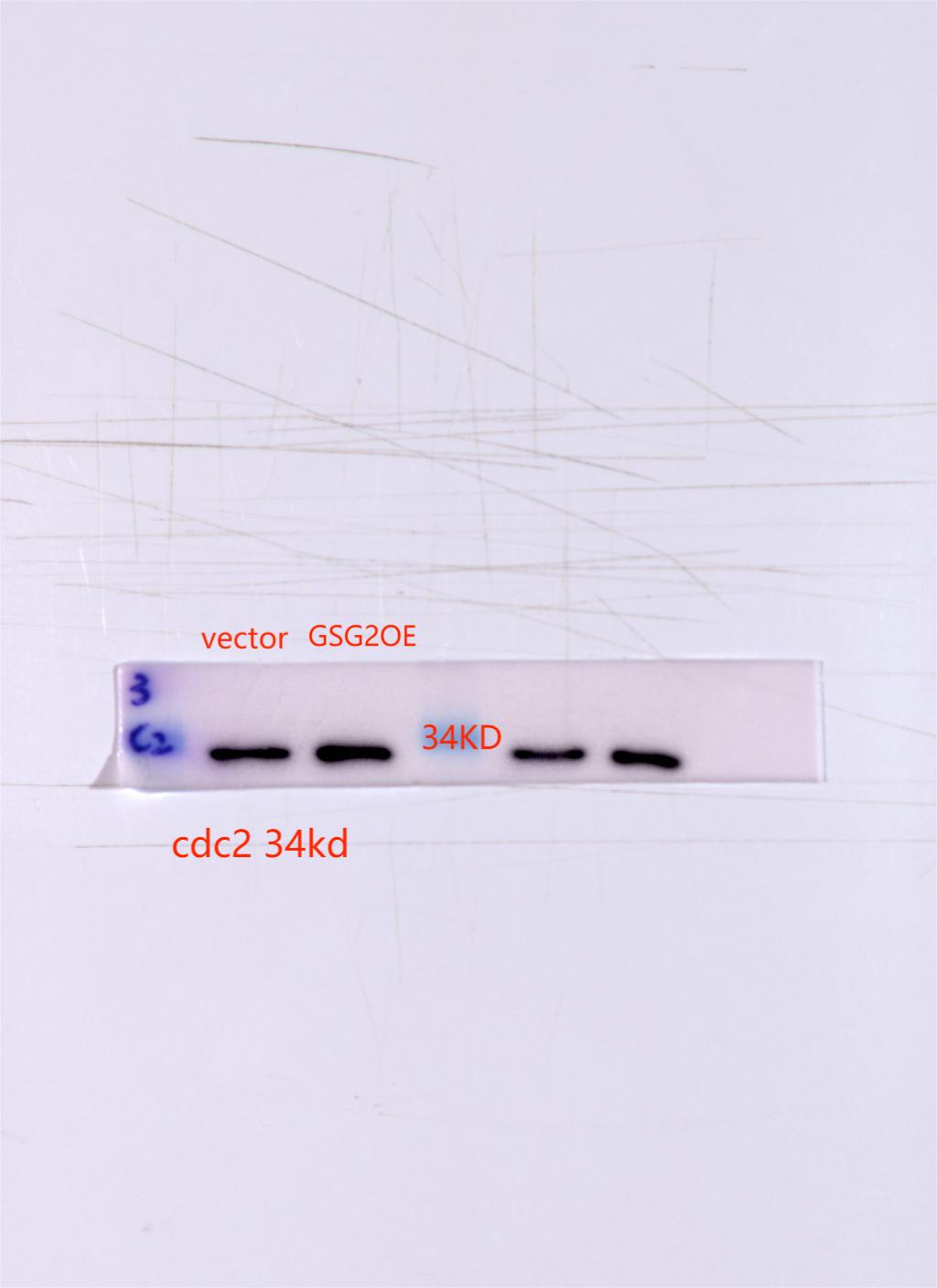

Supplement: Supplementary file 1 — Supplementary file1 (ZIP 11,586 kb) [file 10735_2024_10185_MOESM1_ESM.zip › 10735_2024_10185_MOESM1_ESM/Supplementary Material/Fig3B-SKOV3-OE-cdc2.jpg]

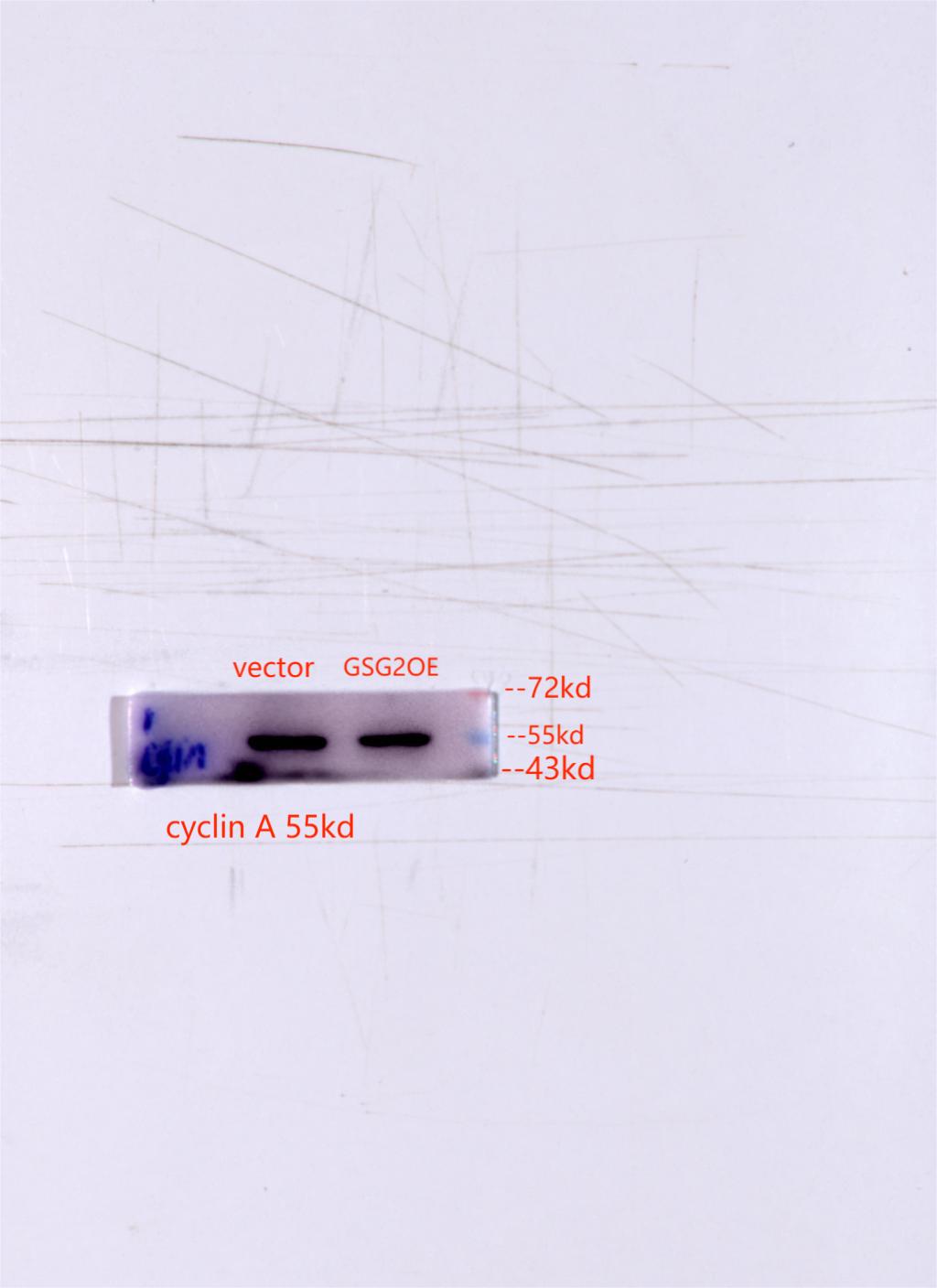

Supplement: Supplementary file 1 — Supplementary file1 (ZIP 11,586 kb) [file 10735_2024_10185_MOESM1_ESM.zip › 10735_2024_10185_MOESM1_ESM/Supplementary Material/Fig3B-SKOV3-OE-cyclin A .jpg]

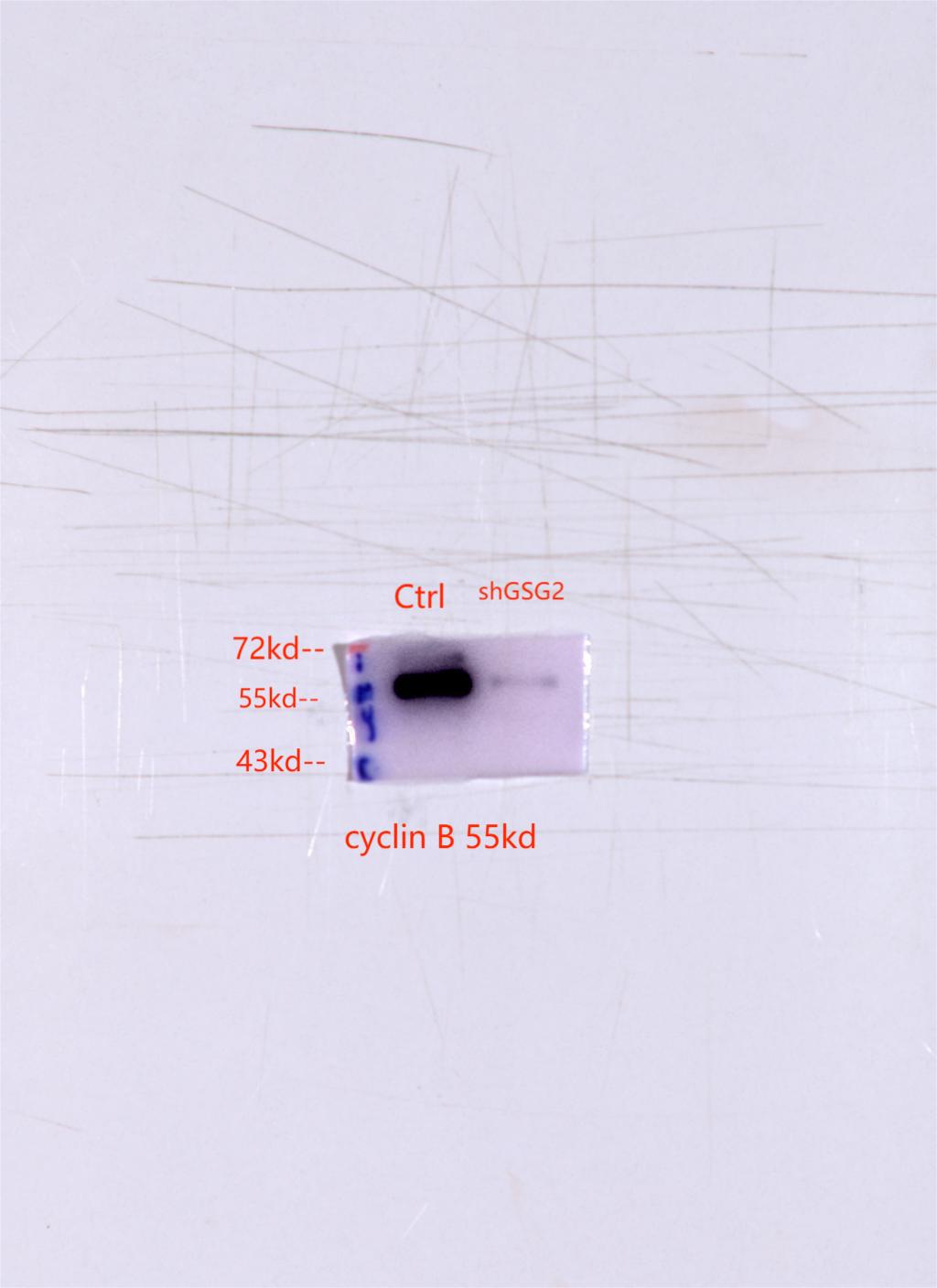

Supplement: Supplementary file 1 — Supplementary file1 (ZIP 11,586 kb) [file 10735_2024_10185_MOESM1_ESM.zip › 10735_2024_10185_MOESM1_ESM/Supplementary Material/Fig3B-SKOV3-OE-cyclin B.jpg]

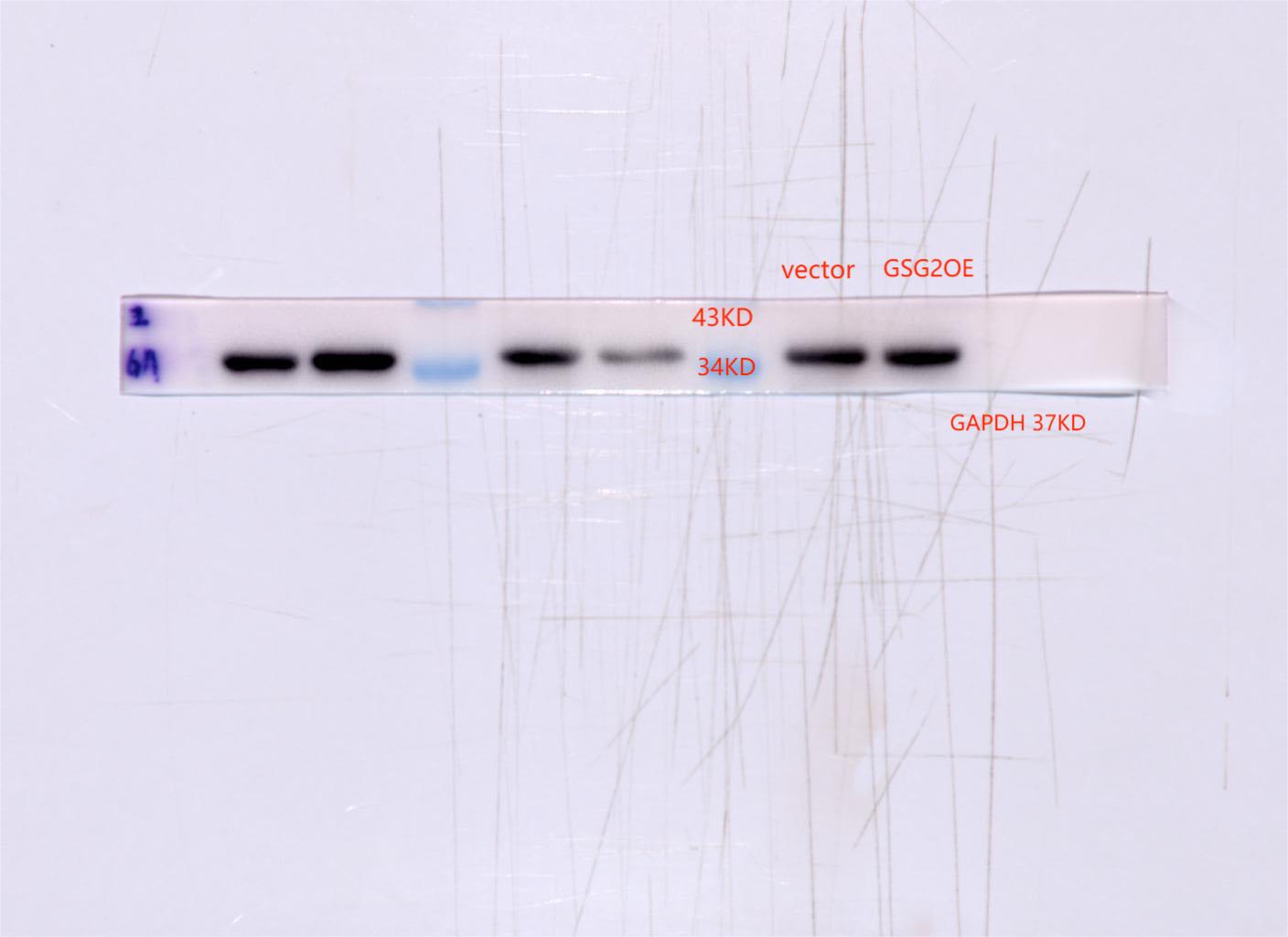

Supplement: Supplementary file 1 — Supplementary file1 (ZIP 11,586 kb) [file 10735_2024_10185_MOESM1_ESM.zip › 10735_2024_10185_MOESM1_ESM/Supplementary Material/Fig3B-SKOV3-OE-GAPDH.jpg]

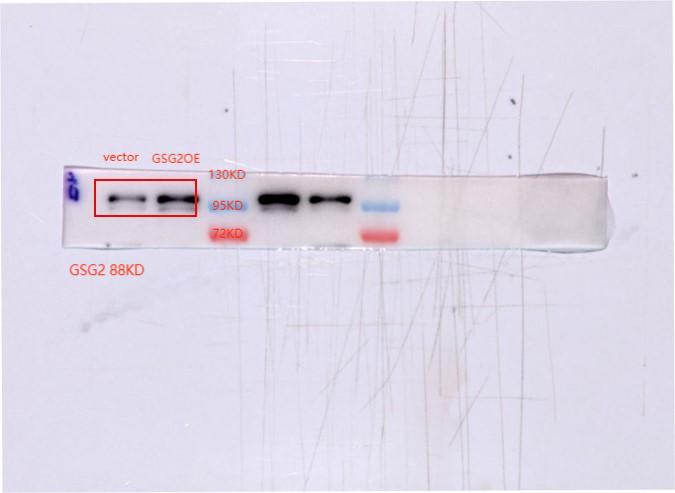

Supplement: Supplementary file 1 — Supplementary file1 (ZIP 11,586 kb) [file 10735_2024_10185_MOESM1_ESM.zip › 10735_2024_10185_MOESM1_ESM/Supplementary Material/Fig3B-SKOV3-OE-GSG2.jpg]

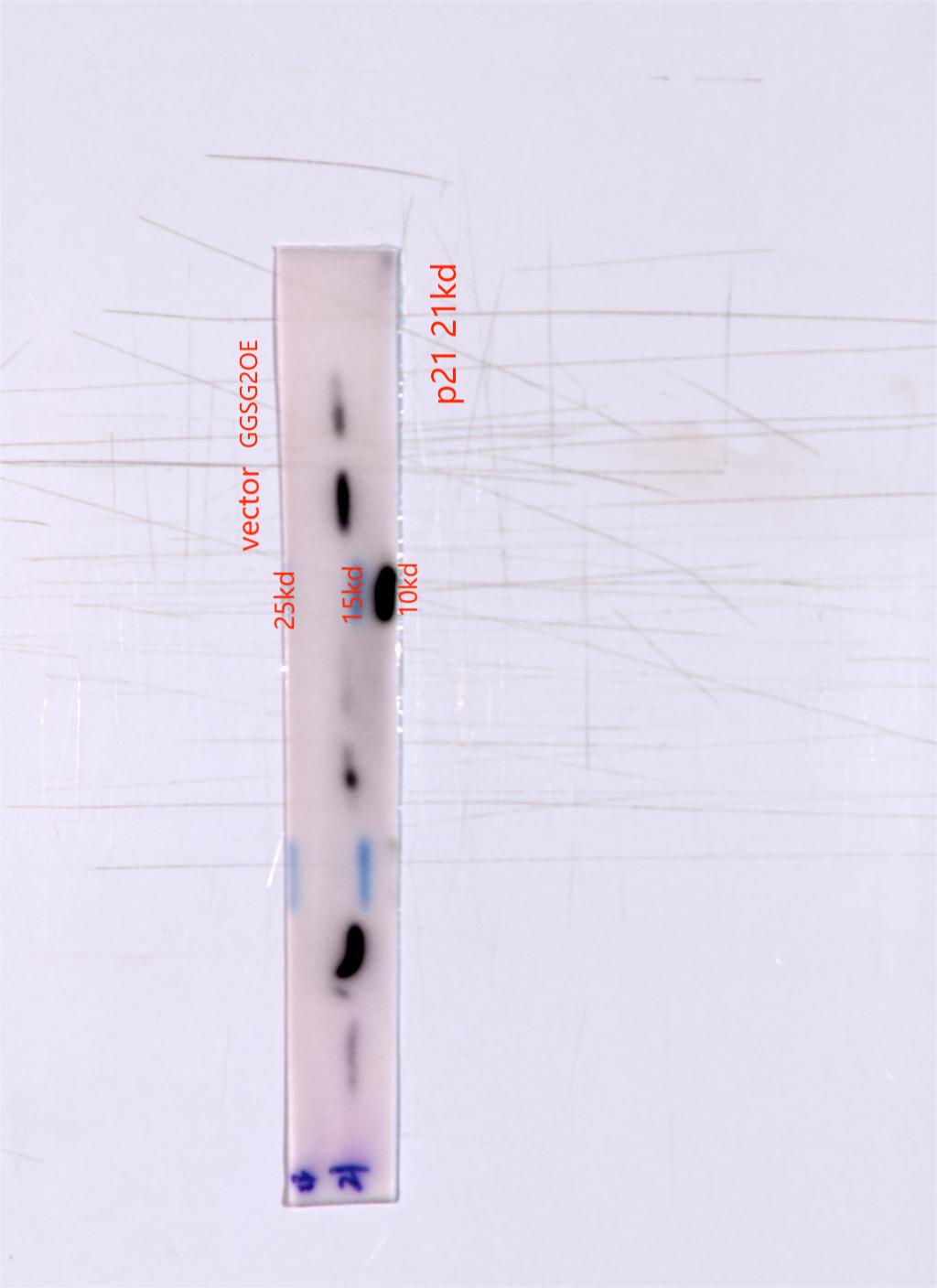

Supplement: Supplementary file 1 — Supplementary file1 (ZIP 11,586 kb) [file 10735_2024_10185_MOESM1_ESM.zip › 10735_2024_10185_MOESM1_ESM/Supplementary Material/Fig3B-SKOV3-OE-p21.jpg]

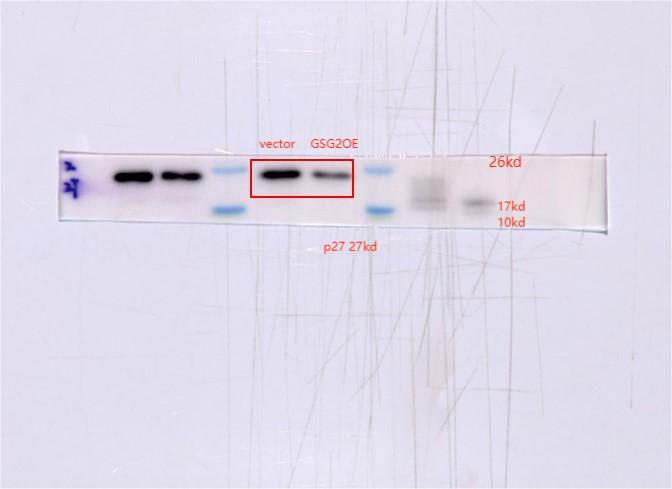

Supplement: Supplementary file 1 — Supplementary file1 (ZIP 11,586 kb) [file 10735_2024_10185_MOESM1_ESM.zip › 10735_2024_10185_MOESM1_ESM/Supplementary Material/Fig3B-SKOV3-OE-p27.jpg]

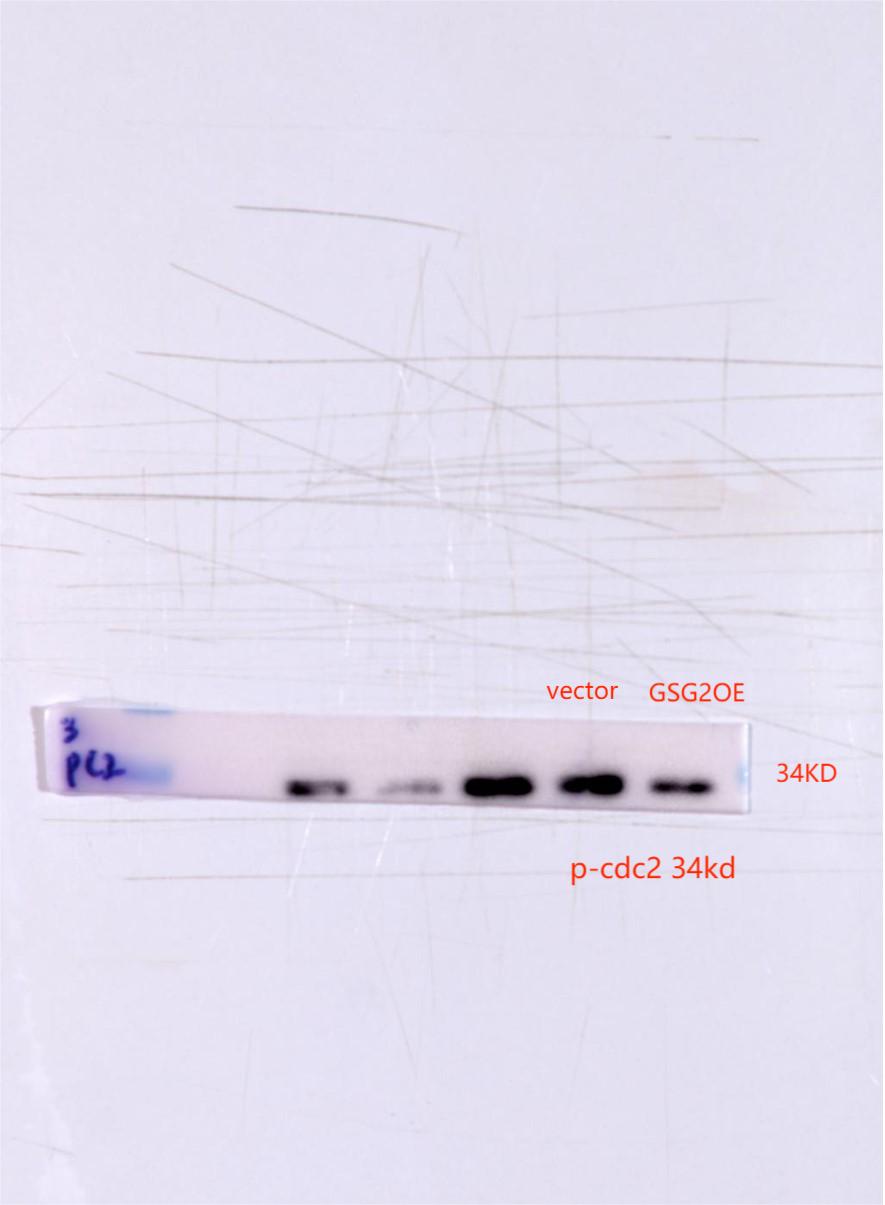

Supplement: Supplementary file 1 — Supplementary file1 (ZIP 11,586 kb) [file 10735_2024_10185_MOESM1_ESM.zip › 10735_2024_10185_MOESM1_ESM/Supplementary Material/Fig3B-SKOV3-OE-pcdc2.jpg]

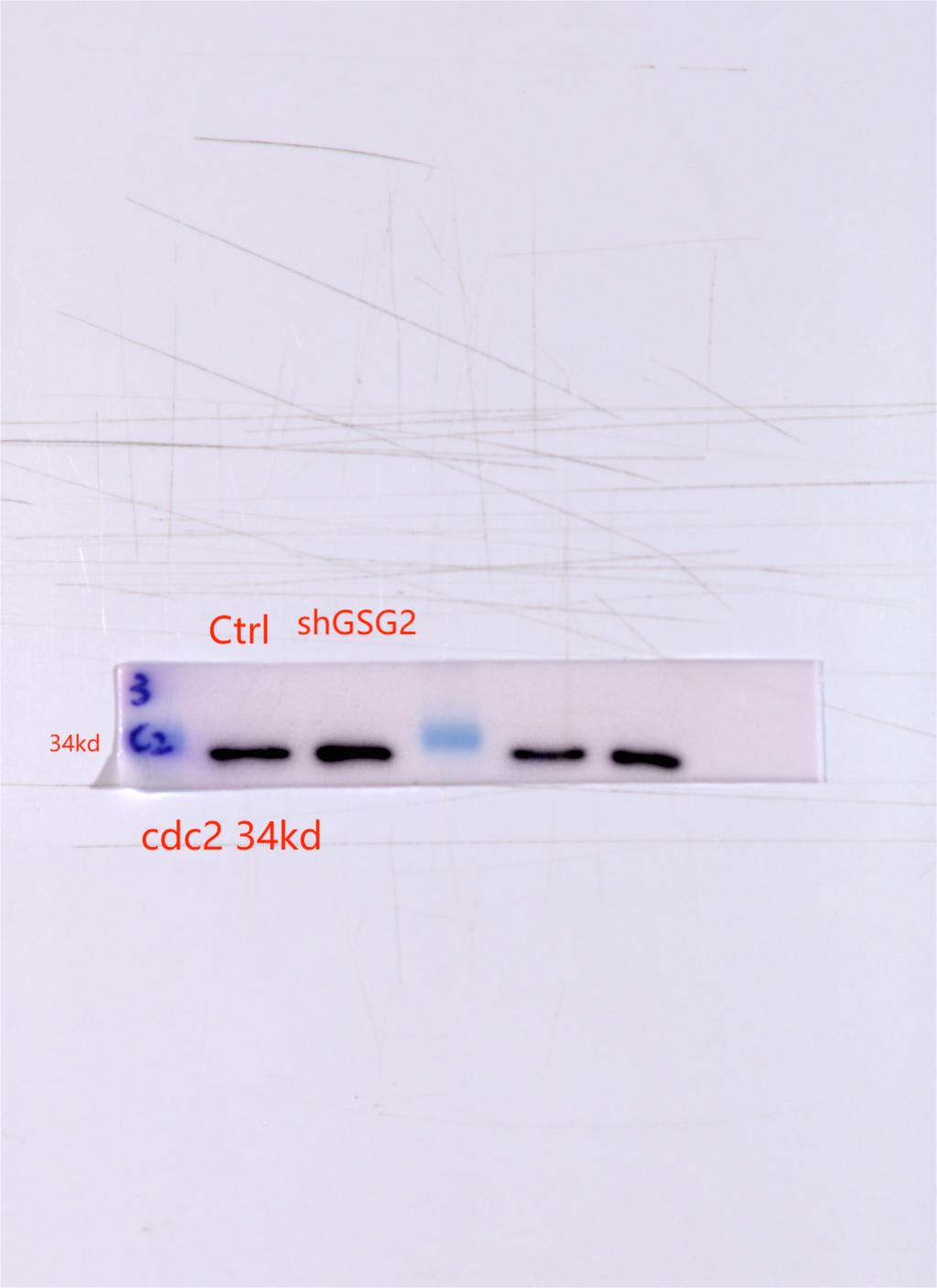

Supplement: Supplementary file 1 — Supplementary file1 (ZIP 11,586 kb) [file 10735_2024_10185_MOESM1_ESM.zip › 10735_2024_10185_MOESM1_ESM/Supplementary Material/Fig3B-SKOV3-sh-cdc2.jpg]

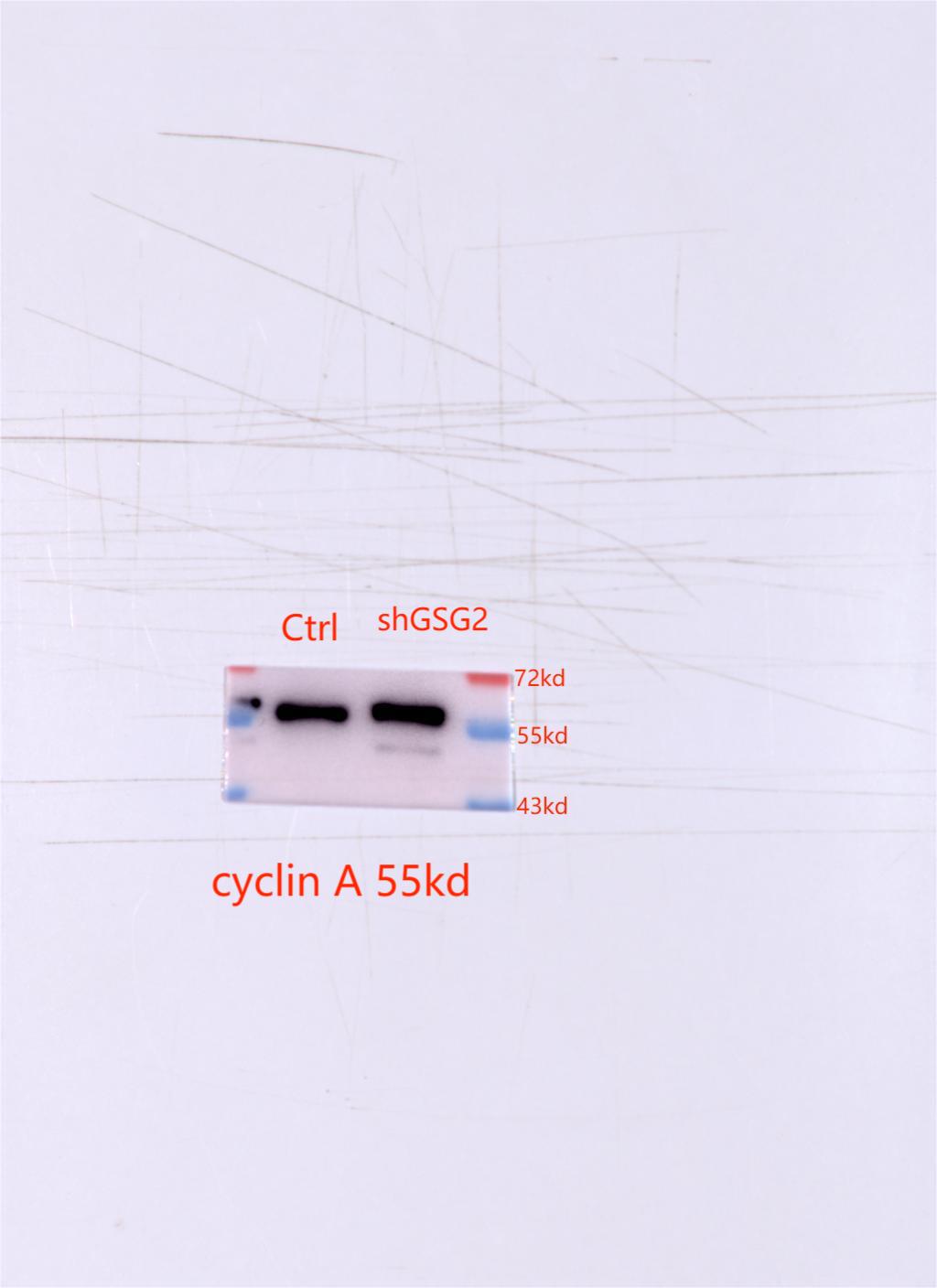

Supplement: Supplementary file 1 — Supplementary file1 (ZIP 11,586 kb) [file 10735_2024_10185_MOESM1_ESM.zip › 10735_2024_10185_MOESM1_ESM/Supplementary Material/Fig3B-SKOV3-sh-cyclin A.jpg]

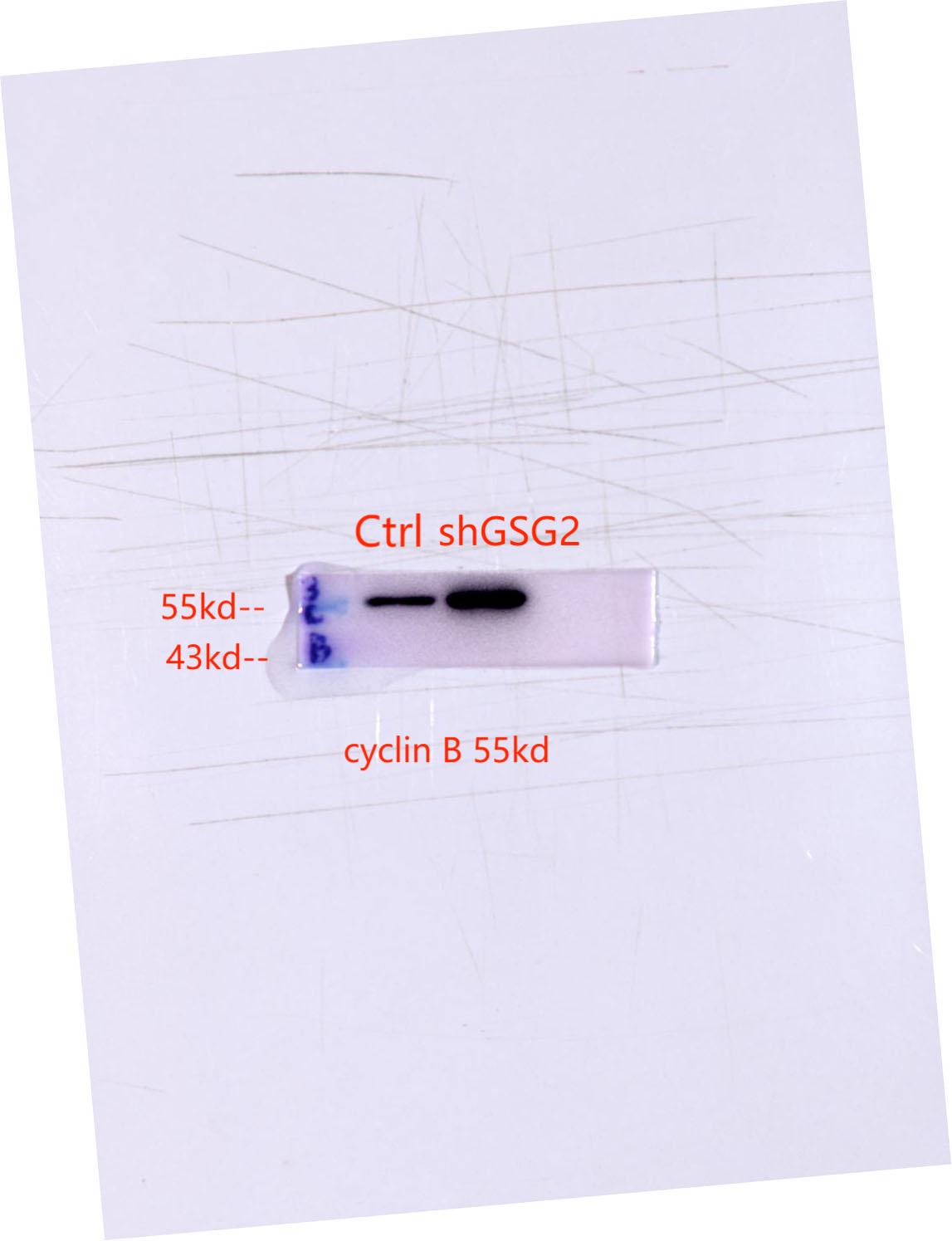

Supplement: Supplementary file 1 — Supplementary file1 (ZIP 11,586 kb) [file 10735_2024_10185_MOESM1_ESM.zip › 10735_2024_10185_MOESM1_ESM/Supplementary Material/Fig3B-SKOV3-sh-CyclinB.jpg]

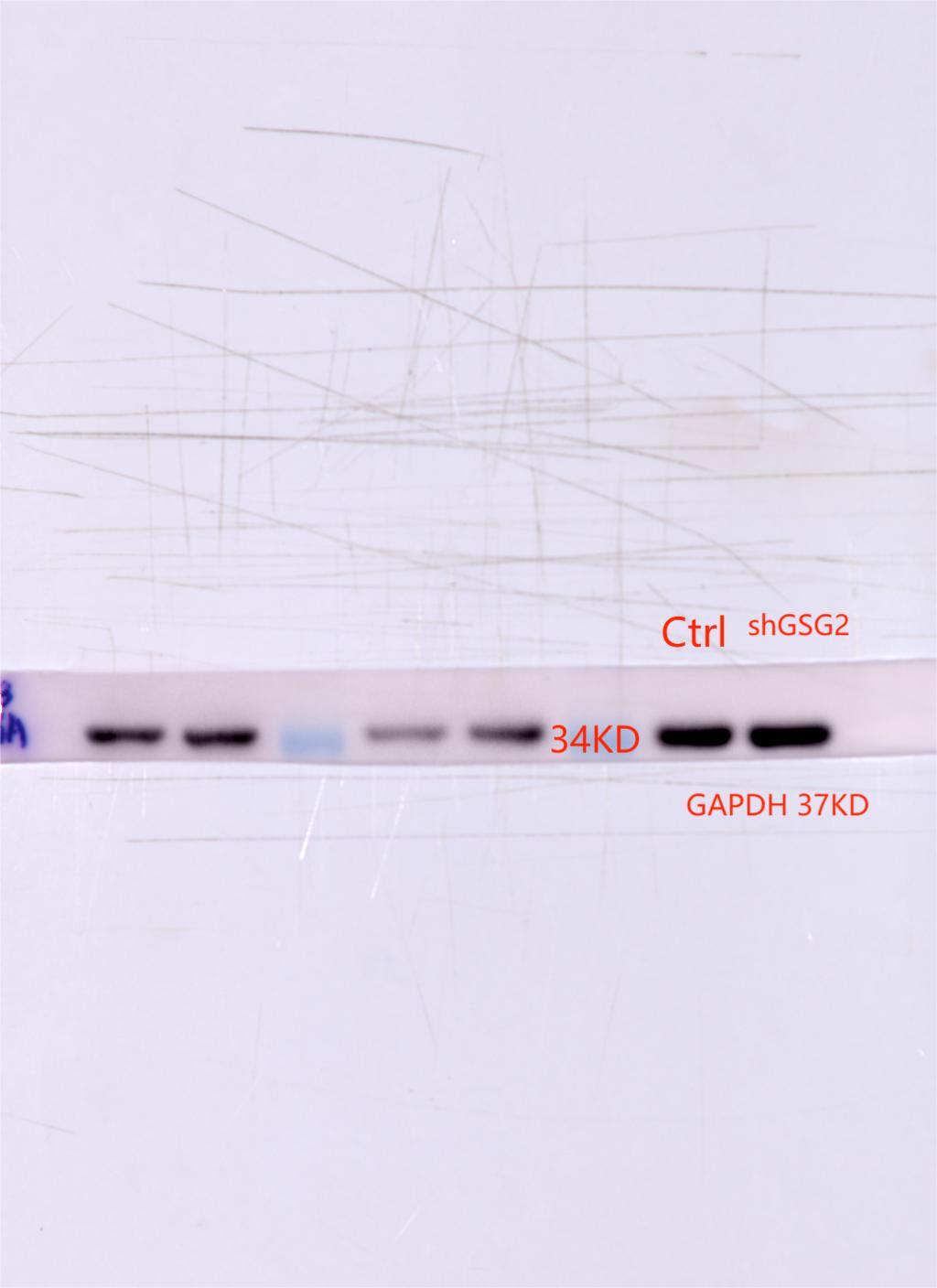

Supplement: Supplementary file 1 — Supplementary file1 (ZIP 11,586 kb) [file 10735_2024_10185_MOESM1_ESM.zip › 10735_2024_10185_MOESM1_ESM/Supplementary Material/Fig3B-SKOV3-sh-GAPDH.jpg]

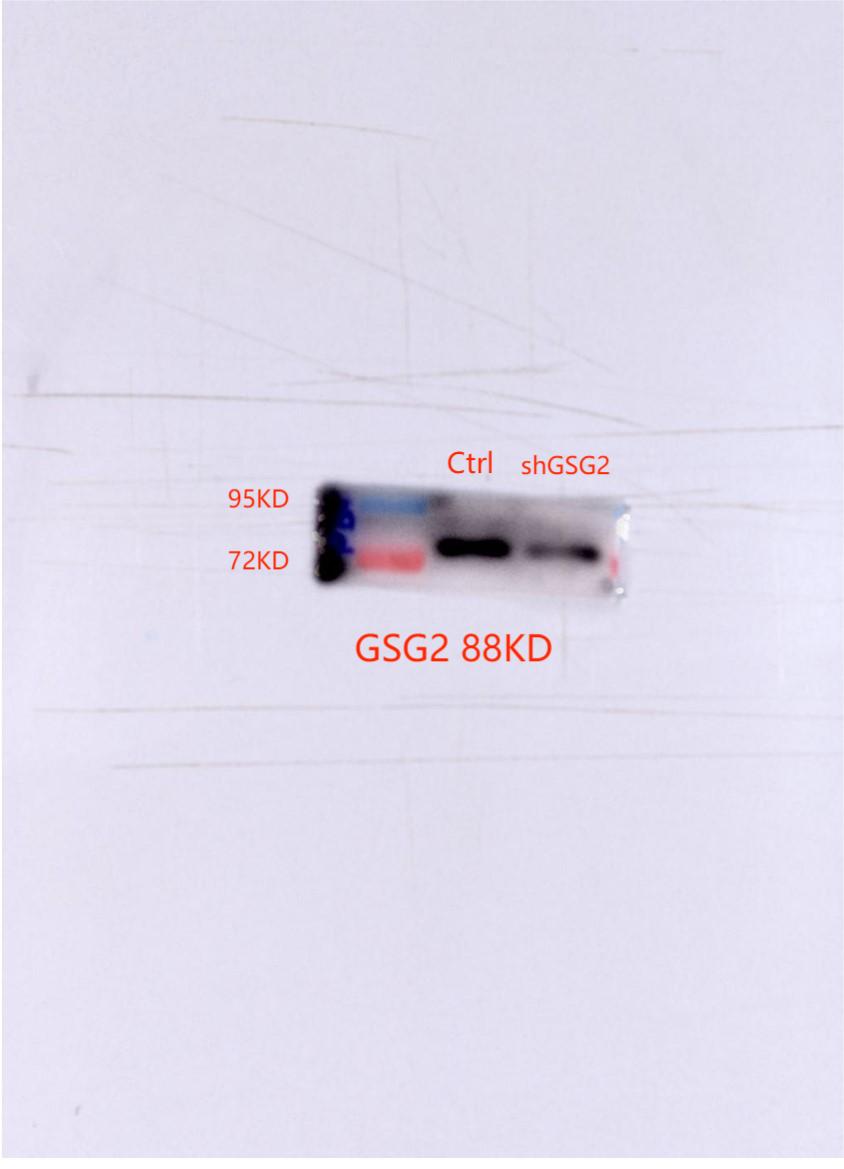

Supplement: Supplementary file 1 — Supplementary file1 (ZIP 11,586 kb) [file 10735_2024_10185_MOESM1_ESM.zip › 10735_2024_10185_MOESM1_ESM/Supplementary Material/Fig3B-SKOV3-sh-GSG2.jpg]

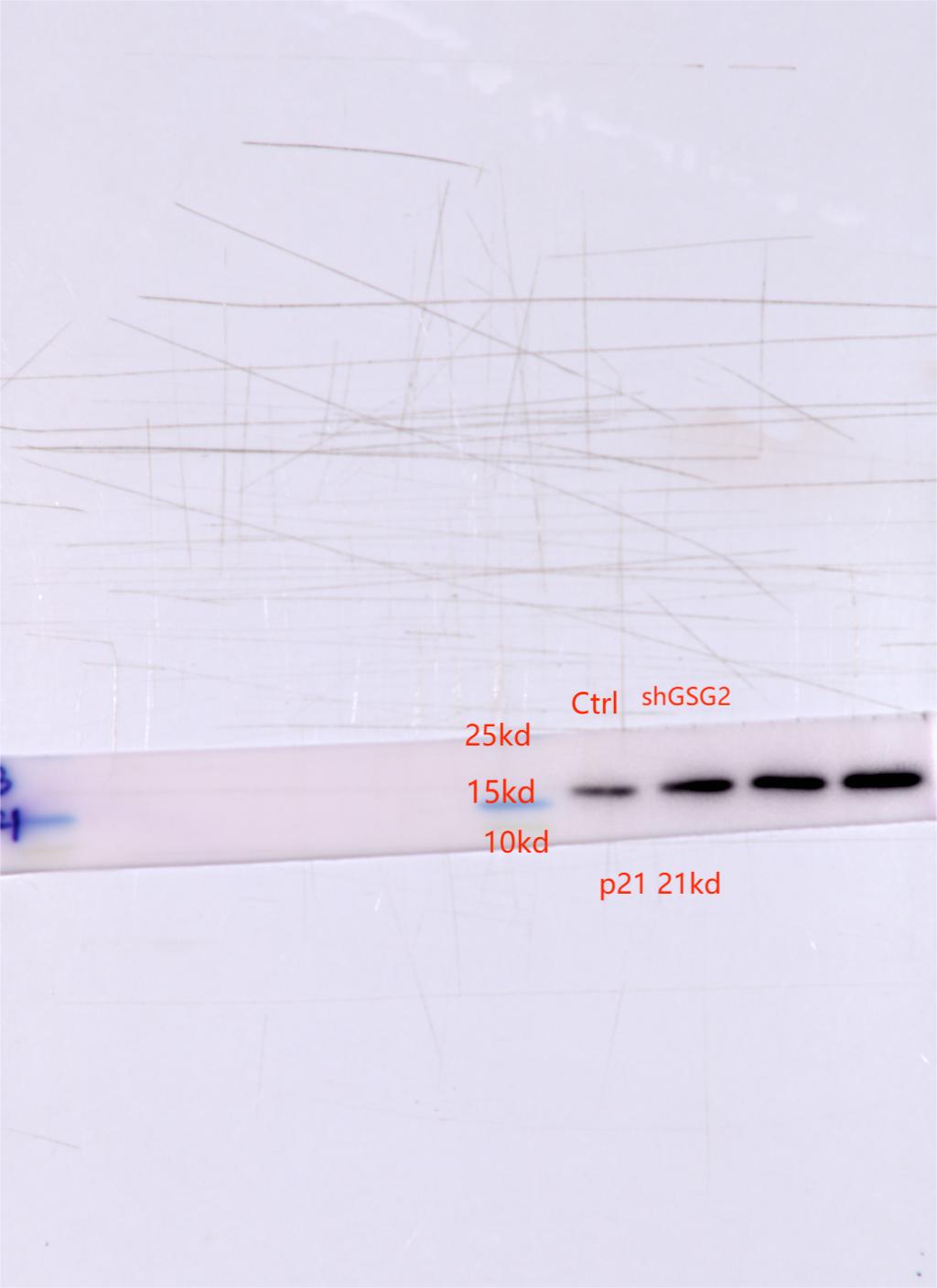

Supplement: Supplementary file 1 — Supplementary file1 (ZIP 11,586 kb) [file 10735_2024_10185_MOESM1_ESM.zip › 10735_2024_10185_MOESM1_ESM/Supplementary Material/Fig3B-SKOV3-sh-p21.jpg]

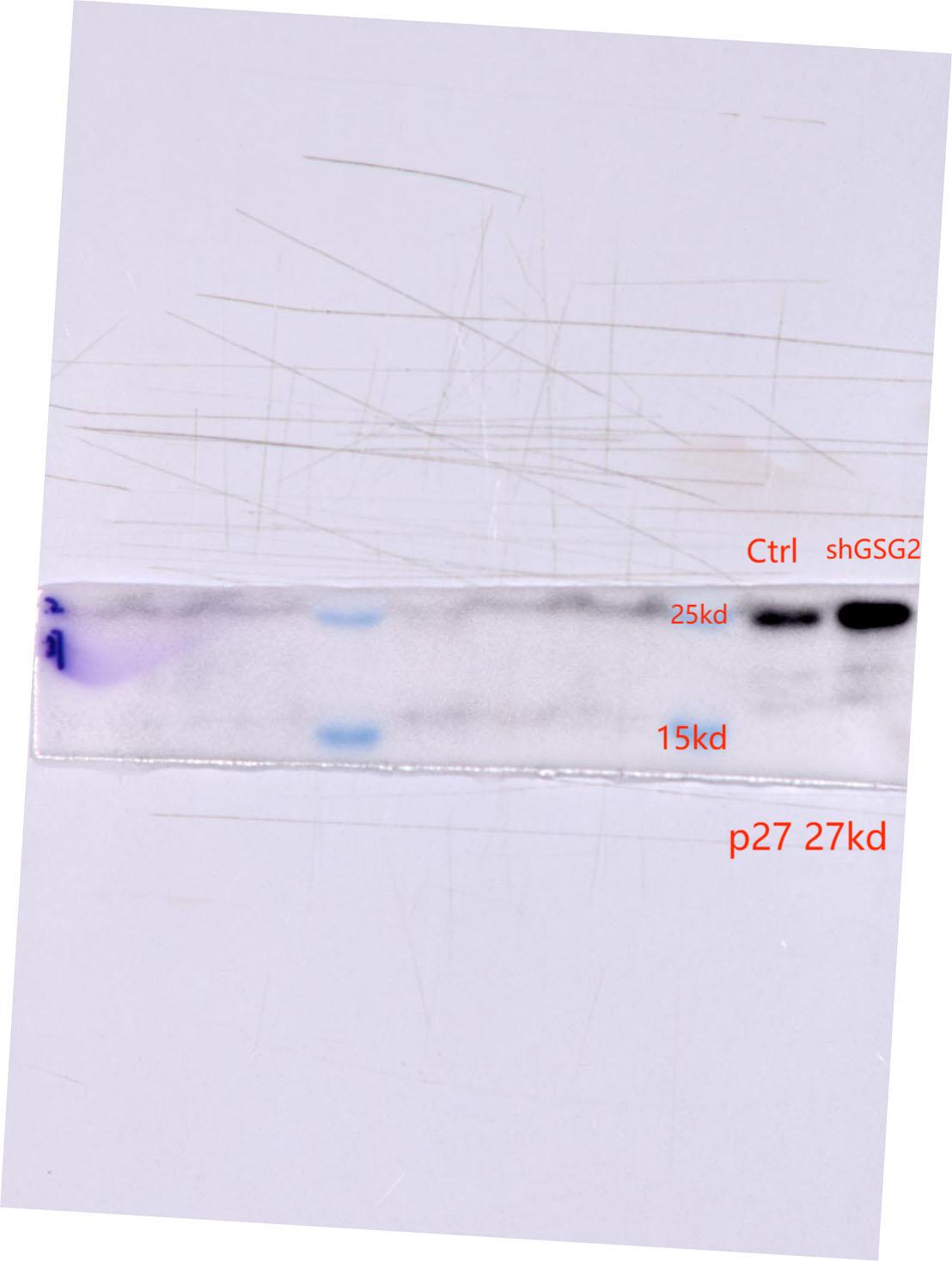

Supplement: Supplementary file 1 — Supplementary file1 (ZIP 11,586 kb) [file 10735_2024_10185_MOESM1_ESM.zip › 10735_2024_10185_MOESM1_ESM/Supplementary Material/Fig3B-SKOV3-sh-p27.jpg]

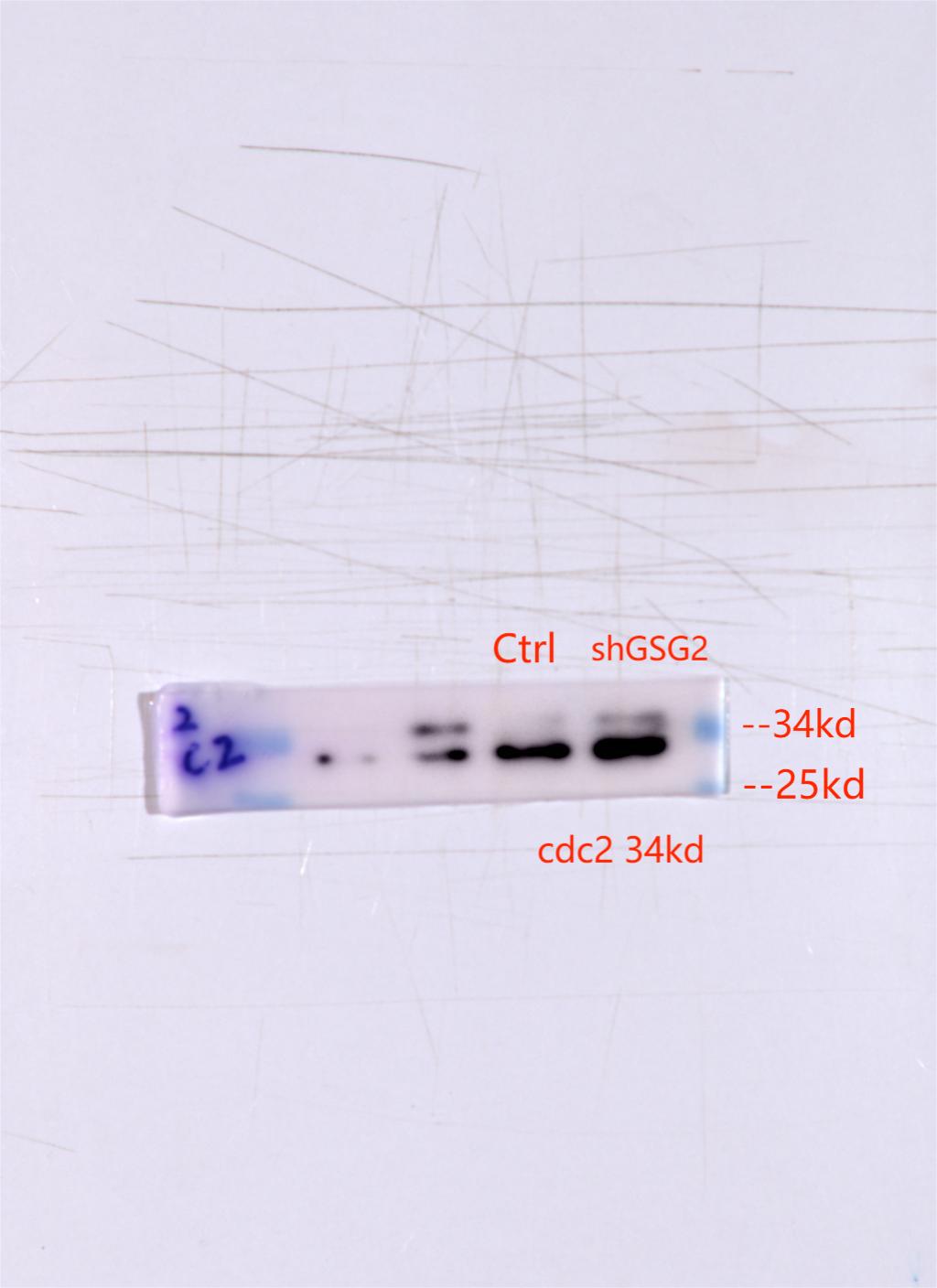

Supplement: Supplementary file 1 — Supplementary file1 (ZIP 11,586 kb) [file 10735_2024_10185_MOESM1_ESM.zip › 10735_2024_10185_MOESM1_ESM/Supplementary Material/Fig3B-SKOV3-sh-pcdc2.jpg]

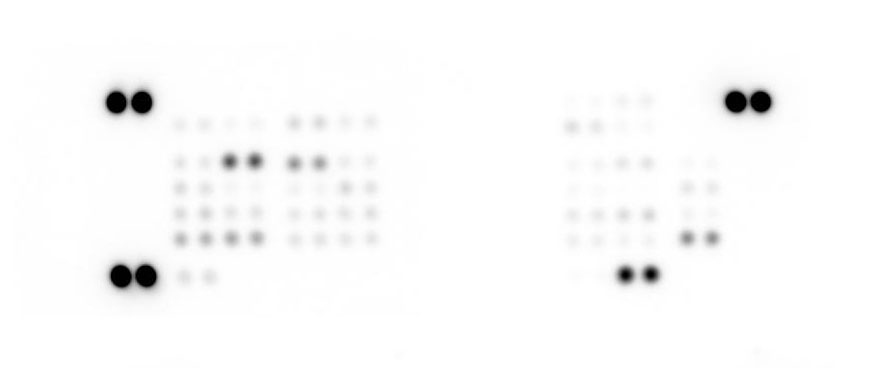

Supplement: Supplementary file 1 — Supplementary file1 (ZIP 11,586 kb) [file 10735_2024_10185_MOESM1_ESM.zip › 10735_2024_10185_MOESM1_ESM/Supplementary Material/Fig4A-Nc .jpg]

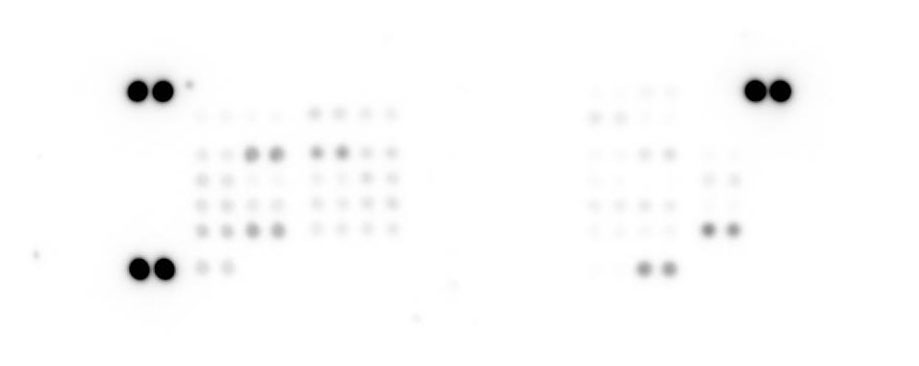

Supplement: Supplementary file 1 — Supplementary file1 (ZIP 11,586 kb) [file 10735_2024_10185_MOESM1_ESM.zip › 10735_2024_10185_MOESM1_ESM/Supplementary Material/Fig4A-sh.jpg]

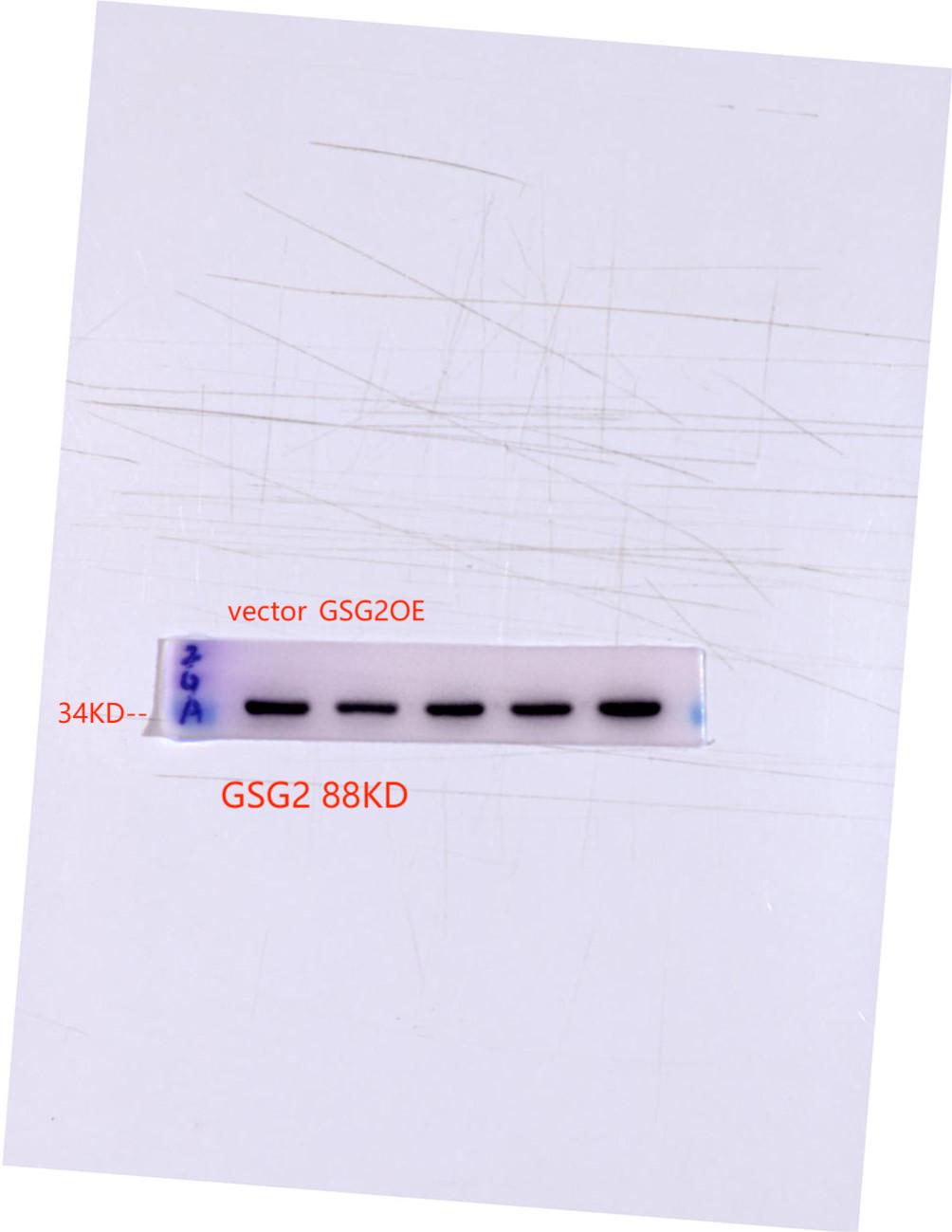

Supplement: Supplementary file 1 — Supplementary file1 (ZIP 11,586 kb) [file 10735_2024_10185_MOESM1_ESM.zip › 10735_2024_10185_MOESM1_ESM/Supplementary Material/Fig4B-HO8910-OE-GAPDH.jpg]

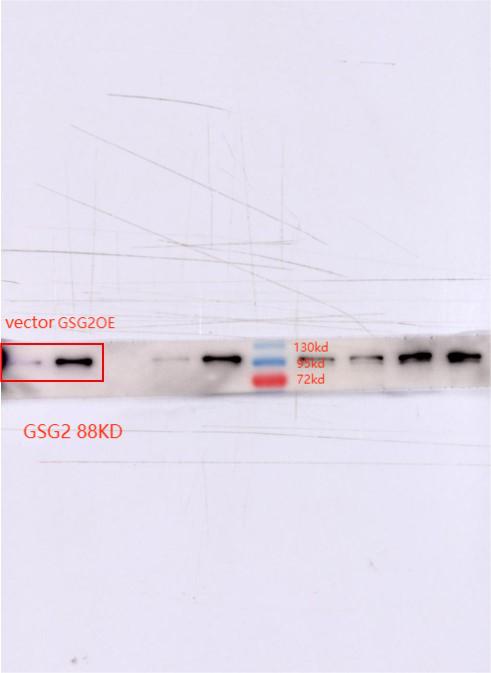

Supplement: Supplementary file 1 — Supplementary file1 (ZIP 11,586 kb) [file 10735_2024_10185_MOESM1_ESM.zip › 10735_2024_10185_MOESM1_ESM/Supplementary Material/Fig4B-HO8910-OE-GSG2.jpg]

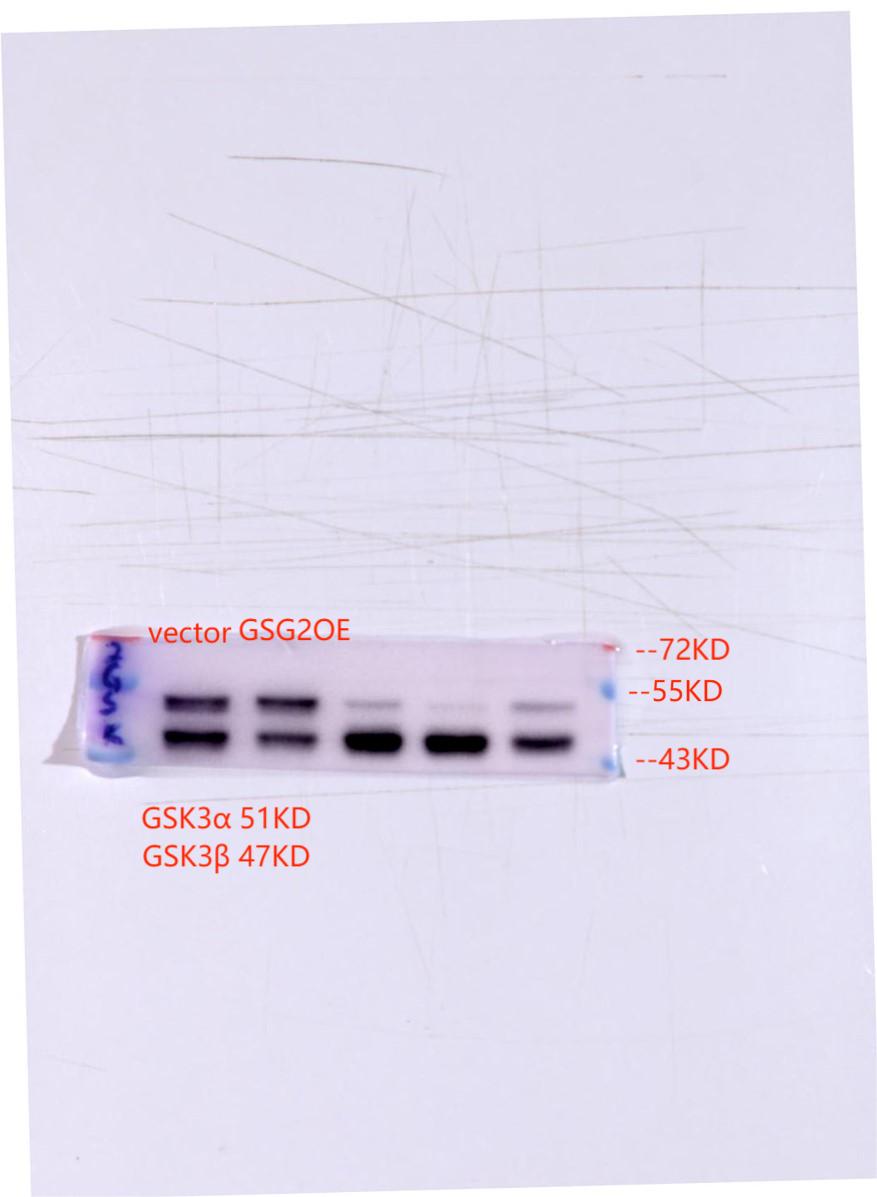

Supplement: Supplementary file 1 — Supplementary file1 (ZIP 11,586 kb) [file 10735_2024_10185_MOESM1_ESM.zip › 10735_2024_10185_MOESM1_ESM/Supplementary Material/Fig4B-HO8910-OE-GSK3.jpg]

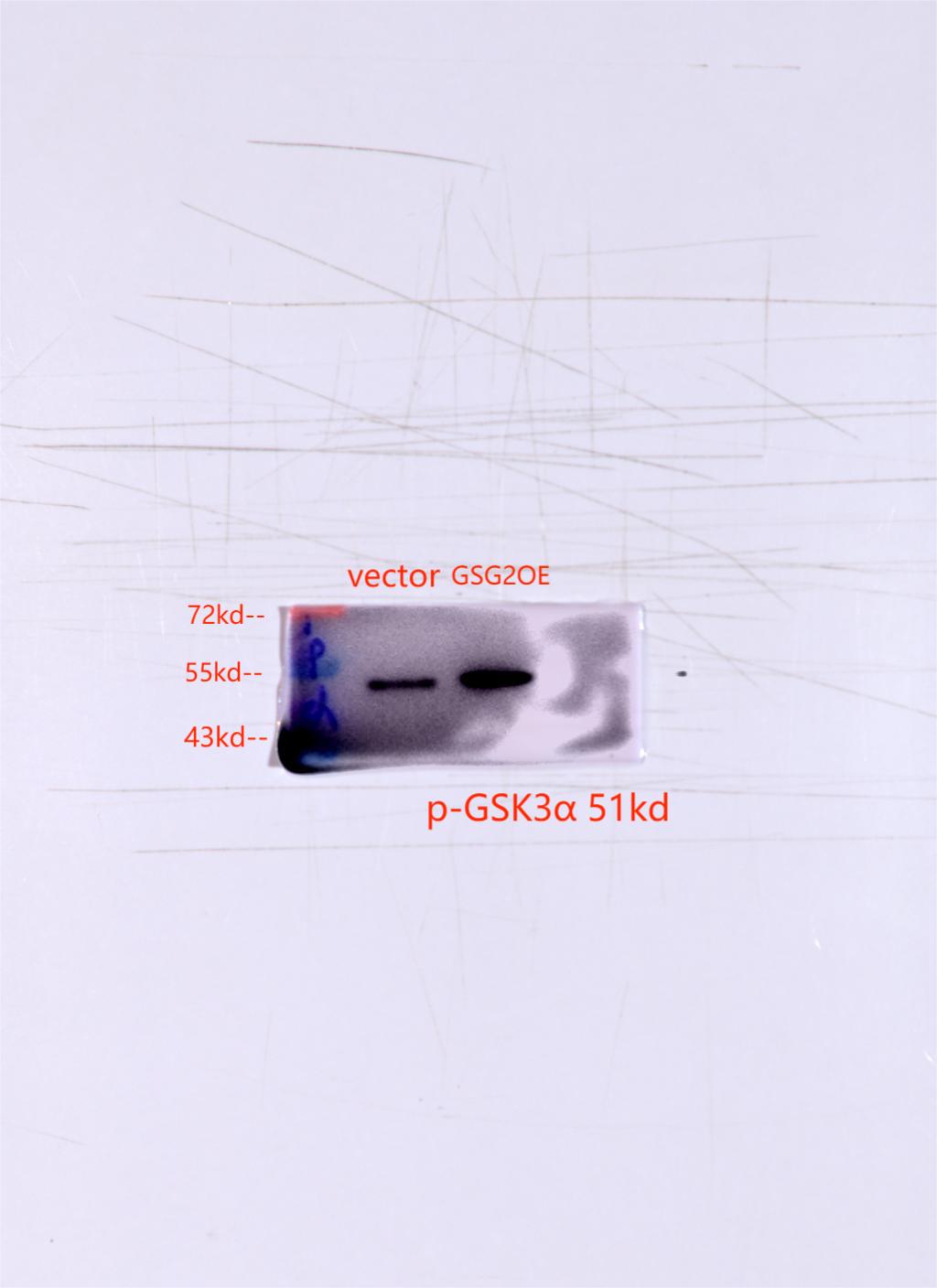

Supplement: Supplementary file 1 — Supplementary file1 (ZIP 11,586 kb) [file 10735_2024_10185_MOESM1_ESM.zip › 10735_2024_10185_MOESM1_ESM/Supplementary Material/Fig4B-HO8910-OE-pGSK3A.jpg]

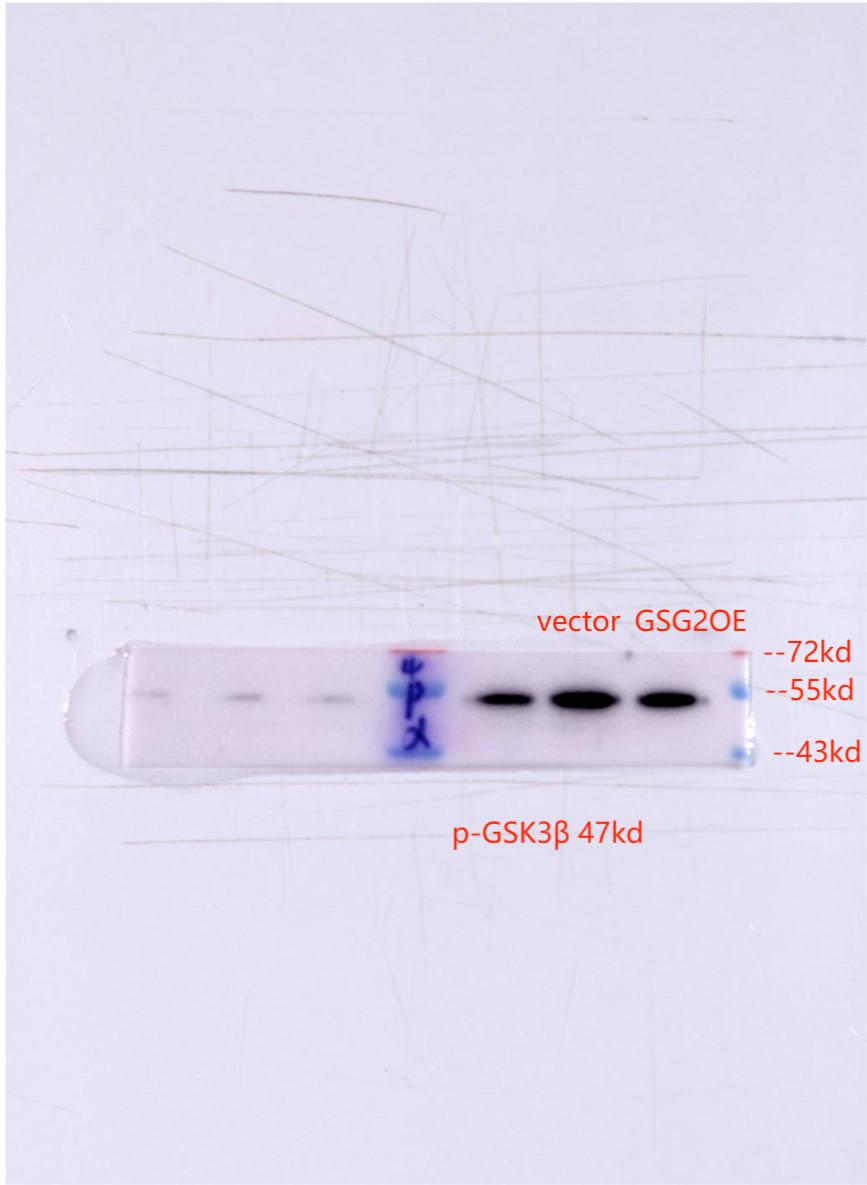

Supplement: Supplementary file 1 — Supplementary file1 (ZIP 11,586 kb) [file 10735_2024_10185_MOESM1_ESM.zip › 10735_2024_10185_MOESM1_ESM/Supplementary Material/Fig4B-HO8910-OE-pGSK3B.jpg]

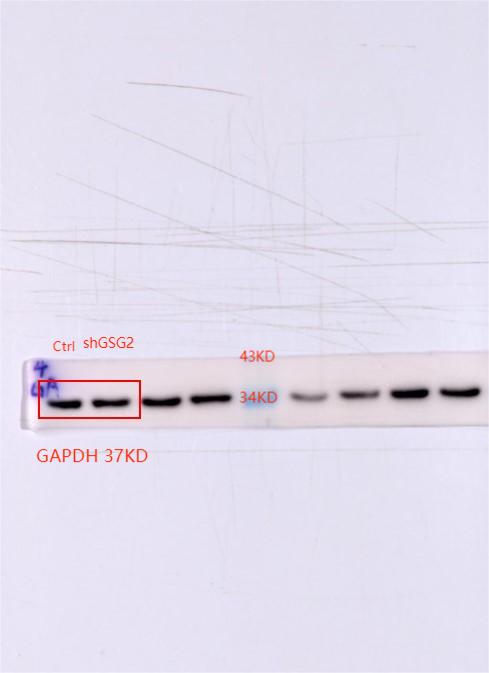

Supplement: Supplementary file 1 — Supplementary file1 (ZIP 11,586 kb) [file 10735_2024_10185_MOESM1_ESM.zip › 10735_2024_10185_MOESM1_ESM/Supplementary Material/Fig4B-HO8910-SH-GAPDH .jpg]

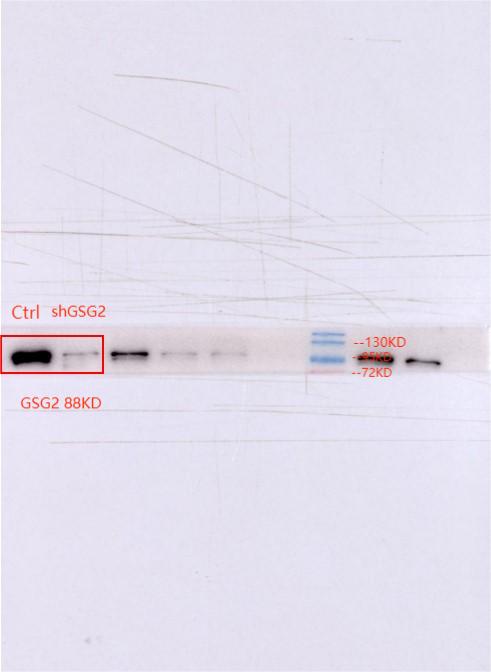

Supplement: Supplementary file 1 — Supplementary file1 (ZIP 11,586 kb) [file 10735_2024_10185_MOESM1_ESM.zip › 10735_2024_10185_MOESM1_ESM/Supplementary Material/Fig4B-HO8910-sh-GSG2.jpg]

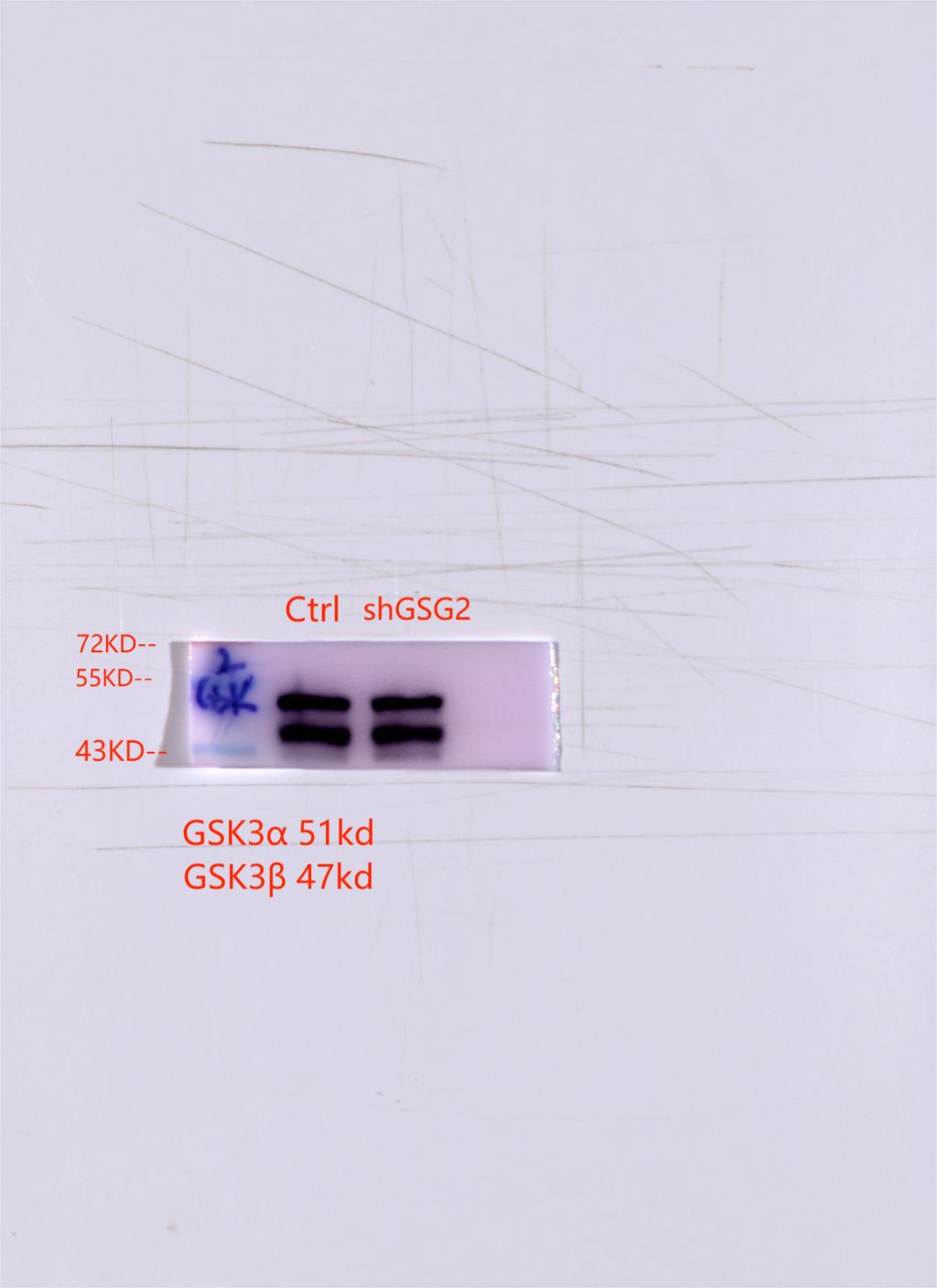

Supplement: Supplementary file 1 — Supplementary file1 (ZIP 11,586 kb) [file 10735_2024_10185_MOESM1_ESM.zip › 10735_2024_10185_MOESM1_ESM/Supplementary Material/Fig4B-HO8910-SH-GSK3 .jpg]

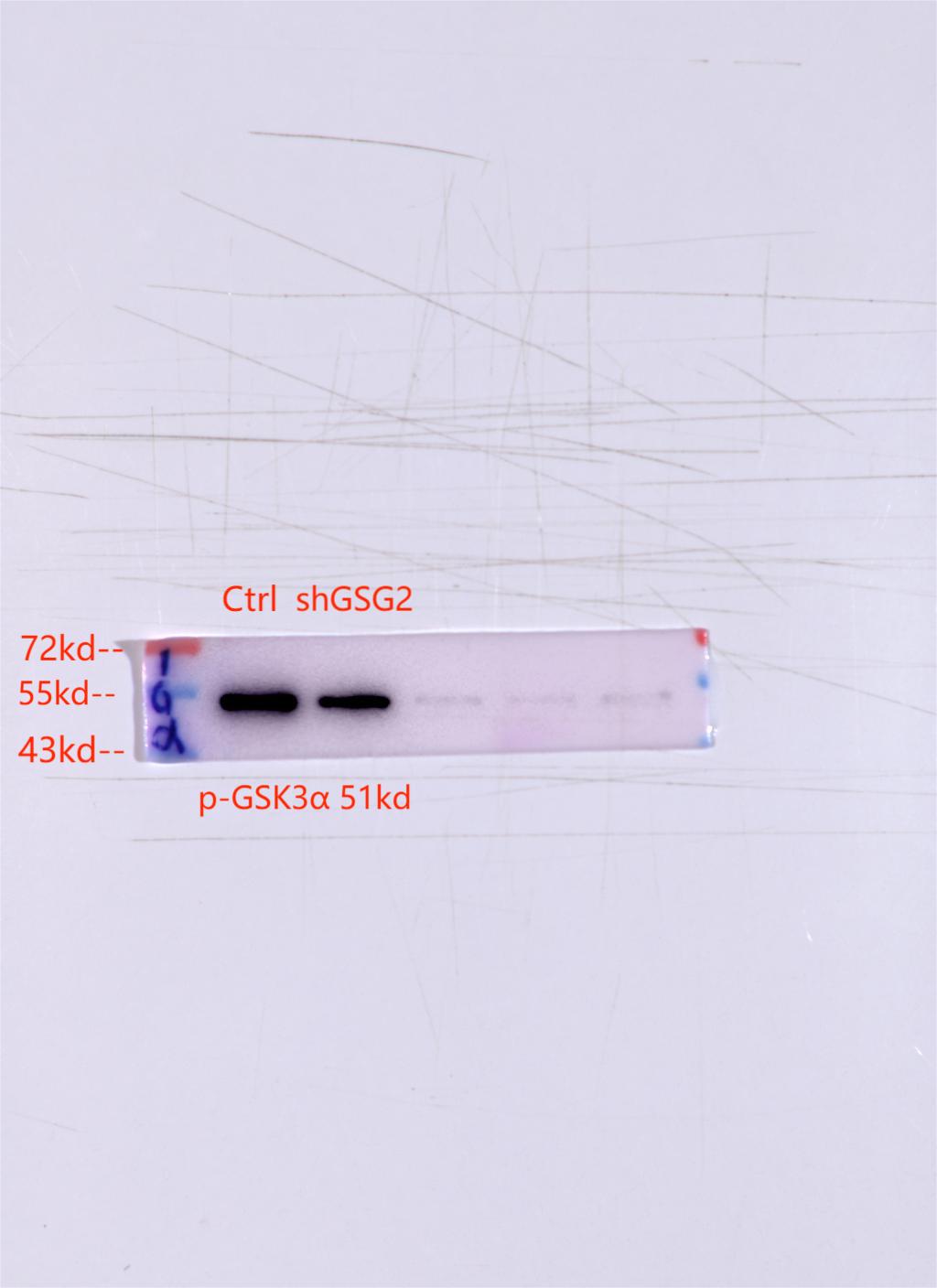

Supplement: Supplementary file 1 — Supplementary file1 (ZIP 11,586 kb) [file 10735_2024_10185_MOESM1_ESM.zip › 10735_2024_10185_MOESM1_ESM/Supplementary Material/Fig4B-HO8910-SH-pGSK3A.jpg]

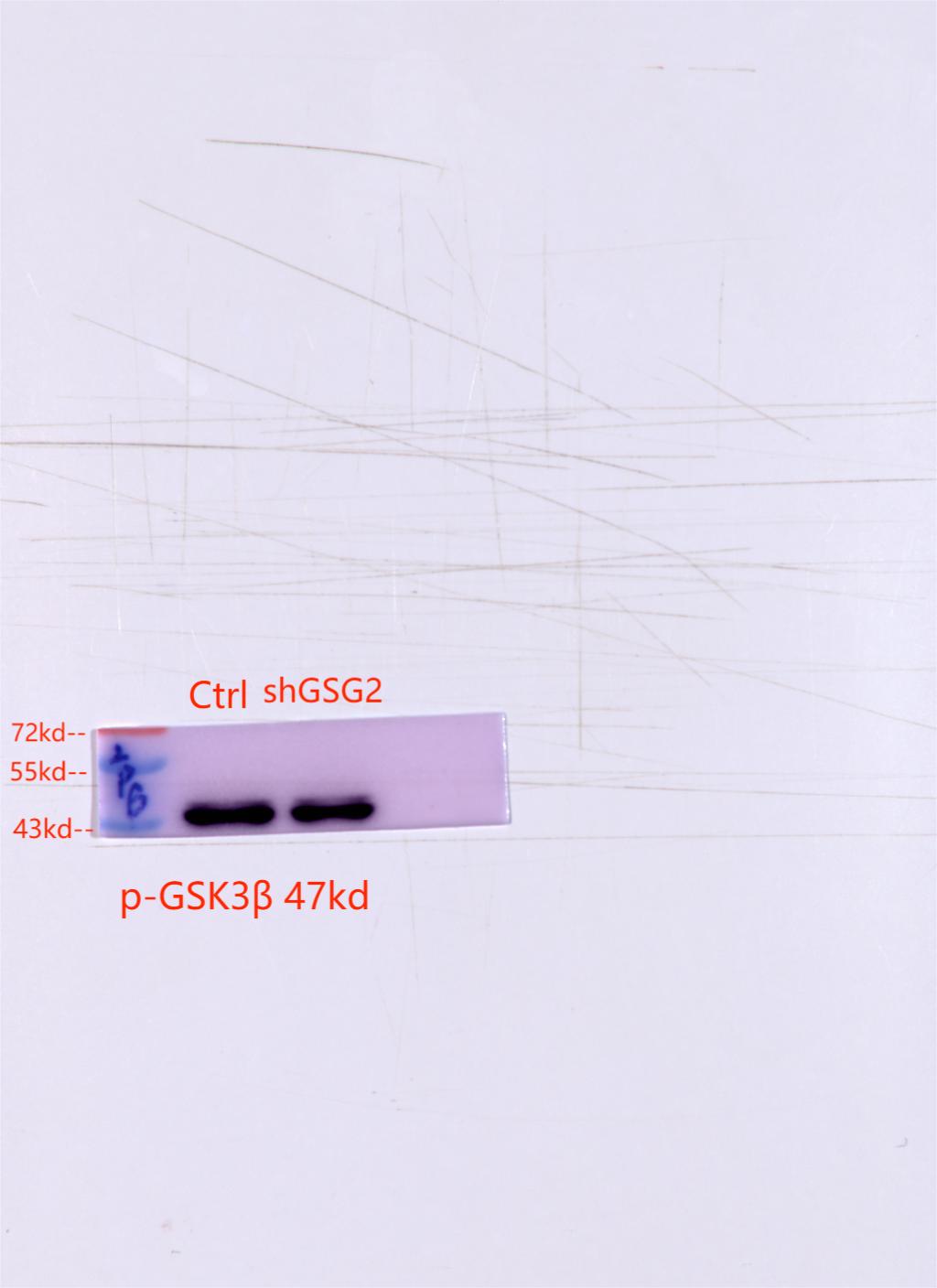

Supplement: Supplementary file 1 — Supplementary file1 (ZIP 11,586 kb) [file 10735_2024_10185_MOESM1_ESM.zip › 10735_2024_10185_MOESM1_ESM/Supplementary Material/Fig4B-HO8910-SH-pGSK3b .jpg]

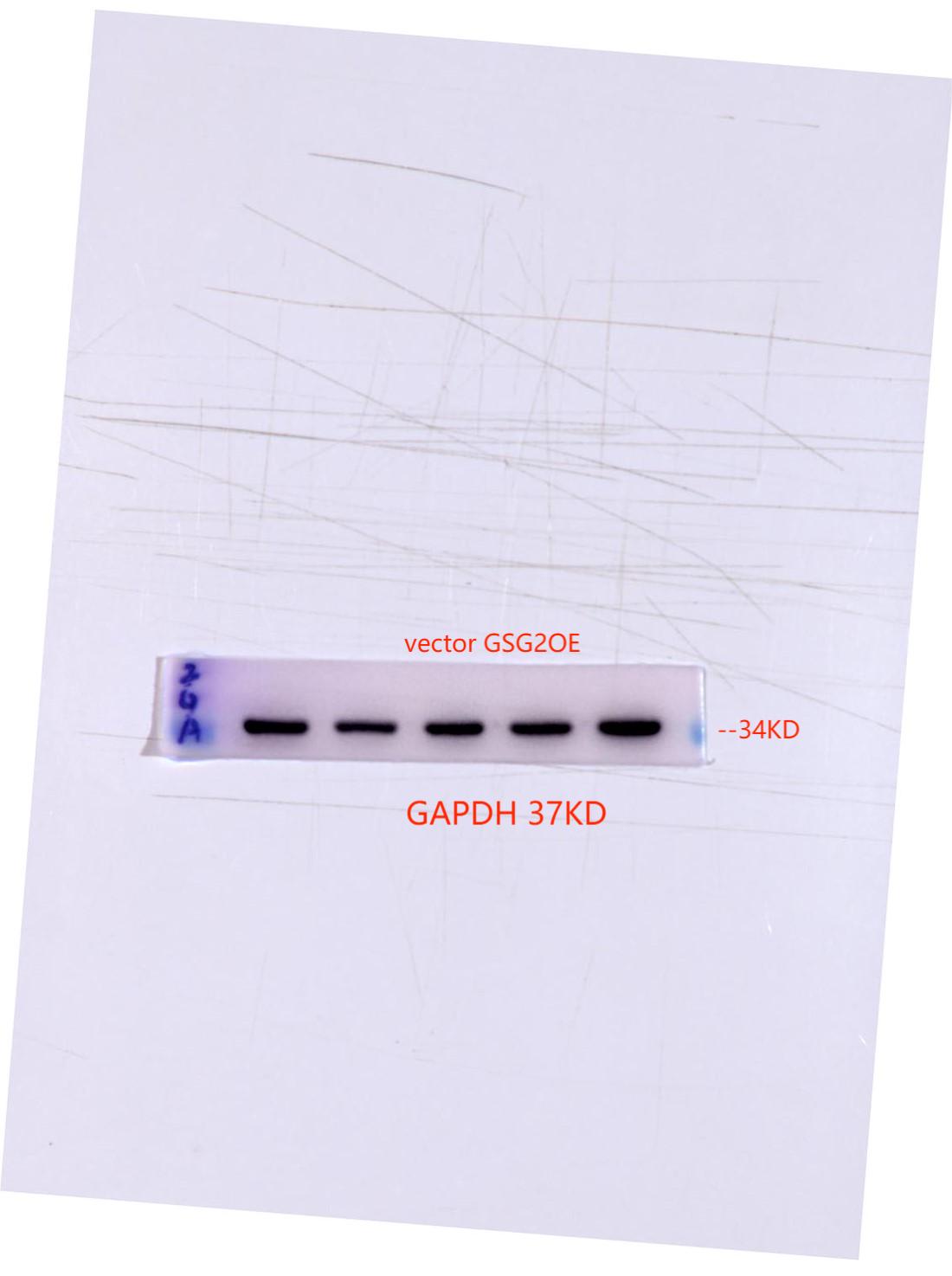

Supplement: Supplementary file 1 — Supplementary file1 (ZIP 11,586 kb) [file 10735_2024_10185_MOESM1_ESM.zip › 10735_2024_10185_MOESM1_ESM/Supplementary Material/Fig4B-SKOV3-OE-GAPDH.jpg]

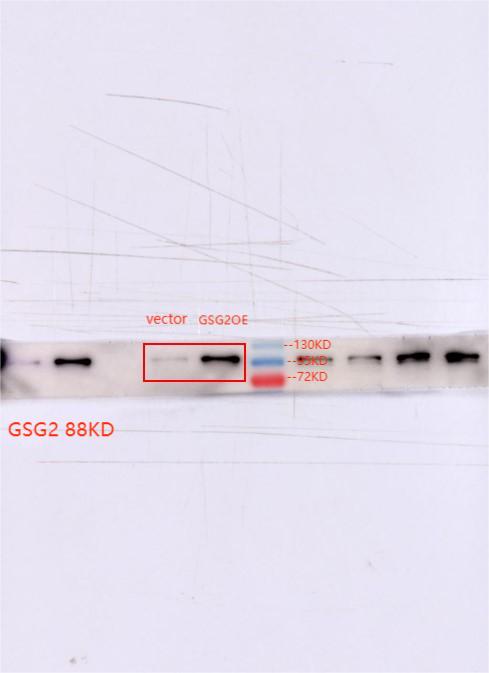

Supplement: Supplementary file 1 — Supplementary file1 (ZIP 11,586 kb) [file 10735_2024_10185_MOESM1_ESM.zip › 10735_2024_10185_MOESM1_ESM/Supplementary Material/Fig4B-SKOV3-OE-GSG2.jpg]

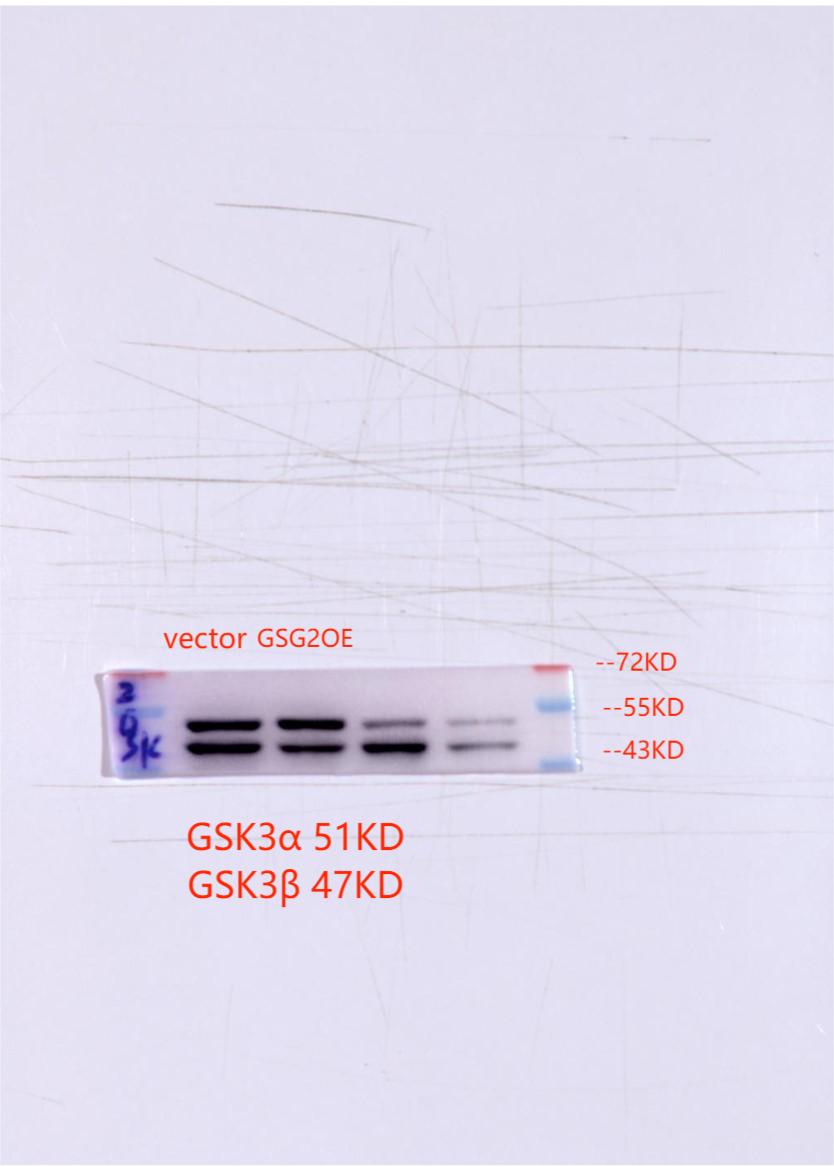

Supplement: Supplementary file 1 — Supplementary file1 (ZIP 11,586 kb) [file 10735_2024_10185_MOESM1_ESM.zip › 10735_2024_10185_MOESM1_ESM/Supplementary Material/Fig4B-SKOV3-OE-GSK3.jpg]

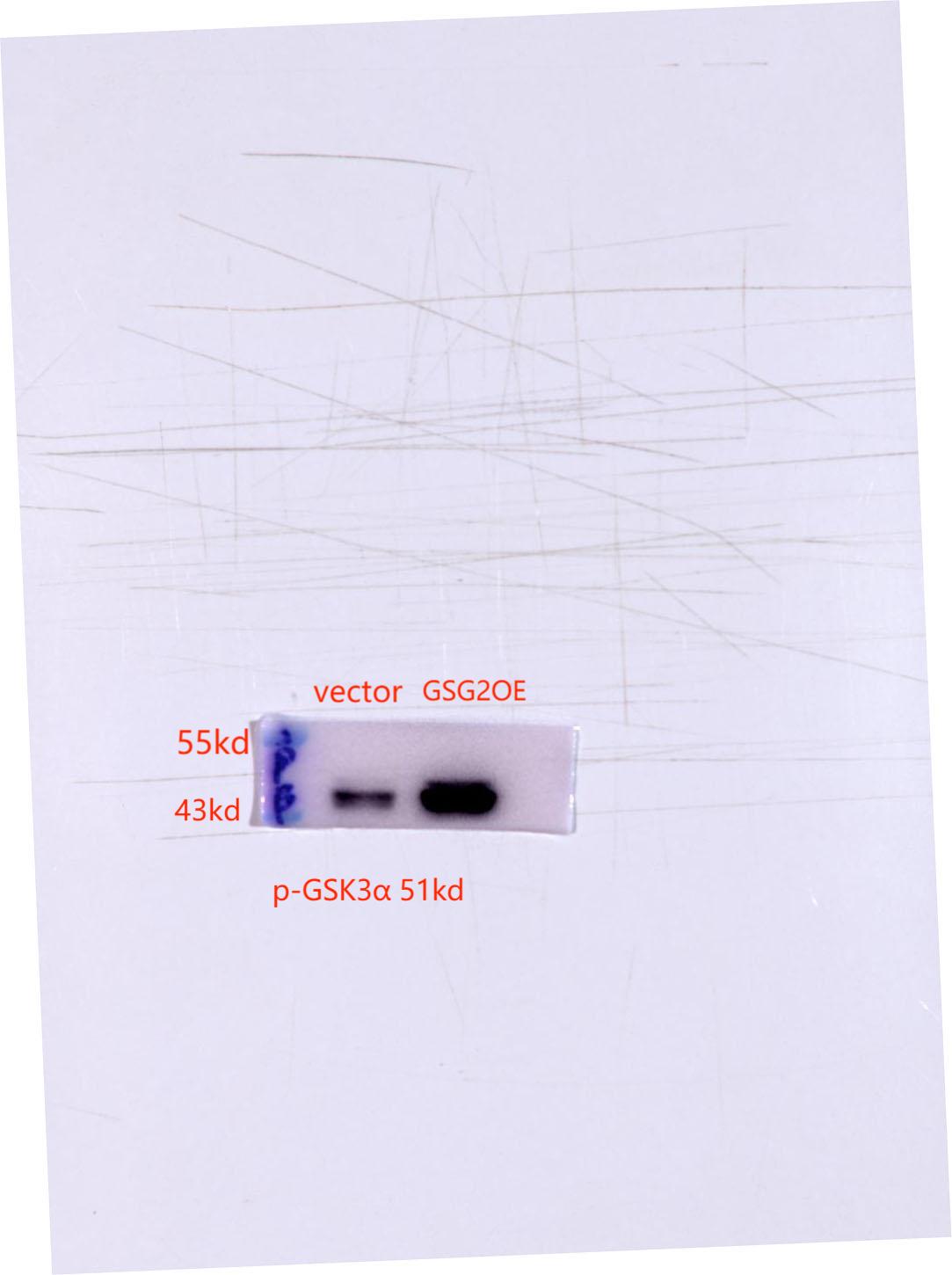

Supplement: Supplementary file 1 — Supplementary file1 (ZIP 11,586 kb) [file 10735_2024_10185_MOESM1_ESM.zip › 10735_2024_10185_MOESM1_ESM/Supplementary Material/Fig4B-SKOV3-OE-pGSK3A.jpg]

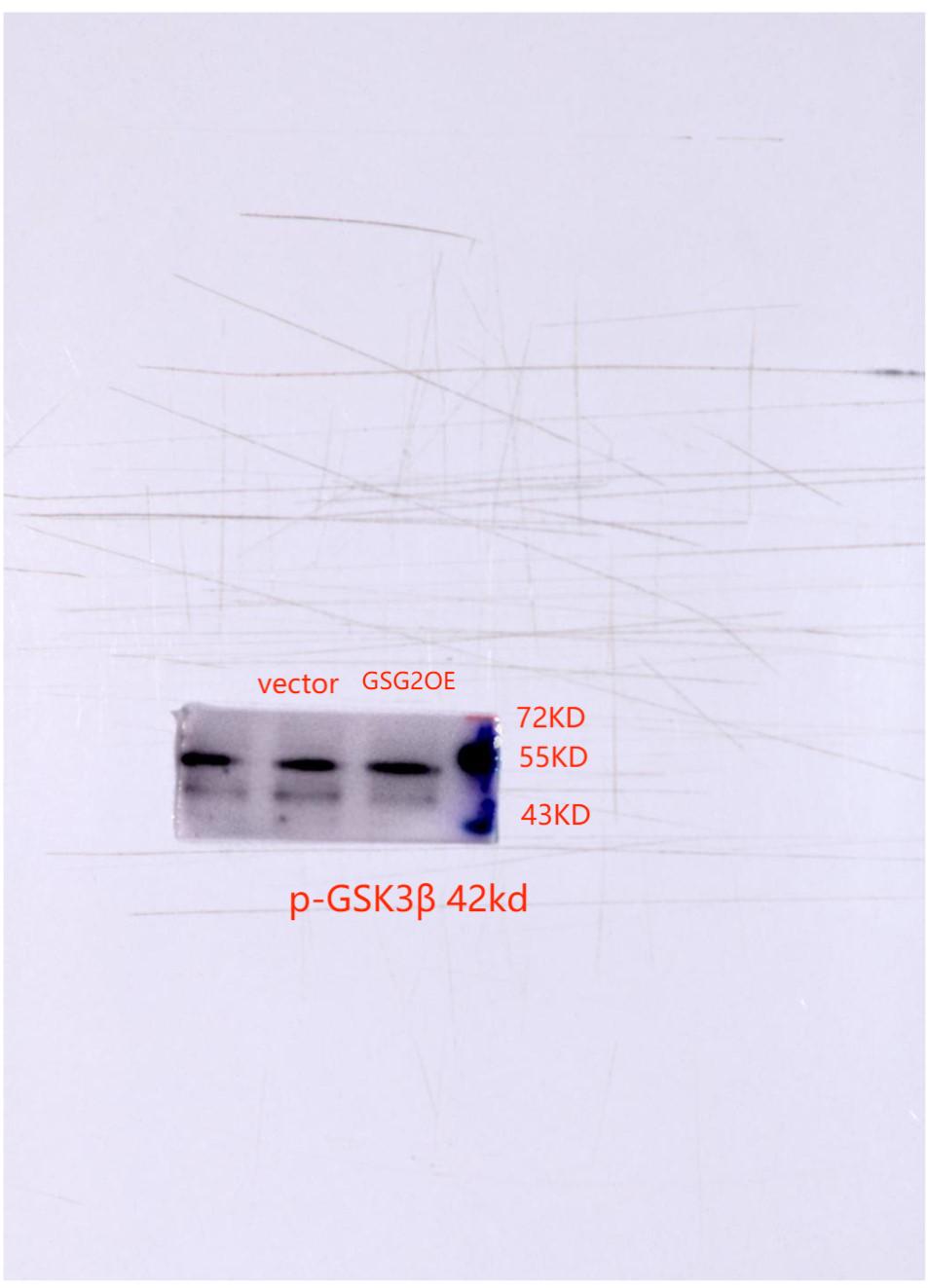

Supplement: Supplementary file 1 — Supplementary file1 (ZIP 11,586 kb) [file 10735_2024_10185_MOESM1_ESM.zip › 10735_2024_10185_MOESM1_ESM/Supplementary Material/Fig4B-SKOV3-OE-pGSK3B.jpg]

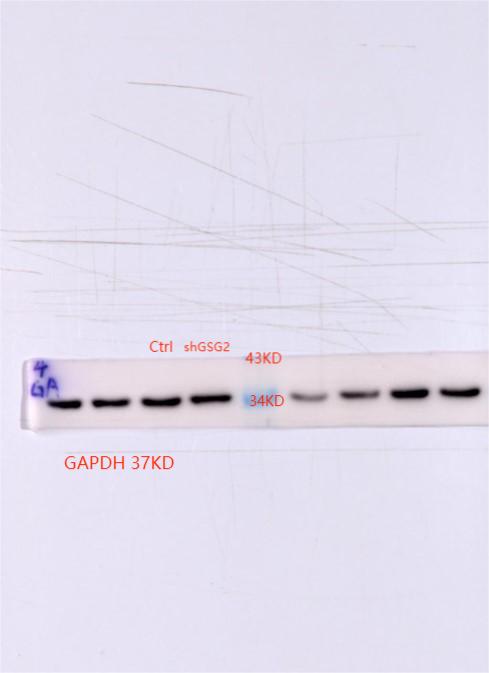

Supplement: Supplementary file 1 — Supplementary file1 (ZIP 11,586 kb) [file 10735_2024_10185_MOESM1_ESM.zip › 10735_2024_10185_MOESM1_ESM/Supplementary Material/Fig4B-SKOV3-SH-GAPDH .jpg]

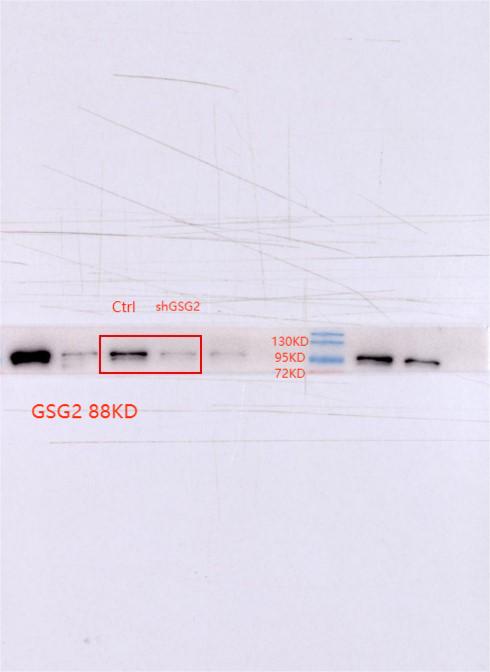

Supplement: Supplementary file 1 — Supplementary file1 (ZIP 11,586 kb) [file 10735_2024_10185_MOESM1_ESM.zip › 10735_2024_10185_MOESM1_ESM/Supplementary Material/Fig4B-SKOV3-sh-GSG2.jpg]

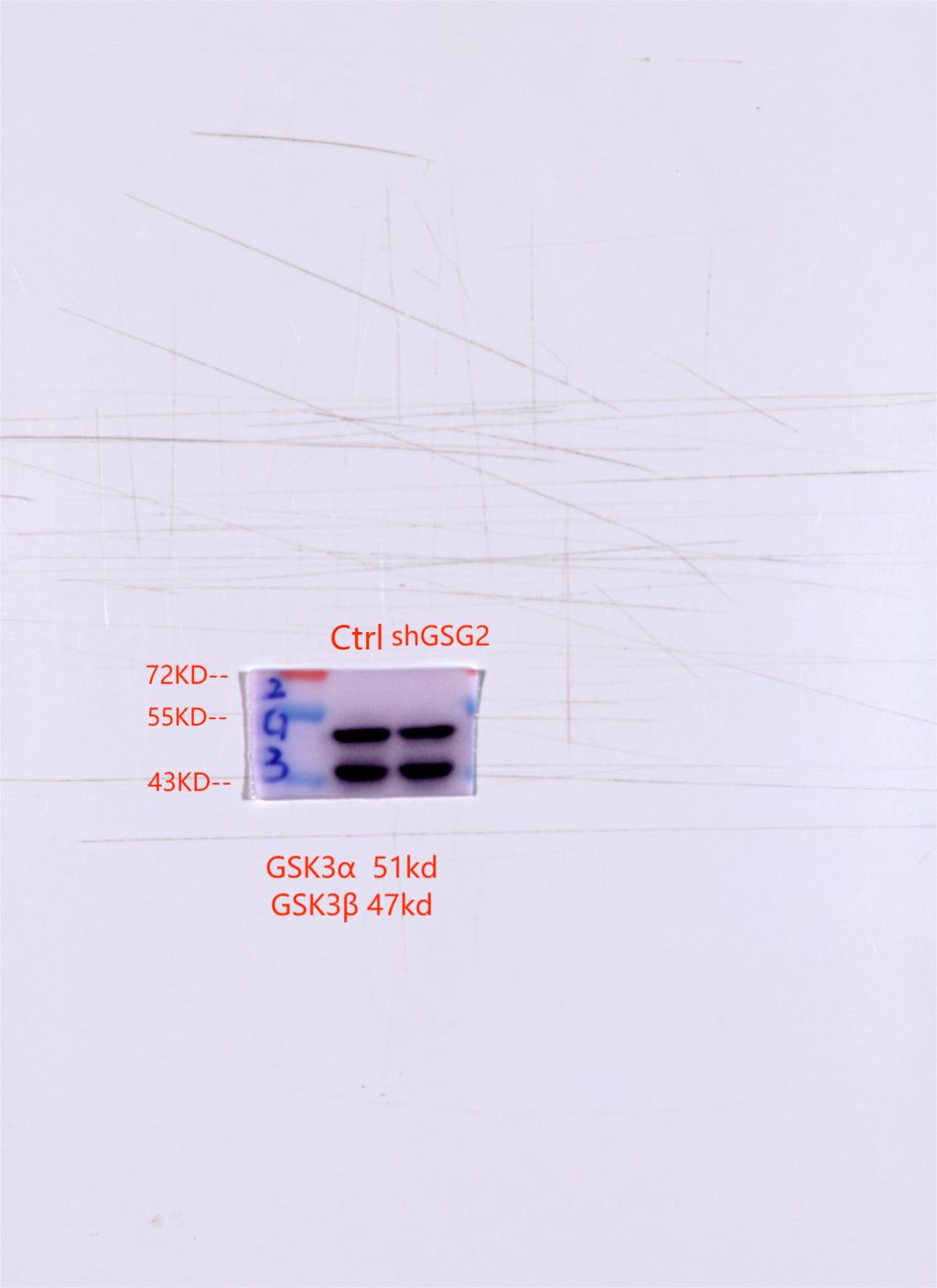

Supplement: Supplementary file 1 — Supplementary file1 (ZIP 11,586 kb) [file 10735_2024_10185_MOESM1_ESM.zip › 10735_2024_10185_MOESM1_ESM/Supplementary Material/Fig4B-SKOV3-SH-GSK3 .jpg]

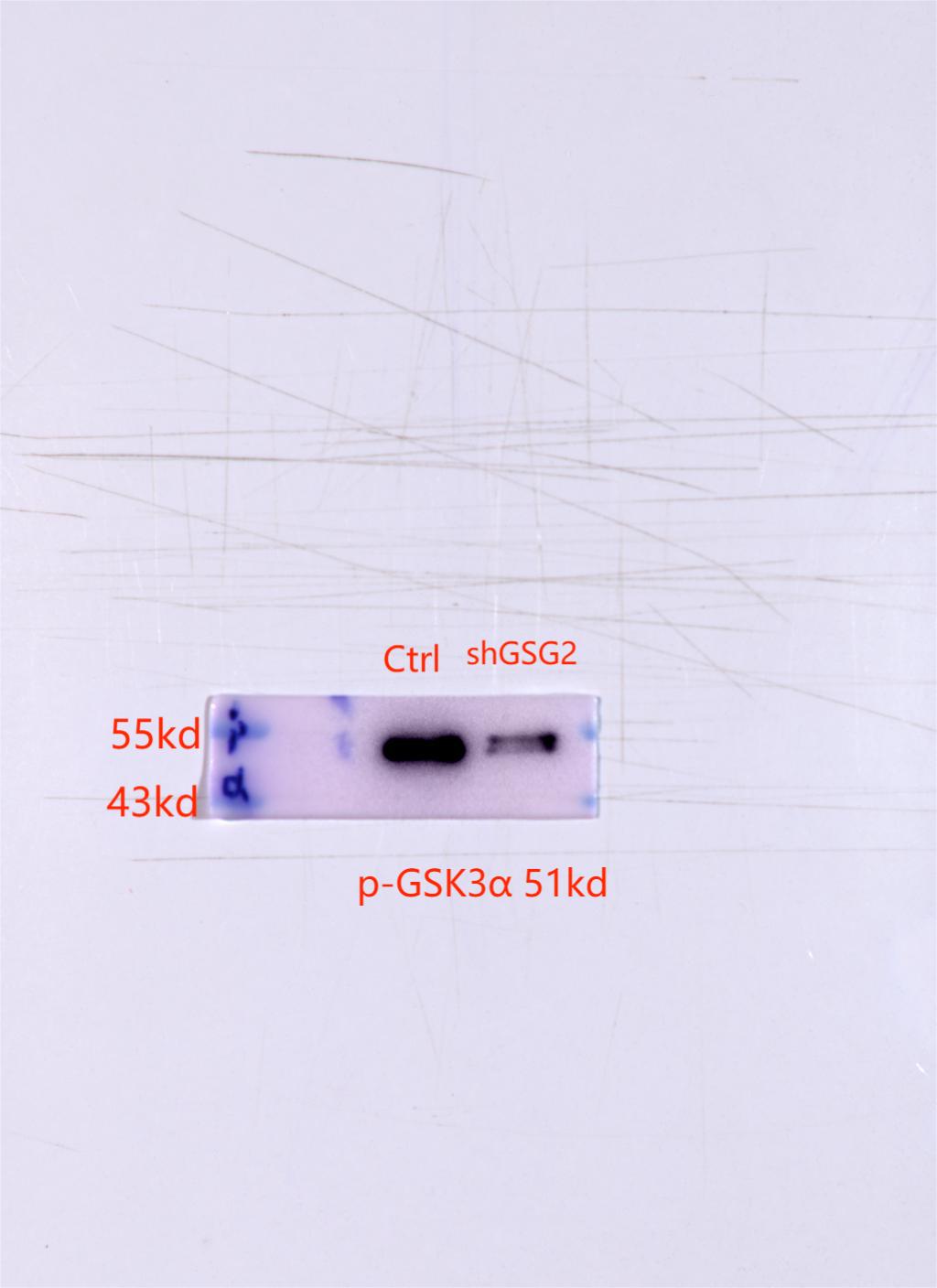

Supplement: Supplementary file 1 — Supplementary file1 (ZIP 11,586 kb) [file 10735_2024_10185_MOESM1_ESM.zip › 10735_2024_10185_MOESM1_ESM/Supplementary Material/Fig4B-SKOV3-SH-pGSK3A.jpg]

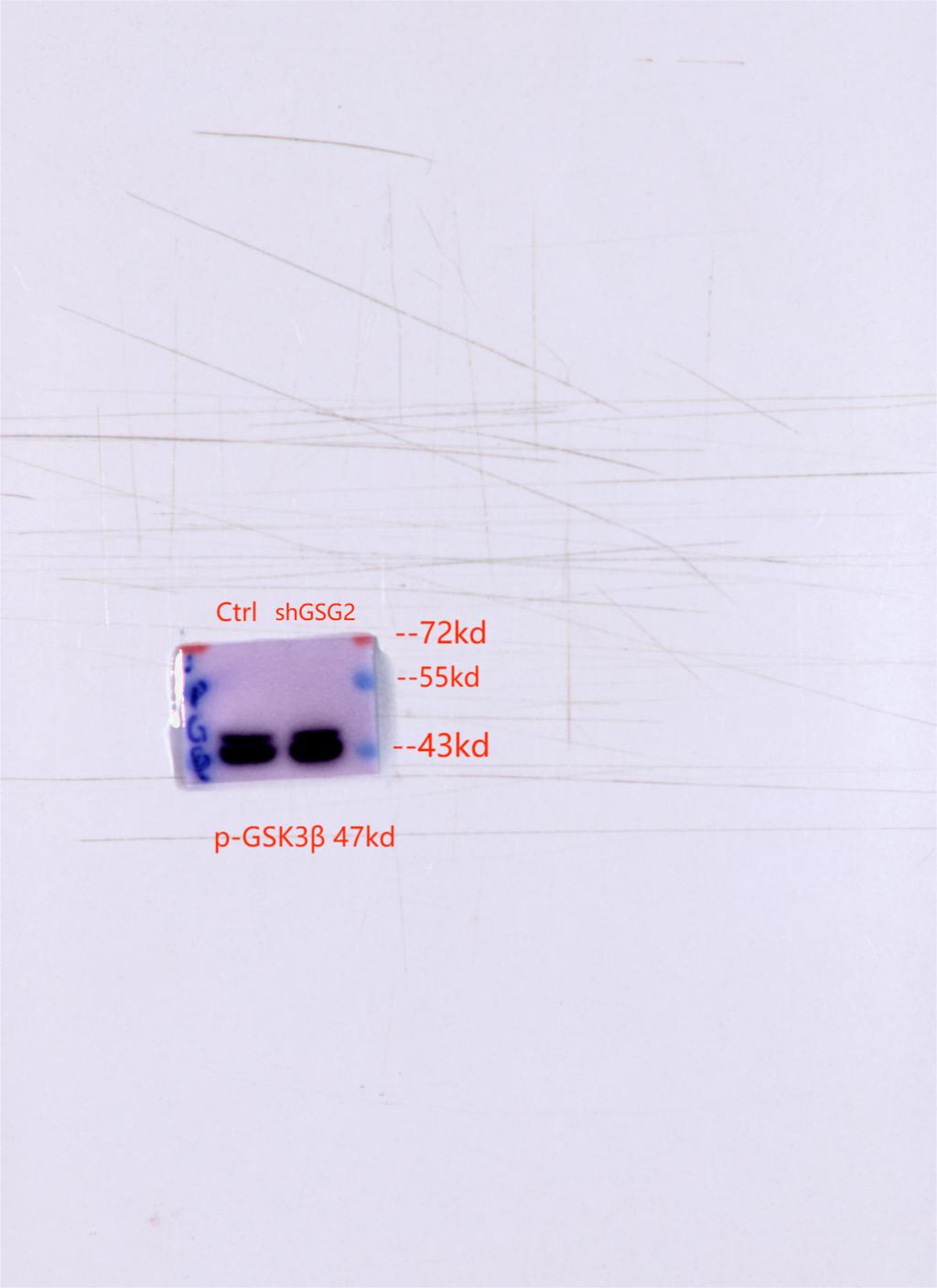

Supplement: Supplementary file 1 — Supplementary file1 (ZIP 11,586 kb) [file 10735_2024_10185_MOESM1_ESM.zip › 10735_2024_10185_MOESM1_ESM/Supplementary Material/Fig4B-SKOV3-SH-pGSK3B.jpg]

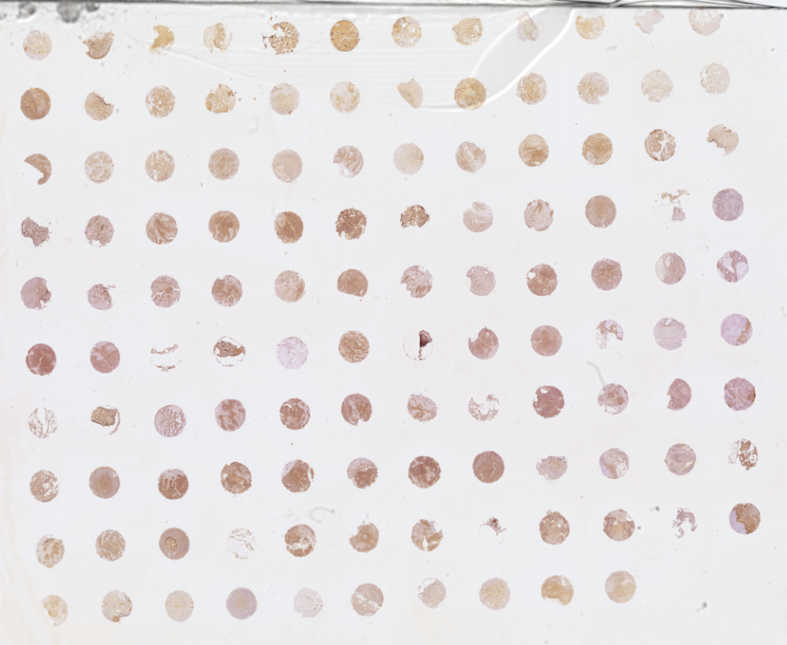

Supplement: Supplementary file 1 — Supplementary file1 (ZIP 11,586 kb) [file 10735_2024_10185_MOESM1_ESM.zip › 10735_2024_10185_MOESM1_ESM/Supplementary Material/Fig4C-IHC-GSG2.png]

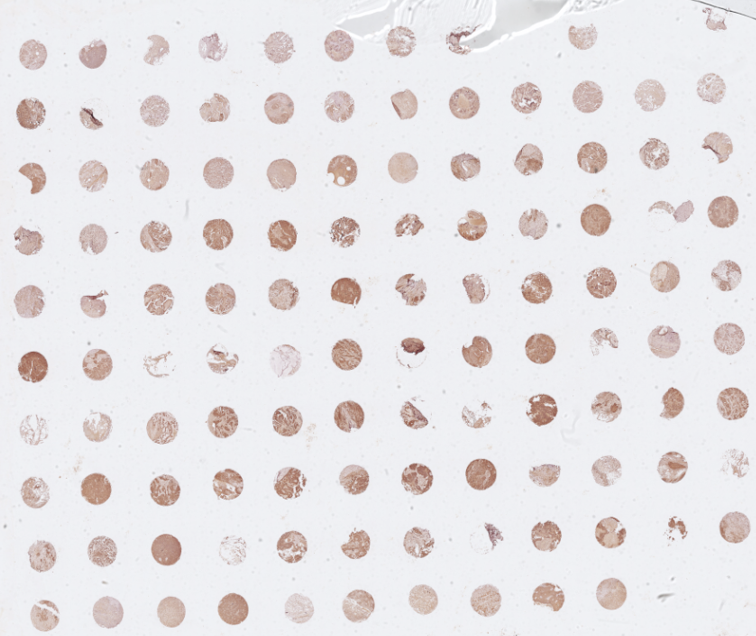

Supplement: Supplementary file 1 — Supplementary file1 (ZIP 11,586 kb) [file 10735_2024_10185_MOESM1_ESM.zip › 10735_2024_10185_MOESM1_ESM/Supplementary Material/Fig4C-IHC-p-GSK3A.png]

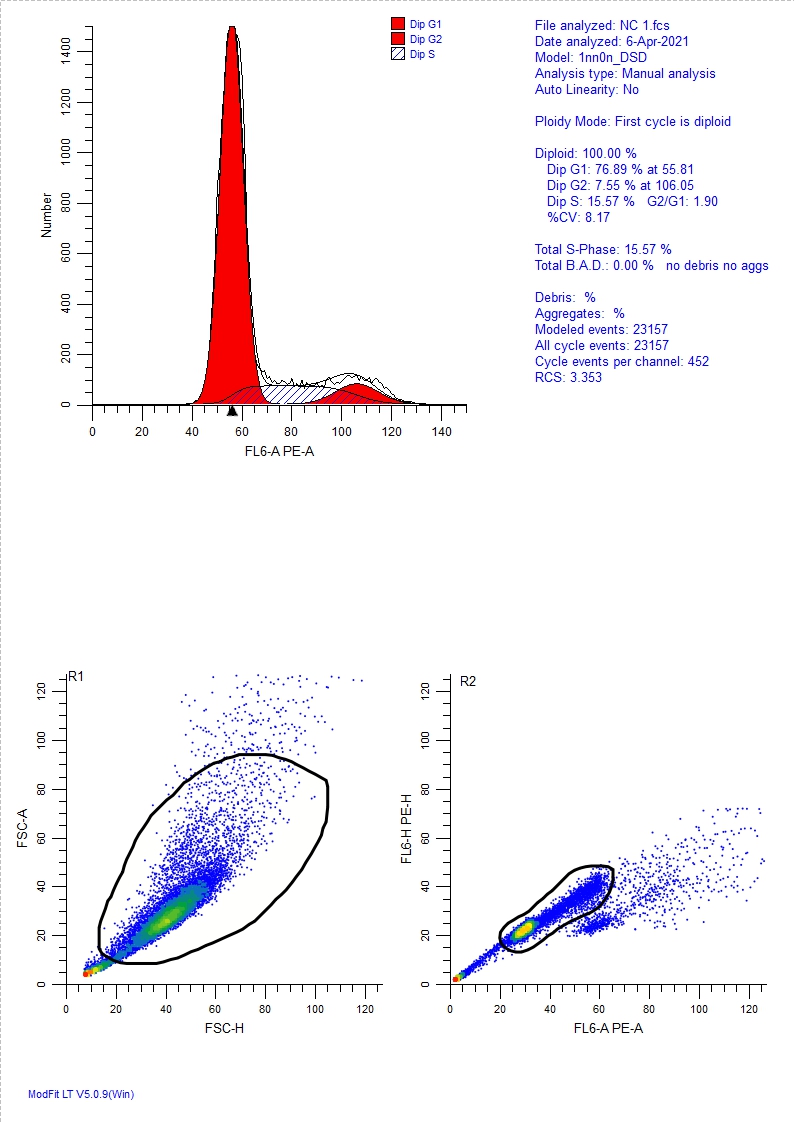

Supplement: Supplementary file 1 — Supplementary file1 (ZIP 11,586 kb) [file 10735_2024_10185_MOESM1_ESM.zip › 10735_2024_10185_MOESM1_ESM/Supplementary Material/Fig5B-HO8910-shCtrl.jpg]

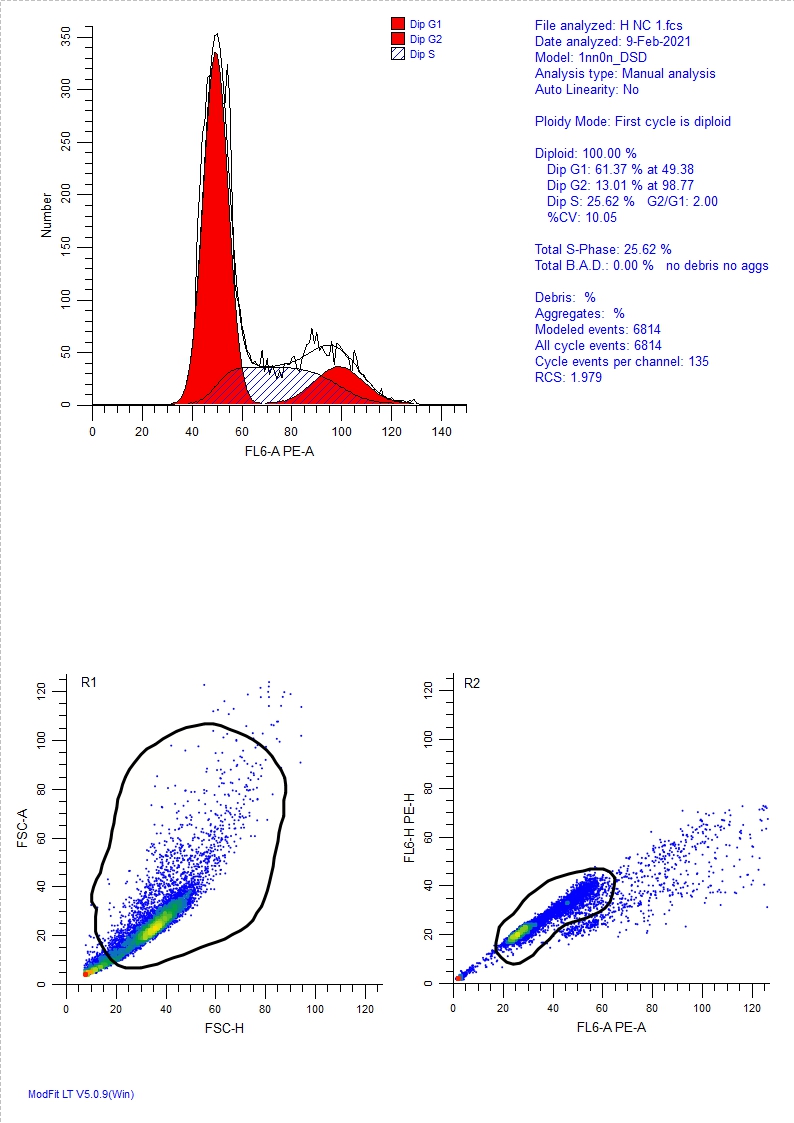

Supplement: Supplementary file 1 — Supplementary file1 (ZIP 11,586 kb) [file 10735_2024_10185_MOESM1_ESM.zip › 10735_2024_10185_MOESM1_ESM/Supplementary Material/Fig5B-HO8910-shGSG2-BRD0705(-) .jpg]

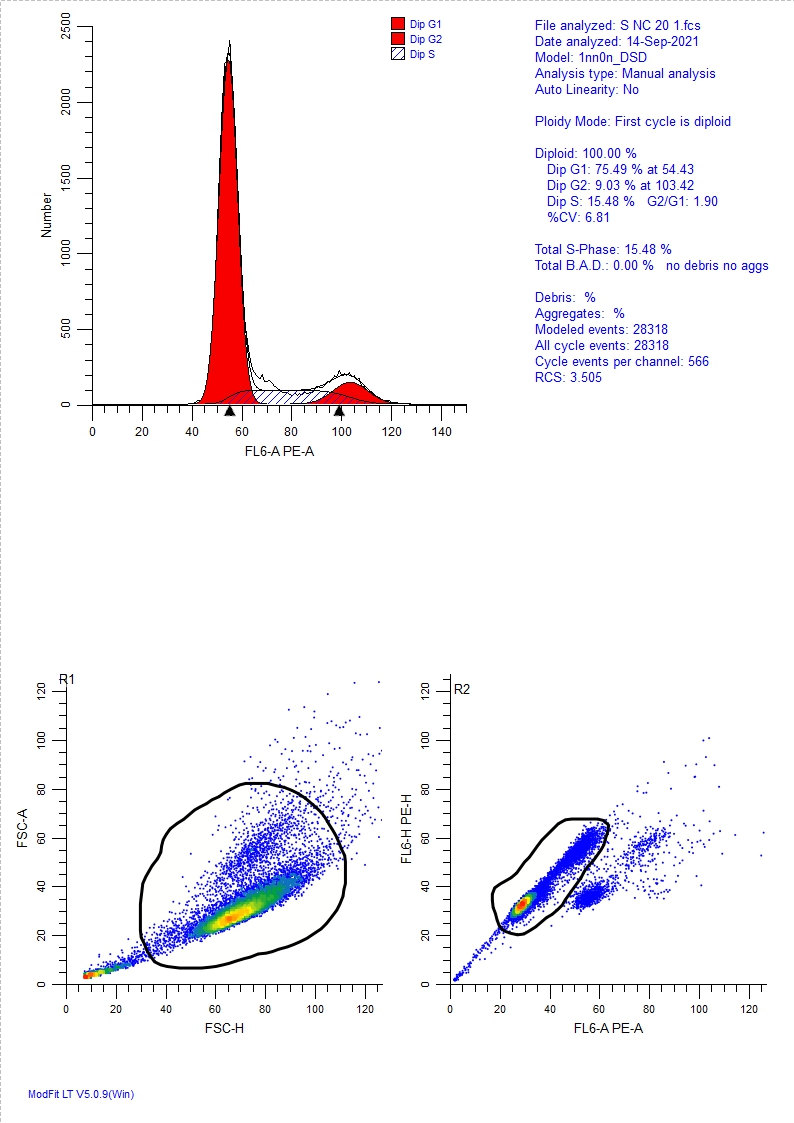

Supplement: Supplementary file 1 — Supplementary file1 (ZIP 11,586 kb) [file 10735_2024_10185_MOESM1_ESM.zip › 10735_2024_10185_MOESM1_ESM/Supplementary Material/Fig5B-HO8910-shGSG2-BRD0705(+).jpg]

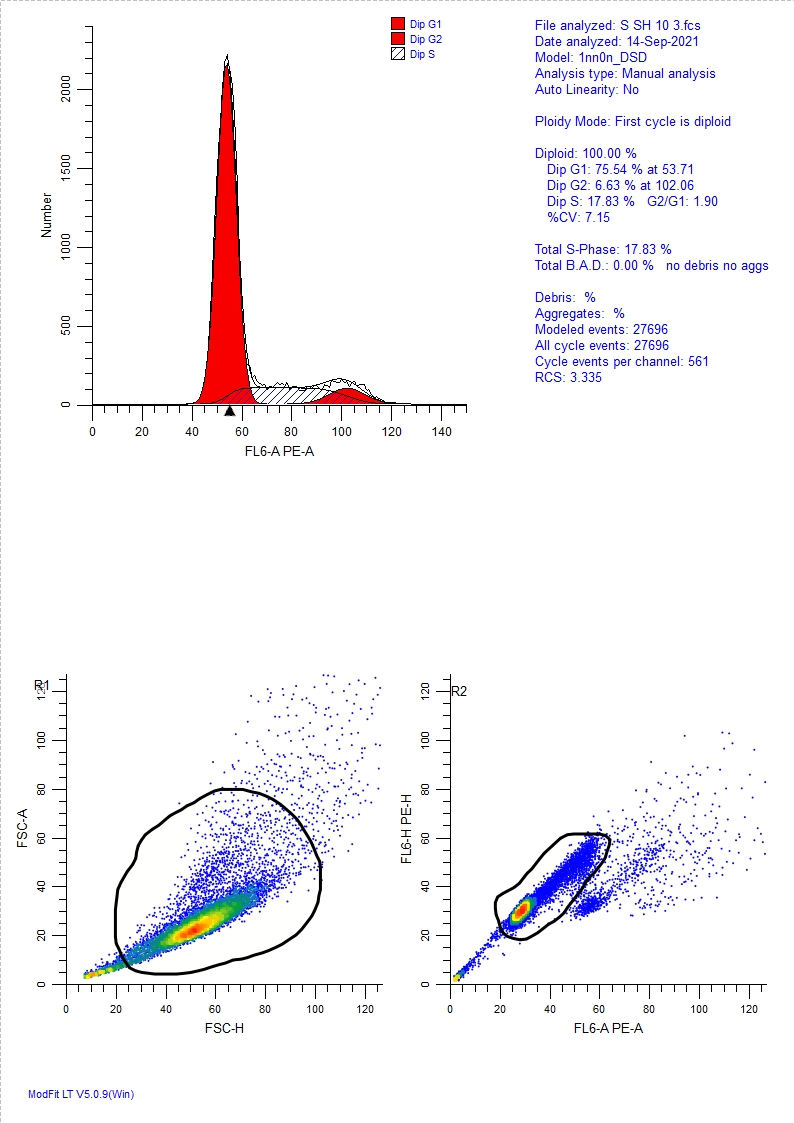

Supplement: Supplementary file 1 — Supplementary file1 (ZIP 11,586 kb) [file 10735_2024_10185_MOESM1_ESM.zip › 10735_2024_10185_MOESM1_ESM/Supplementary Material/Fig5B-HO8910-shGSG2-BRD0705(++).jpg]

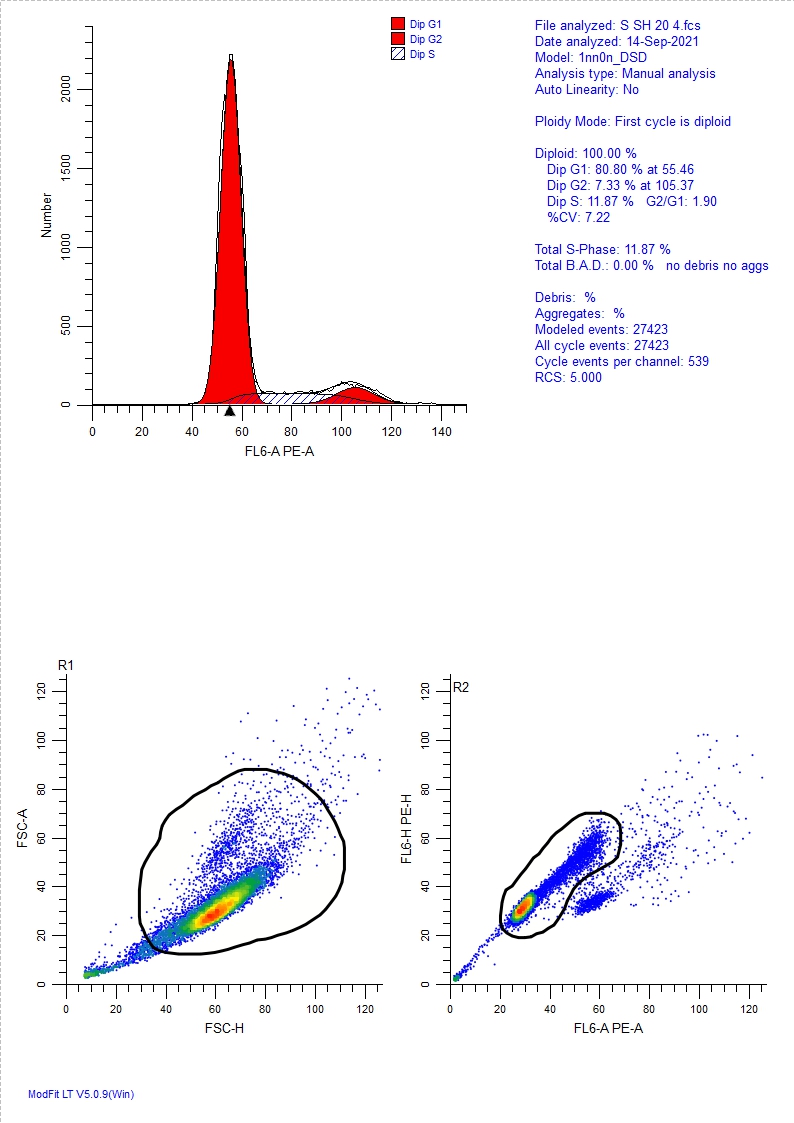

Supplement: Supplementary file 1 — Supplementary file1 (ZIP 11,586 kb) [file 10735_2024_10185_MOESM1_ESM.zip › 10735_2024_10185_MOESM1_ESM/Supplementary Material/Fig5B-SKOV3-shGSG2-BRD0705(++).jpg]

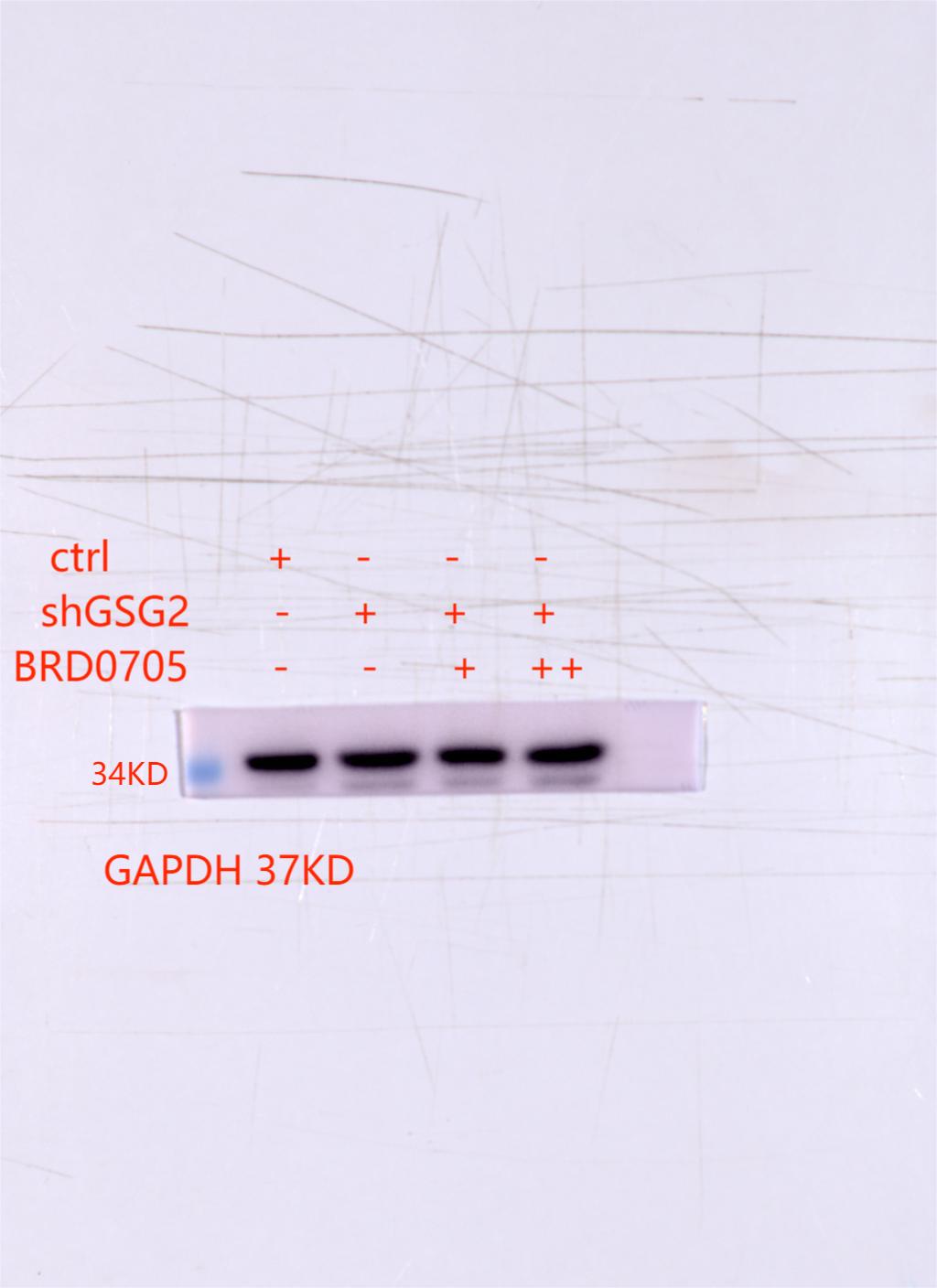

Supplement: Supplementary file 1 — Supplementary file1 (ZIP 11,586 kb) [file 10735_2024_10185_MOESM1_ESM.zip › 10735_2024_10185_MOESM1_ESM/Supplementary Material/Fig6A-HO8910-GAPDH.jpg]

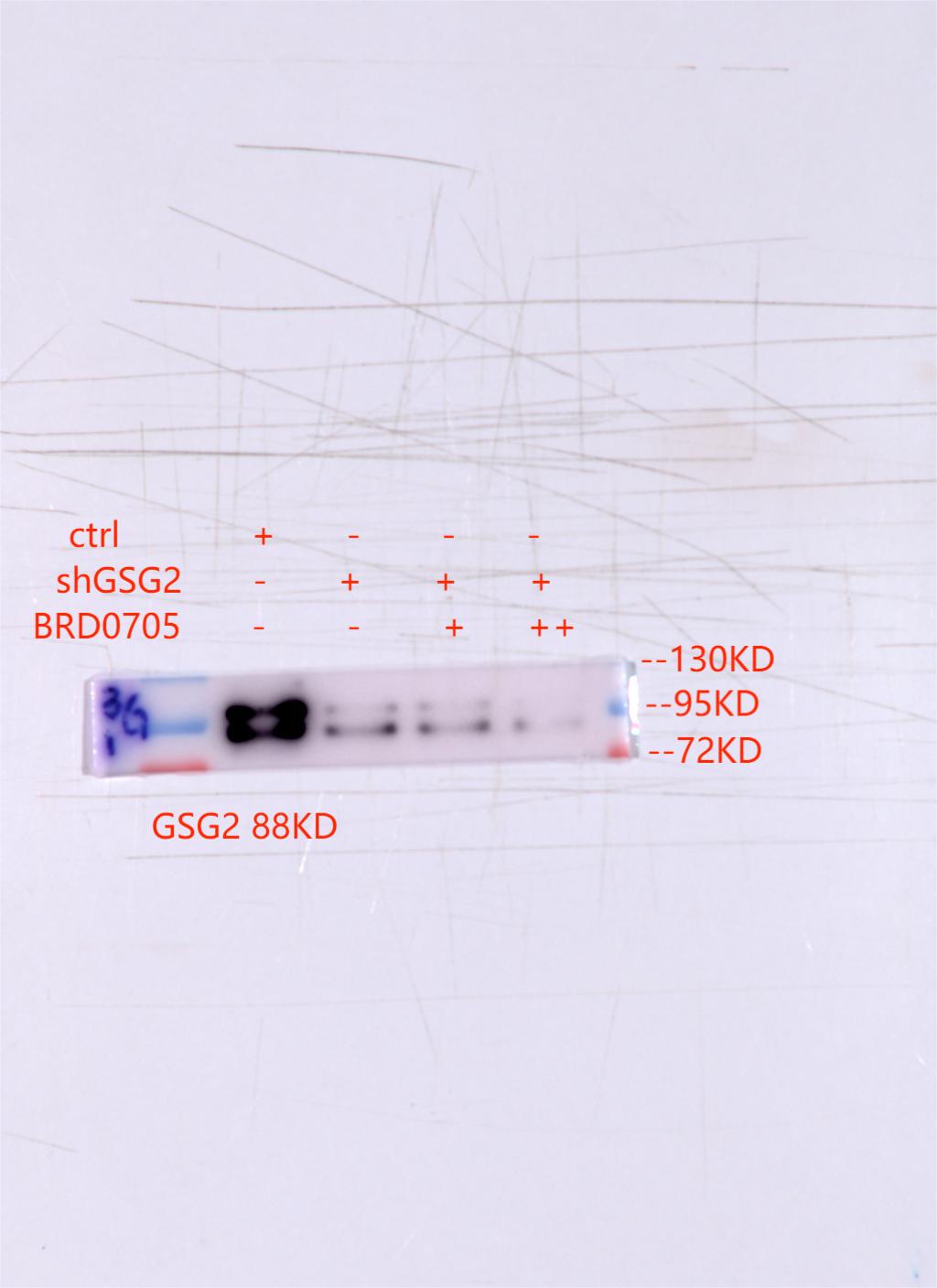

Supplement: Supplementary file 1 — Supplementary file1 (ZIP 11,586 kb) [file 10735_2024_10185_MOESM1_ESM.zip › 10735_2024_10185_MOESM1_ESM/Supplementary Material/Fig6A-HO8910-GSG2.jpg]

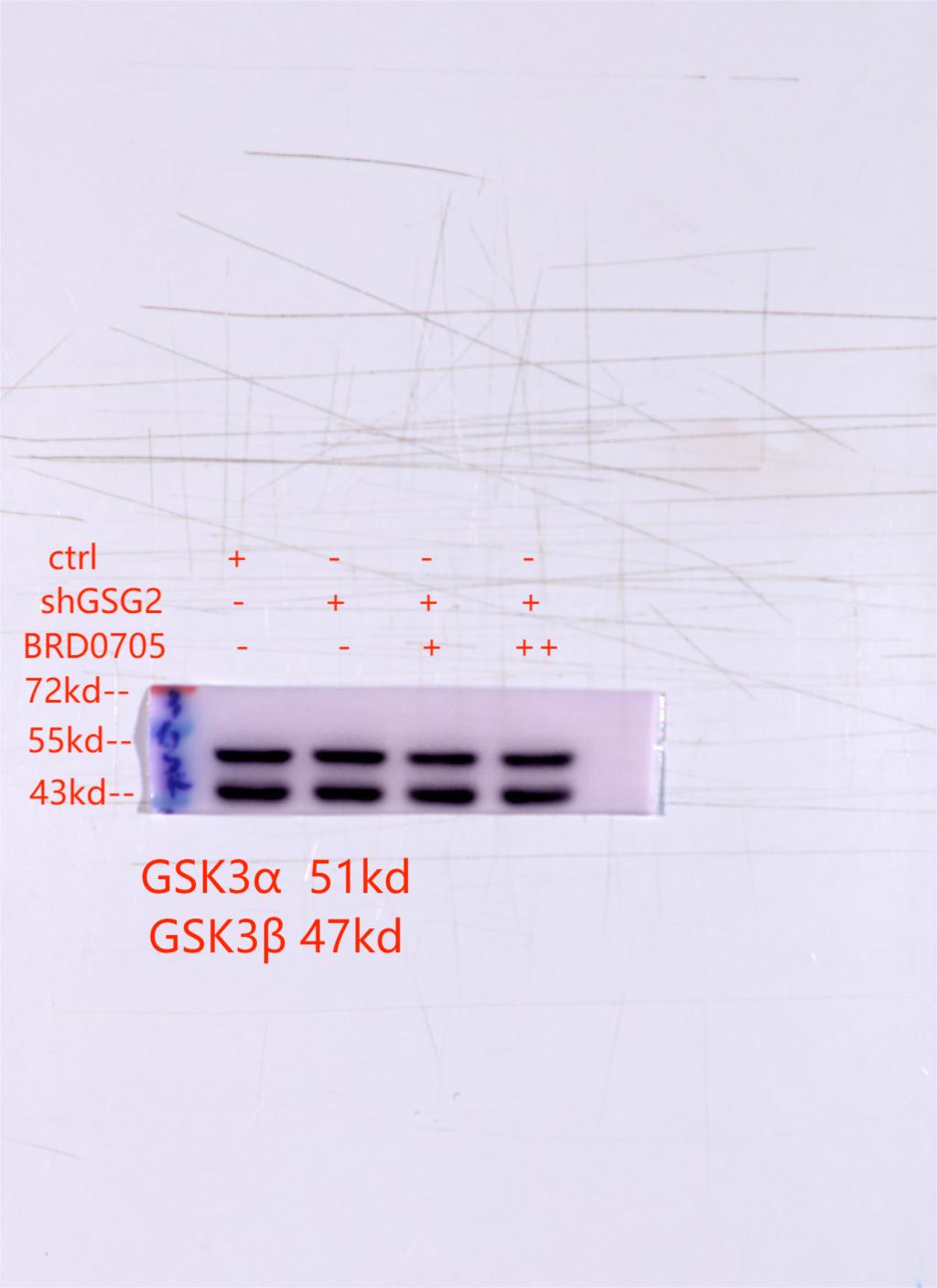

Supplement: Supplementary file 1 — Supplementary file1 (ZIP 11,586 kb) [file 10735_2024_10185_MOESM1_ESM.zip › 10735_2024_10185_MOESM1_ESM/Supplementary Material/Fig6A-HO8910-GSK3.jpg]

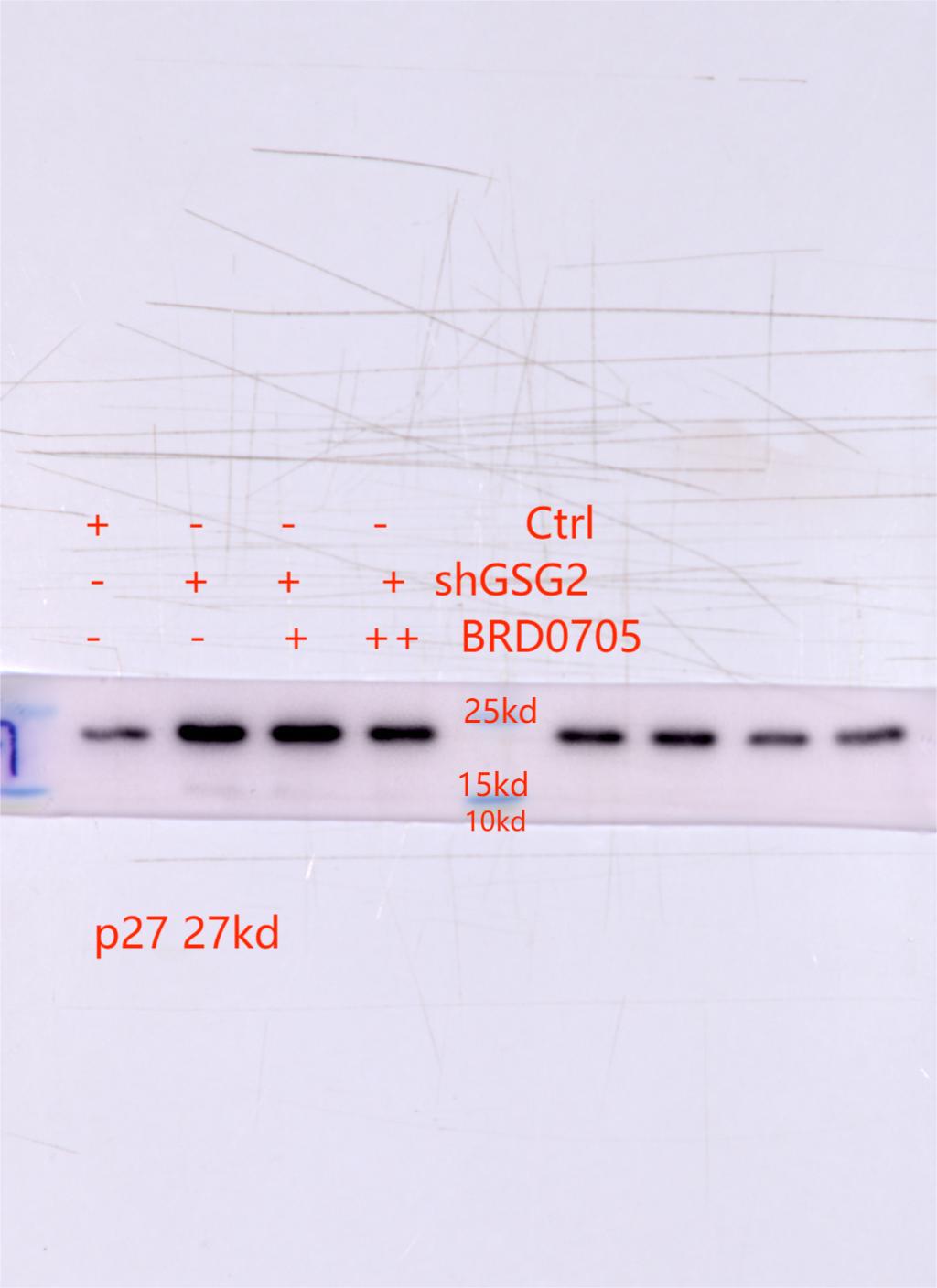

Supplement: Supplementary file 1 — Supplementary file1 (ZIP 11,586 kb) [file 10735_2024_10185_MOESM1_ESM.zip › 10735_2024_10185_MOESM1_ESM/Supplementary Material/Fig6A-HO8910-p27.jpg]

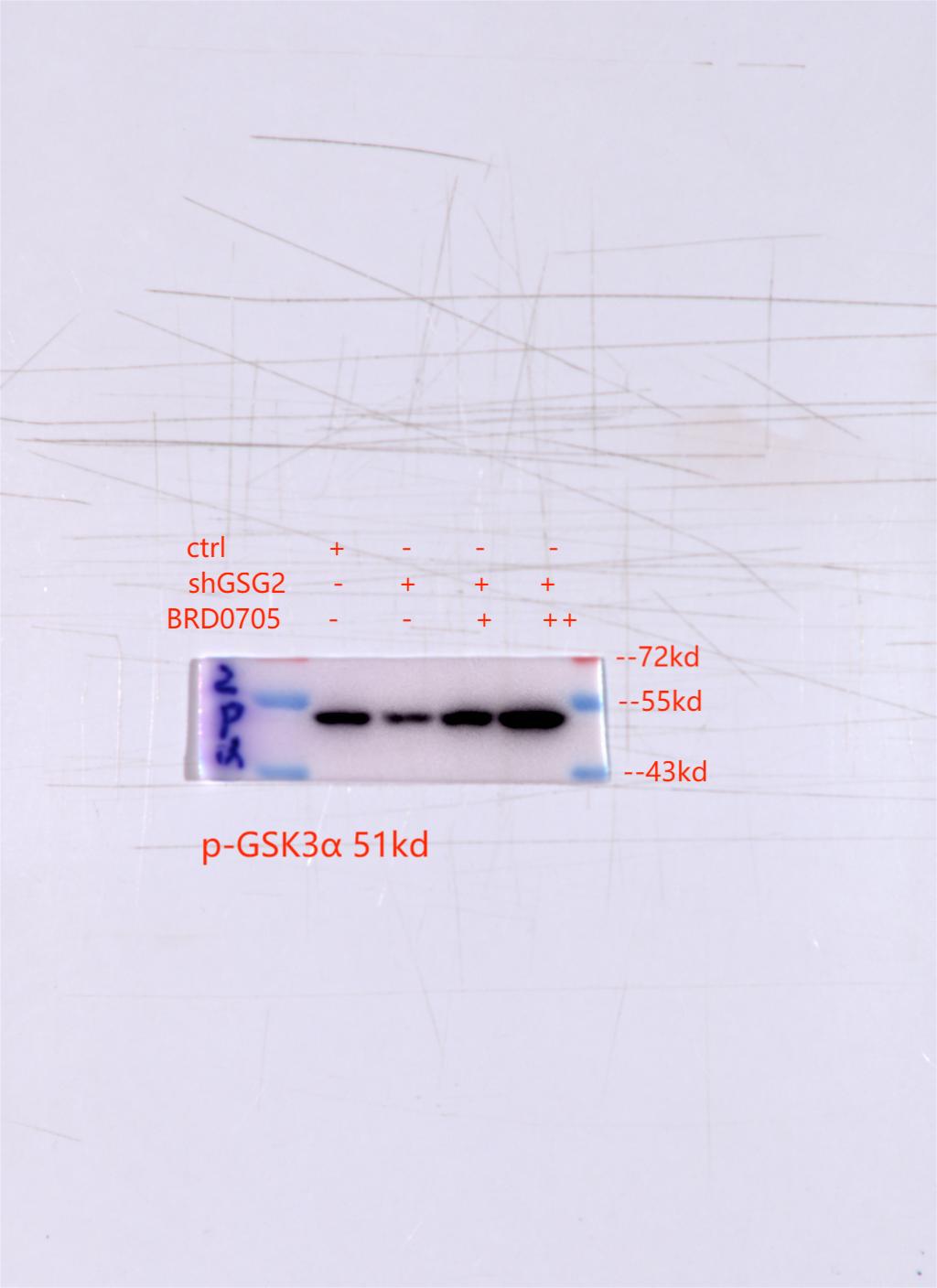

Supplement: Supplementary file 1 — Supplementary file1 (ZIP 11,586 kb) [file 10735_2024_10185_MOESM1_ESM.zip › 10735_2024_10185_MOESM1_ESM/Supplementary Material/Fig6A-HO8910-p-GSK3a .jpg]

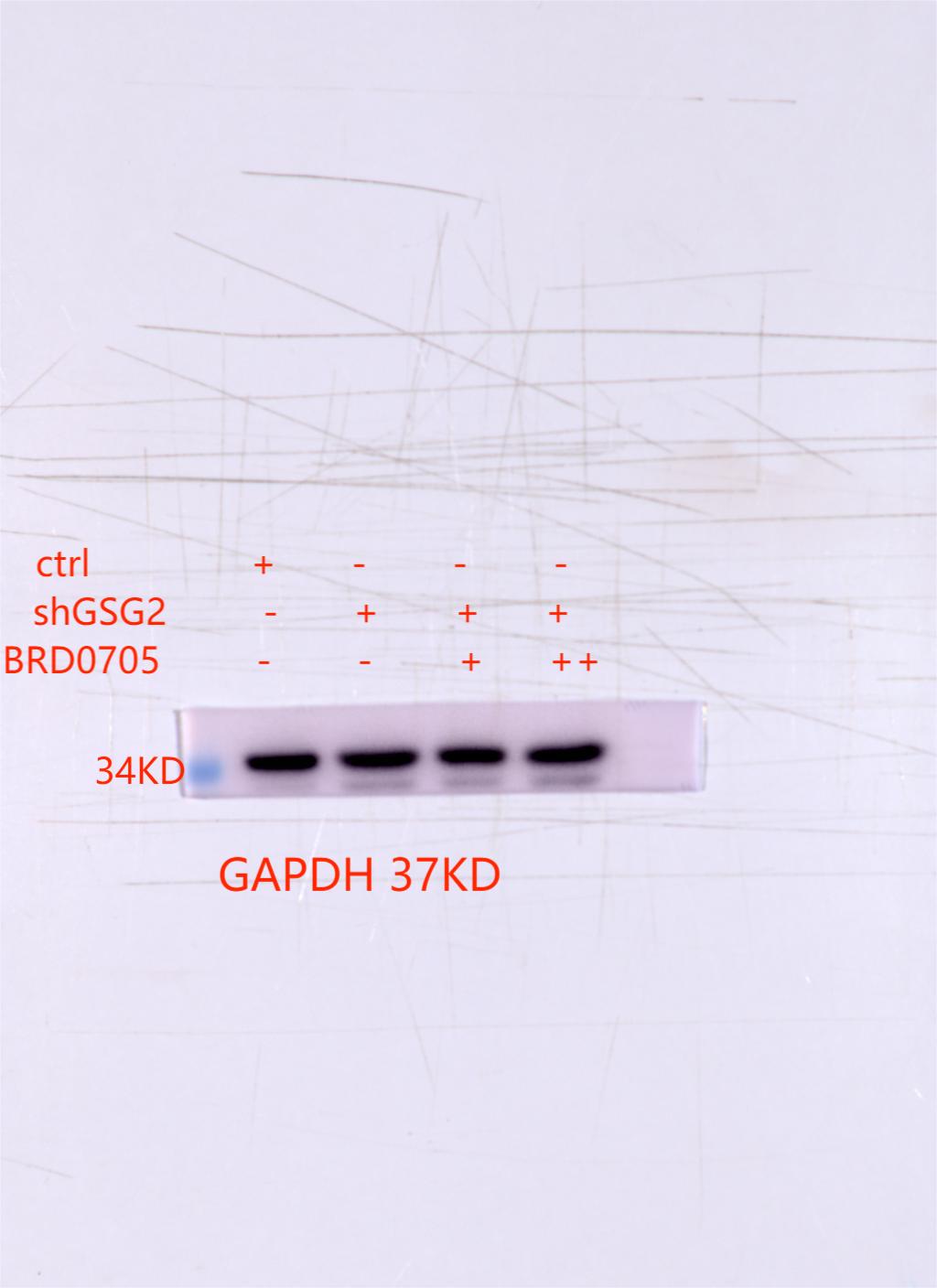

Supplement: Supplementary file 1 — Supplementary file1 (ZIP 11,586 kb) [file 10735_2024_10185_MOESM1_ESM.zip › 10735_2024_10185_MOESM1_ESM/Supplementary Material/Fig6A-SKOV3-GAPDH.jpg]

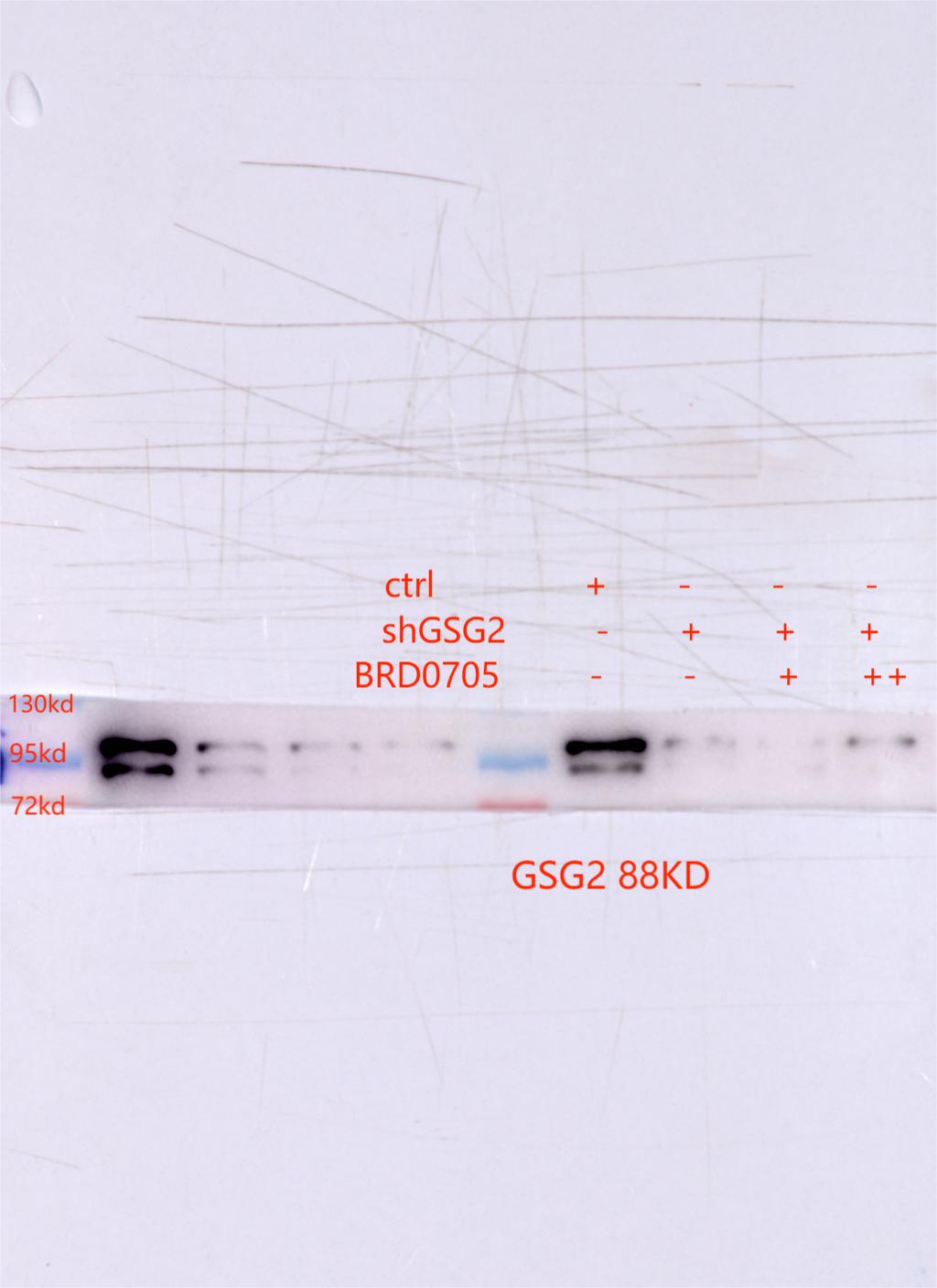

Supplement: Supplementary file 1 — Supplementary file1 (ZIP 11,586 kb) [file 10735_2024_10185_MOESM1_ESM.zip › 10735_2024_10185_MOESM1_ESM/Supplementary Material/Fig6A-SKOV3-GSG2.jpg]

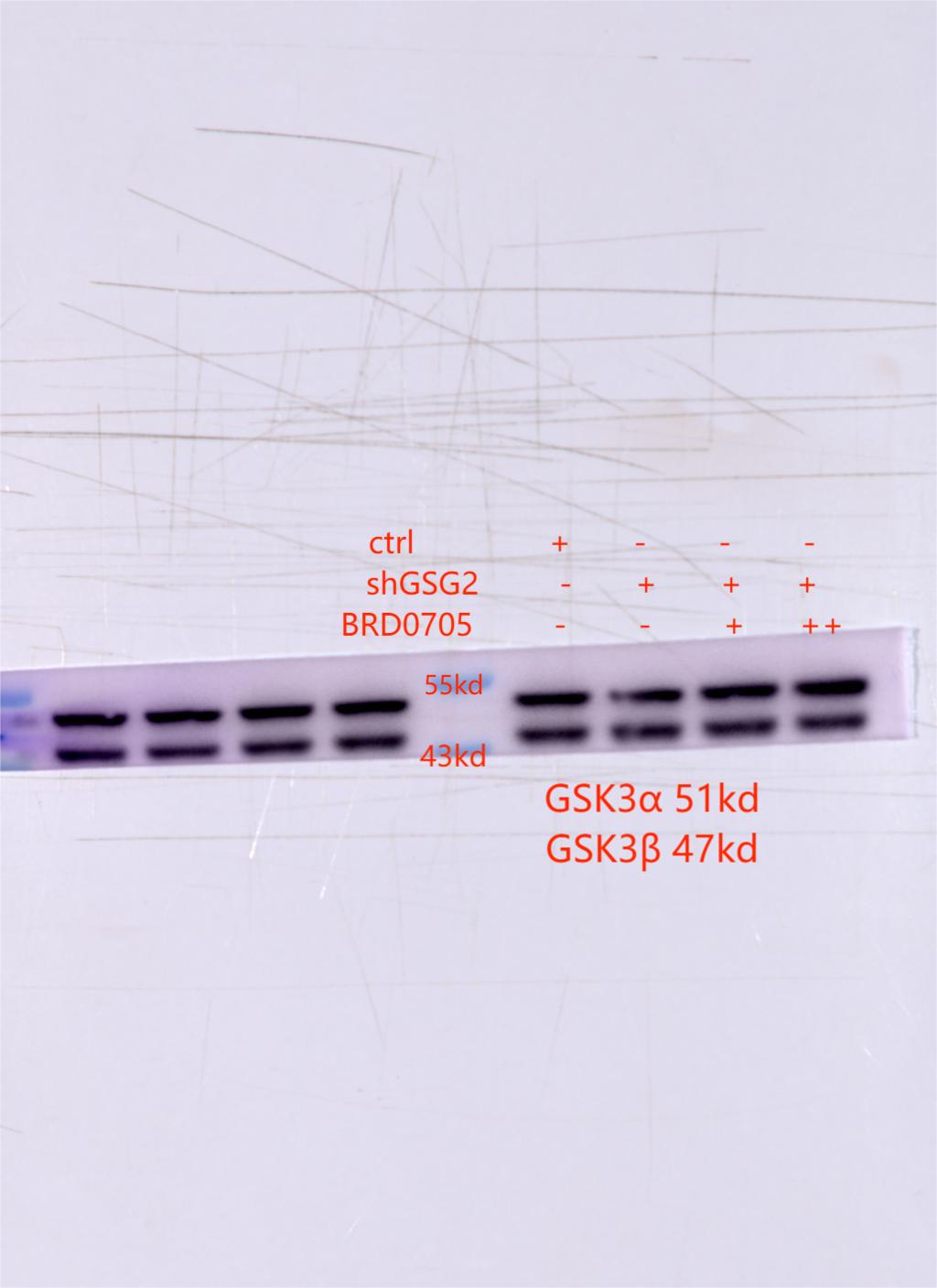

Supplement: Supplementary file 1 — Supplementary file1 (ZIP 11,586 kb) [file 10735_2024_10185_MOESM1_ESM.zip › 10735_2024_10185_MOESM1_ESM/Supplementary Material/Fig6A-SKOV3-GSK .jpg]

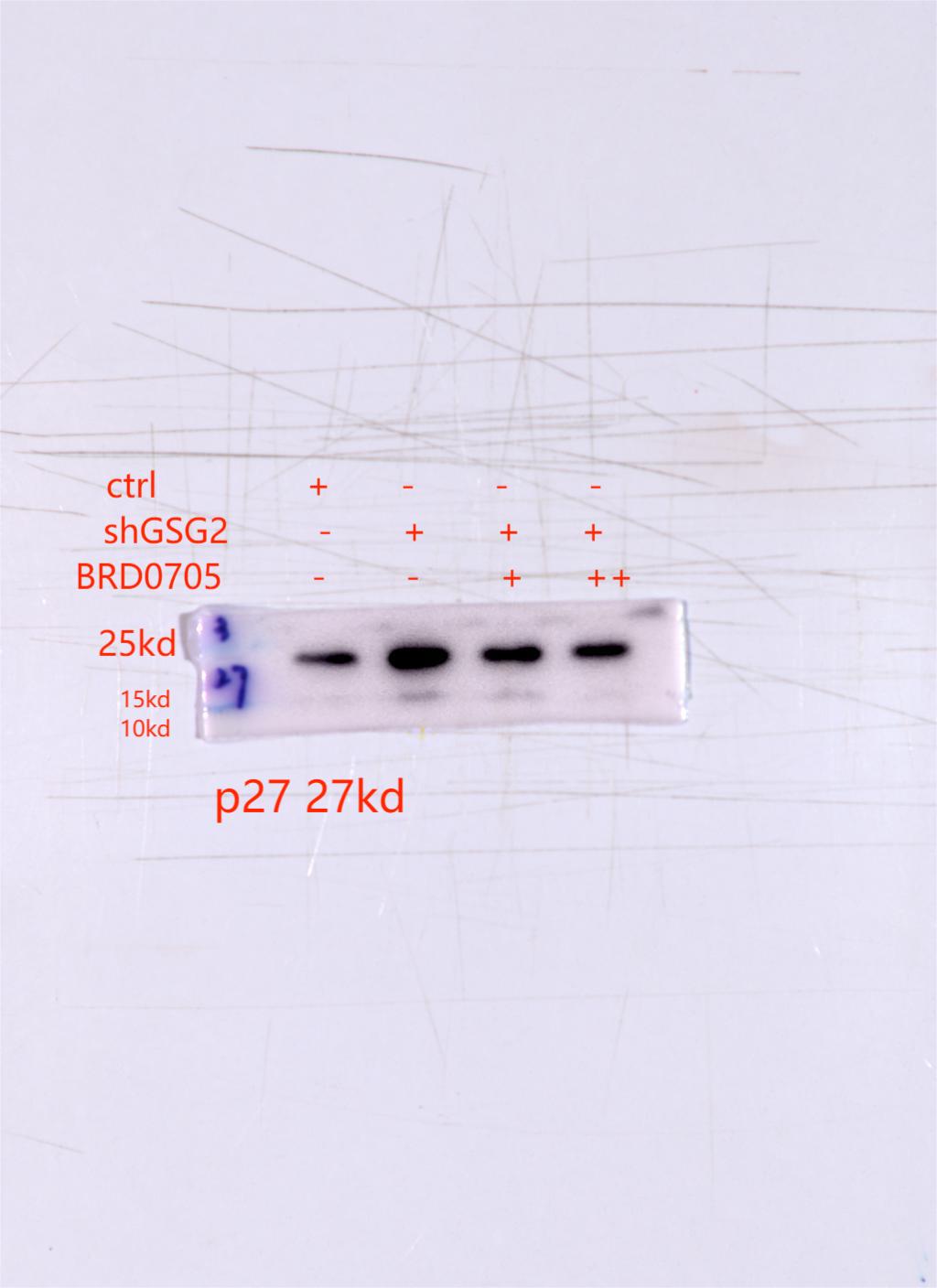

Supplement: Supplementary file 1 — Supplementary file1 (ZIP 11,586 kb) [file 10735_2024_10185_MOESM1_ESM.zip › 10735_2024_10185_MOESM1_ESM/Supplementary Material/Fig6A-SKOV3-p27.jpg]

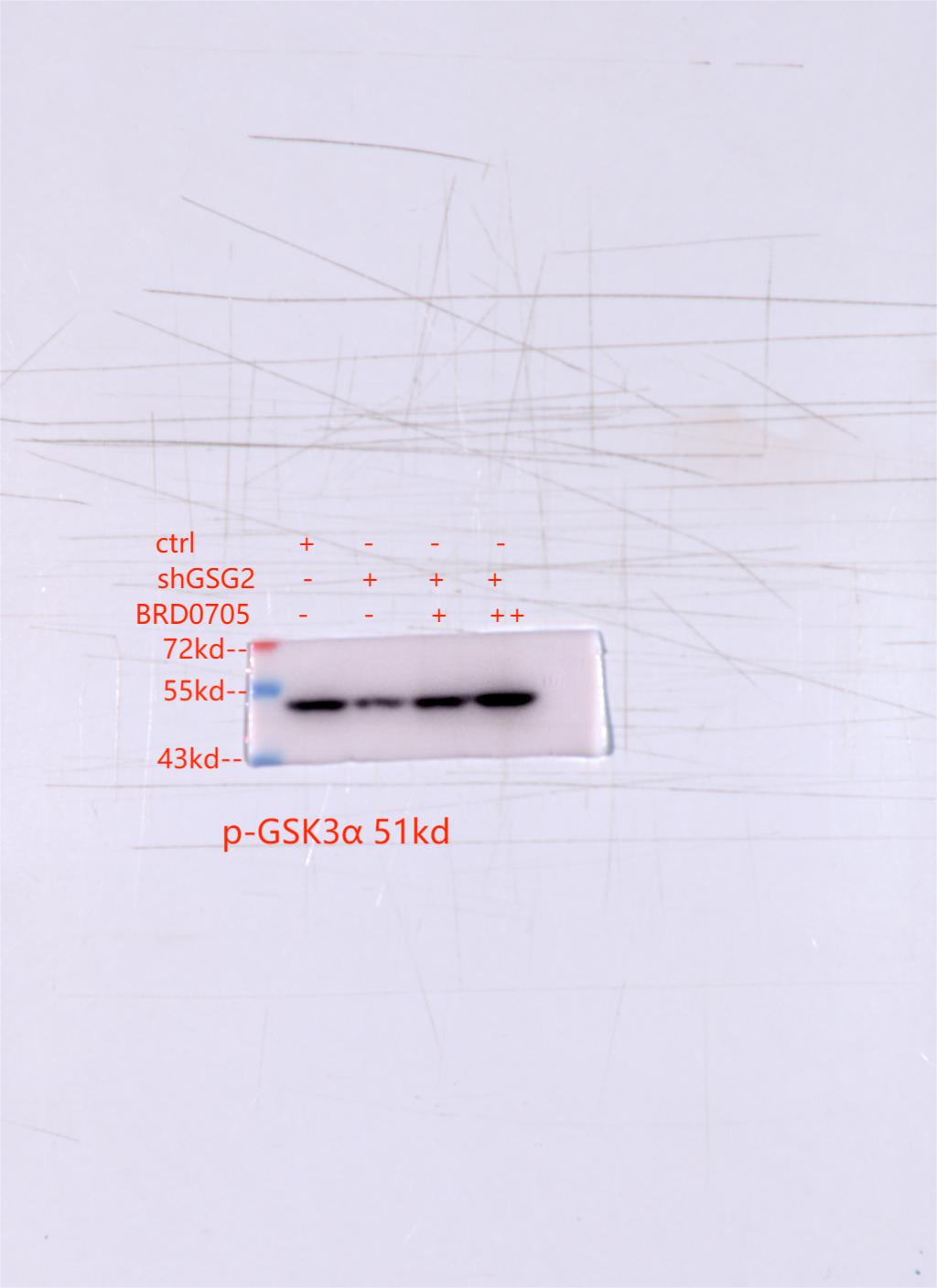

Supplement: Supplementary file 1 — Supplementary file1 (ZIP 11,586 kb) [file 10735_2024_10185_MOESM1_ESM.zip › 10735_2024_10185_MOESM1_ESM/Supplementary Material/Fig6A-SKOV3-p-GSK3a.jpg]

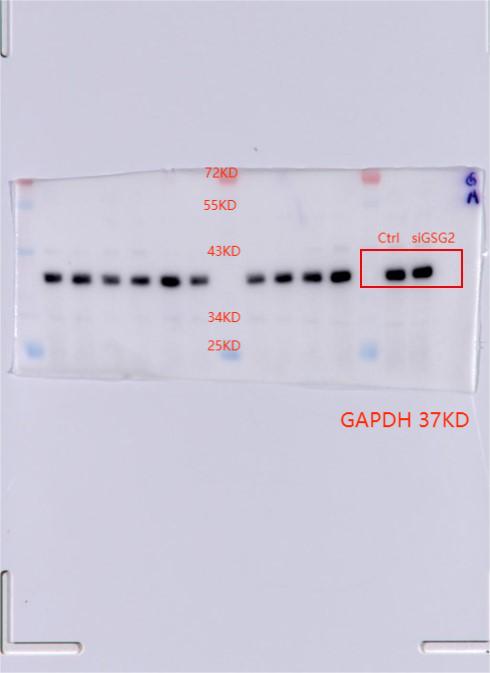

Supplement: Supplementary file 1 — Supplementary file1 (ZIP 11,586 kb) [file 10735_2024_10185_MOESM1_ESM.zip › 10735_2024_10185_MOESM1_ESM/Supplementary Material/Supplementary fig1A-A2780 GAPDH.jpg]

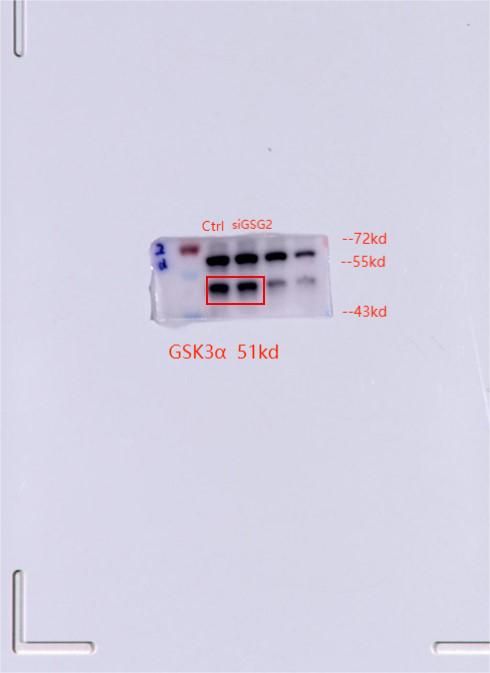

Supplement: Supplementary file 1 — Supplementary file1 (ZIP 11,586 kb) [file 10735_2024_10185_MOESM1_ESM.zip › 10735_2024_10185_MOESM1_ESM/Supplementary Material/Supplementary fig1A-A2780 GSKA.jpg]

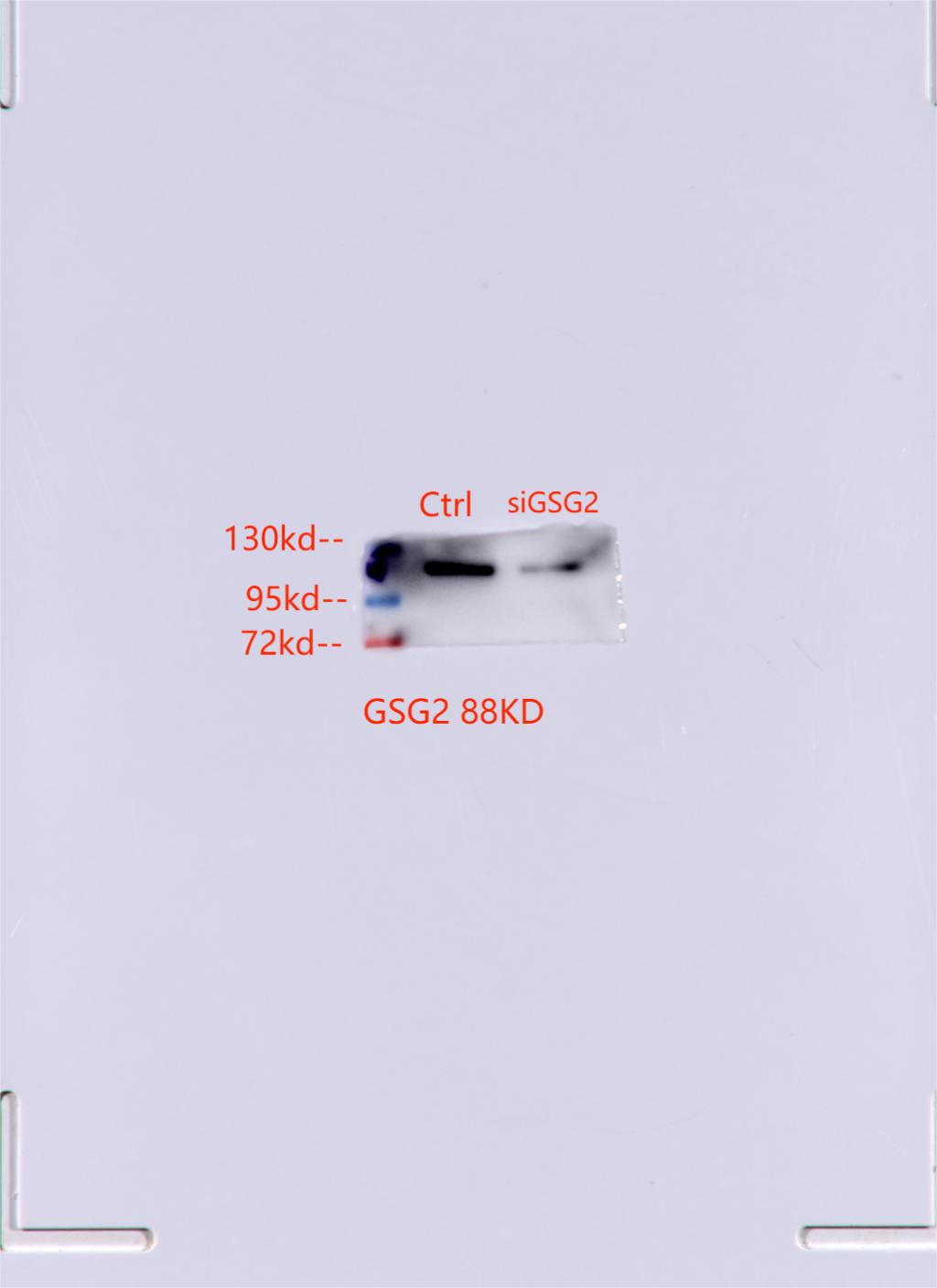

Supplement: Supplementary file 1 — Supplementary file1 (ZIP 11,586 kb) [file 10735_2024_10185_MOESM1_ESM.zip › 10735_2024_10185_MOESM1_ESM/Supplementary Material/Supplementary fig1A-A2780-GSG2 .jpg]

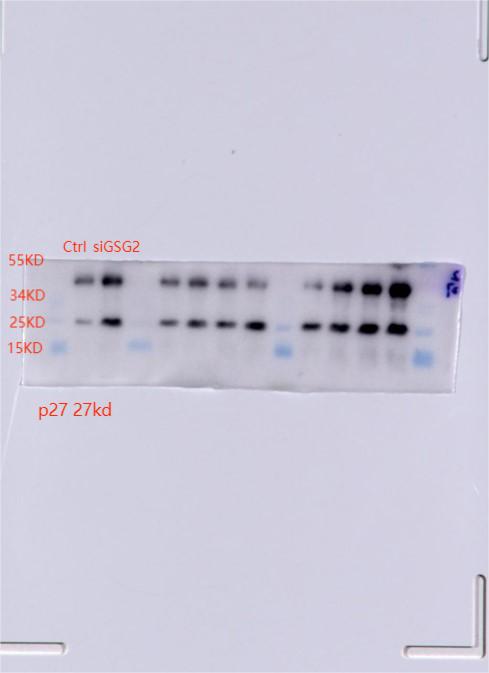

Supplement: Supplementary file 1 — Supplementary file1 (ZIP 11,586 kb) [file 10735_2024_10185_MOESM1_ESM.zip › 10735_2024_10185_MOESM1_ESM/Supplementary Material/Supplementary fig1A-A2780-P27 (1).jpg]

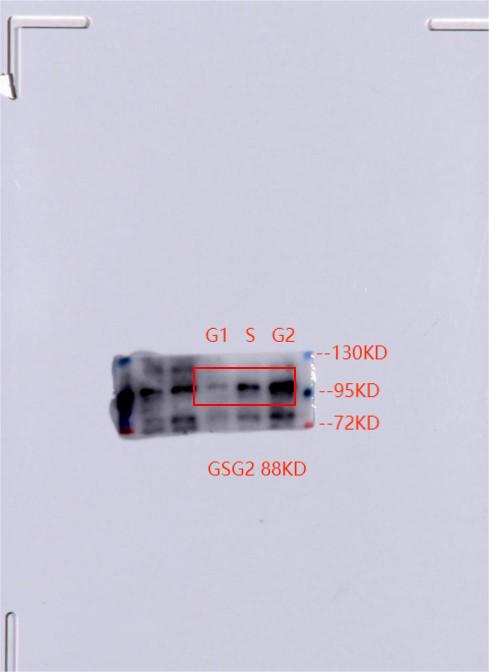

Supplement: Supplementary file 1 — Supplementary file1 (ZIP 11,586 kb) [file 10735_2024_10185_MOESM1_ESM.zip › 10735_2024_10185_MOESM1_ESM/Supplementary Material/Supplementary fig1A-A2780-P27 (2).jpg]

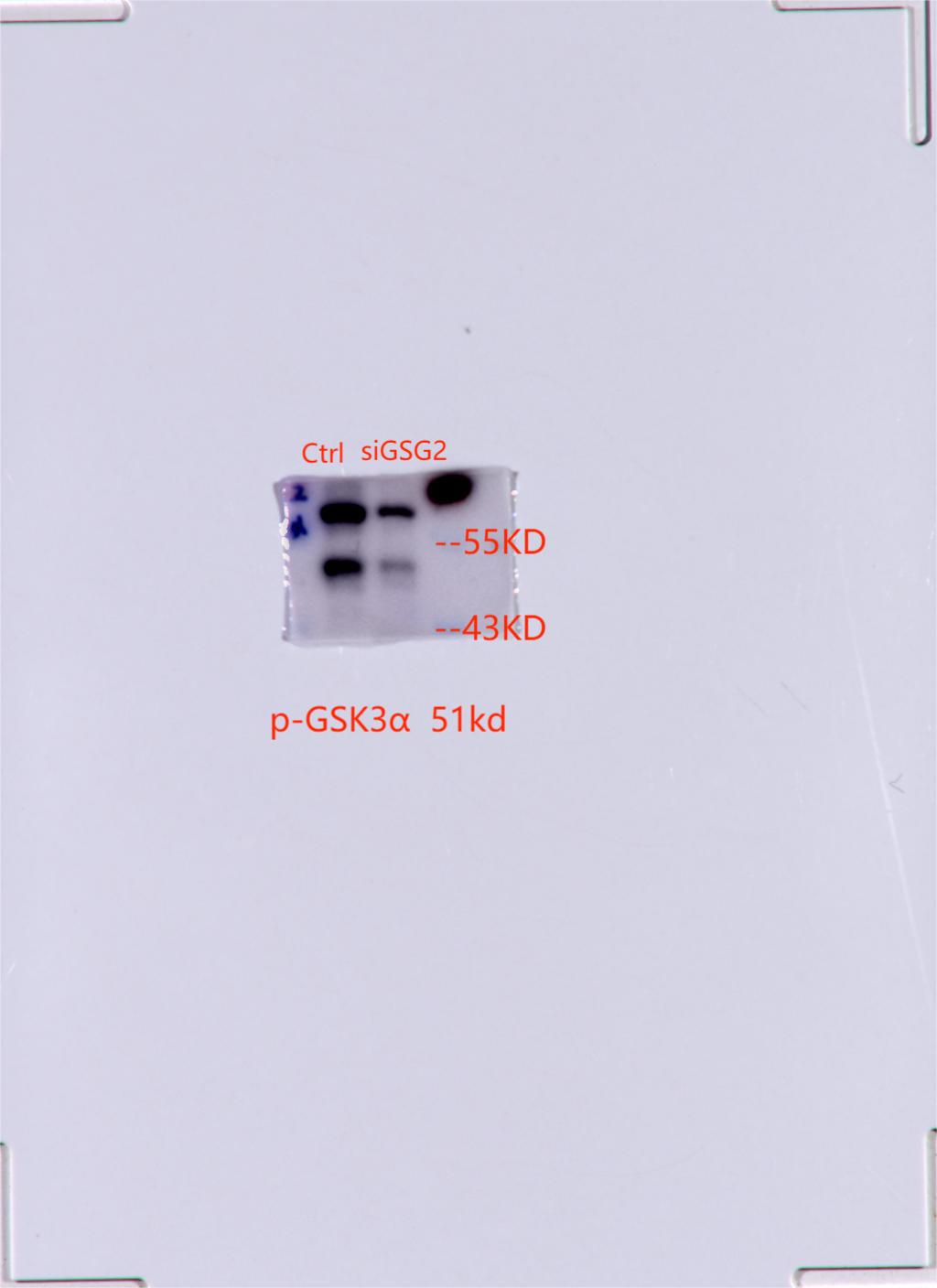

Supplement: Supplementary file 1 — Supplementary file1 (ZIP 11,586 kb) [file 10735_2024_10185_MOESM1_ESM.zip › 10735_2024_10185_MOESM1_ESM/Supplementary Material/Supplementary fig1A-A2780-P-GSK3a .jpg]

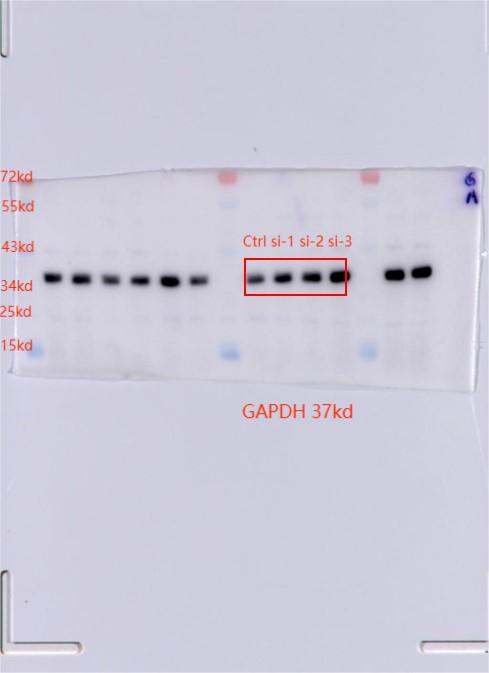

Supplement: Supplementary file 1 — Supplementary file1 (ZIP 11,586 kb) [file 10735_2024_10185_MOESM1_ESM.zip › 10735_2024_10185_MOESM1_ESM/Supplementary Material/Supplementary fig1A-SKOV3 GAPDH.jpg]
